# Global burden of active smoking among people living with HIV on antiretroviral therapy: a systematic review and meta-analysis

**DOI:** 10.1186/s40249-021-00799-3

**Published:** 2021-02-12

**Authors:** Boni Maxime Ale, Franck Amahowe, Motto Malea Nganda, Célestin Danwang, Nelly Njeri Wakaba, Ateeq Almuwallad, Franck Biaou Guy Ale, Alamou Sanoussi, Suleiman Hudu Abdullahi, Jean Joel Bigna

**Affiliations:** 1 Holo Healthcare Limited, Nairobi, Kenya; 2 Department of Clinical Science and International Public Health, Liverpool School of Tropical Medicine, Liverpool, UK; 3 Epidemiology and Biostatistics Unit, Institute of Experimental and Clinical Research, Université Catholique de Louvain, Brussels, Belgium; 4 Applied Medical Sciences College, Jazan University, Jazan, Saudi Arabia; 5 Center for Trauma Science, Queen Mary University of London, London, UK; 6 Transition Support Program Department, Advocate Good Samaritan Hospital, Downers Grove, IL USA; 7 Leprosy and Tuberculosis Relief Initiative Nigeria, Plateau, Nigeria; 8 Department of Epidemiology and Public Health, Centre Pasteur of Cameroon, Yaoundé, Cameroon

**Keywords:** Tobacco, Smoking, HIV, AIDS, Antiretroviral therapy, Global health

## Abstract

**Background:**

Although the high burden of both active smoking and human immunodeficiency virus (HIV) is clearly known, the relationship between them is still not well characterized. Therefore, we estimated the global prevalence of active smoking in people living with HIV (PLHIV) on antiretroviral therapy (ART) and investigated the association between exposure to active smoking and risk for suboptimal adherence to ART.

Main text: We searched PubMed, Embase, and Web of Science to identify articles published until September 19, 2019. Eligible studies reported the prevalence of active smoking in PLHIV on ART or investigated the association between active smoking and ART adherence; or enough data to compute these estimates. We used a random-effects model to pool data and quantified heterogeneity (*I*^2^). The global prevalence of active smoking was 36.1% (95% *CI*: 33.7–37.2; 329 prevalence data; 462 104 participants) with substantial heterogeneity. The prevalence increased with level of country income; from 10.1% (95% *CI*: 6.8–14.1) in low-income to 45.2% (95% *CI*: 42.7–47.7) in high-income countries; *P* < 0.0001. With regards to the Joint United Nations Programme on HIV/AIDS (UNAIDS) regions, the prevalence was higher in West and Central Europe and North America 45.4% (42.7–48.1) and lowest in the two UNAIDS regions of sub-Saharan Africa: Eastern and Southern Africa 10.7% (95% *CI*: 7.8–14.0) and West and Central Africa 4.4% (2.9–6.3); *P* < 0.0001. Globally, we estimated that there were 4 110 669 PLHIV on ART who were active smokers, among which the highest number was from Eastern and Southern Africa (35.9%) followed by Asia and the Pacific (25.9%). Active smoking was significantly associated with suboptimal ART adherence: pooled odds ratio 1.57 (95% *CI*: 1.37–1.80; *I*^2^ = 56.8%; 19 studies; 48 450 participants); even after considering adjusted estimates: 1.67 (95% *CI*: 1.39–2.01; *I*^2^ = 53.0%; 14 studies).

**Conclusions:**

This study suggests a high prevalence of active smoking in PLHIV on ART and an association between active smoking and ART suboptimal adherence. As such, healthcare providers and policy makers should focus on adopting and implementing tobacco harm reduction strategies in HIV care, especially in sub-Saharan Africa known as epicenter of HIV pandemic with highest number of active tobacco smoking among PLHIV on ART.

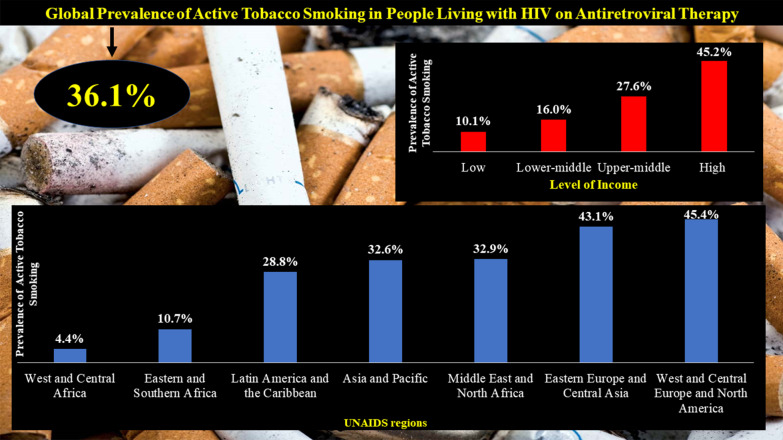

## Background

Human immunodeficiency virus (HIV) infection remains a global public concern. Globally, there were 37.9 million of people living with HIV (PLHIV) by the end of 2018 [1]. In this population, 24.5 million were accessing antiretroviral therapy (ART) [1, 2]. To curb the burden of HIV infection, the Joint United Program on HIV/AIDS (UNAIDS) set an ambitious goal that by 2030, 95% of all PLHIV should know their HIV status; 95% of people diagnosed with HIV infection should receive sustained ART; and 95% of all people receiving ART should have ribonucleic acid HIV suppression [3]. Since non-adherence to ART is correlated with poor virological response to HIV treatment [4], PLHIV should be voluntary compliant to the prescribed ART to achieve the third 95.

Active tobacco smoking could be one of the factors which would prevent reaching the goal. However, to date, it is not clear whether active smoking could be associated with suboptimal adherence to ART in PLHIV. Globally, tobacco smoking is one of the biggest public health threats the world has ever faced, killing more than 8 million people annually [5]. In addition, tobacco is one of the leading risk factors for cardiovascular diseases, the top killer worldwide [6]. In 2018, 770 000 people died from AIDS-related illnesses [1, 2]. In addition, HIV infection is now recognized as risk factor of cardiovascular diseases (the top killer worldwide) alongside with traditional cardiovascular risk factors [6].

Although the high burden of both active smoking and HIV is clearly known, the relationship between them and the burden of active smoking in PLHIV on ART are still not well characterized. Accurate global and context-specific epidemiological data are crucial to tackle the burden of active smoking in PLHIV, especially for health-care planning and resource allocations. Hence, we conducted this systematic review with meta-analysis to determine the burden of active smoking in PLHIV undergoing ART. Firstly, we estimated the global prevalence of active smoking in PLHIV on ART and estimated this prevalence by UNAIDS region and country level of income. Secondly, we provided the burden of active smoking in terms of number of smokers in PLHIV on ART at global and UNAIDS regional levels. Finally, we investigated the association between exposure to active smoking and risk for suboptimal adherence to ART in PLHIV.

## Methods

### Eligibility criteria

We used the same method as per already published meta-analyses [7, 8]. This systematic review and meta-analysis was conducted according to the Joanna Briggs Institute guidelines [9]. This study was reported according to the PRISMA guidelines [10]. The protocol of this review was registered in PROSPERO, CRD42020195796.

We considered observational studies including cross sectional, case-control, and cohort studies. We considered studies reporting the prevalence (or enough data to compute this estimate) of current or active tobacco smoking (willful or deliberate act of inhaling and exhaling smoke from burning substances or agents held by hand) in the global population of PLHIV undergoing ART (at least 90% of PLHIV with ART) [11]. We also considered studies investigating the association between active tobacco smoking and ART suboptimal adherence in PLHIV.

### Search strategy

We searched PubMed, Excerpta Medica Database (EMBASE), and Web of Science to identify all relevant records published up to September 19^th^, 2019 without any language restriction. The search strategy in EMBASE is available in the Appendix (Supplementary Table 1). This search was adapted to suit with other databases. To supplement the bibliographic database searches and identify potential additional data sources, we scrutinized the reference list of all relevant review papers.

**Table 1:** Meta-analysis prevalence of active smoking in people living with HIV undergoing antiretroviral therapy

	**Prevalence, %**	**95% confidence intervals**	**No. studies**	**No. participants**	**Heterogeneity**		**Egger test**	***P value, difference***
					***I***^**2**^**, %**	***P value***		
Global	36.1	33.7–38.5	329	462 104	99.5	< 0.0001	0.0002	…
All participants on ART	34.5	31.8–37.2	268	420 575	99.6	< 0.0001	0.0001	…
Country level of income								
Low	10.1	6.8–14.1	30	11 329	95.9	< 0.0001	0.095	< 0.0001
Lower-middle	16.0	8.6–25.3	21	9858	98.7	< 0.0001	0.496	
Upper-middle	27.6	23.8–31.6	39	8252	90.4	< 0.0001	0.668	
High	45.2	42.7–47.7	216	339 022	99.5	< 0.0001	0.527	
UNAIDS region								
West and Central Africa	4.4	2.9–6.3	12	4868	87.0	< 0.0001	0.263	< 0.0001
Eastern and Southern Africa	10.7	7.8–14.0	34	12 676	94.8	< 0.0001	0.080	
Latin America and the Caribbean	28.8	25.0–32.8	24	16 385	95.9	< 0.0001	0.162	
Asia and Pacific	32.6	26.6–38.9	27	16 352	97.3	< 0.0001	0.031	
Middle East and North Africa	32.9	16.1–52.4	3	320	93.0	< 0.0001	0.711	
Eastern Europe and Central Asia	43.1	15.8–72.9	2	264	95.9	< 0.0001	NA	
West and Central Europe and North America	45.4	42.7–48.1	203	293 547	99.5	< 0.0001	0.339	

*HIV* Human immunodeficiency virus, *ART* antiretroviral therapy, *UNAIDS* Joint United Program on HIV, *NA* not applicable

### Study selection

Titles and abstracts of articles retrieved from literature search were independently screened by two investigators, and the full-texts of those potentially eligible were obtained and further assessed for final inclusion. Disagreements were resolved through consensus.

### Data extraction and management

A preconceived and standardized data extraction form was used to collect information on first author’s name, study country, year of publication, period of participants’ recruitment, study design, setting, sampling method, timing of data collection, response rate, mean or median age of the population, proportion of males, proportion with antiretroviral treatment, number of PLHIV, the number of participants with active smoking. For studies investigating the association between active tobacco smoking and adherence to antiretroviral therapy, we also extracted data for participants with no active smoking, data on the adherence as defined in the original studies, and variables of adjustment in multivariable analysis. Studies were classified by level of income according to the country of recruitment of participants as per World Bank classification [12]. Ten investigators independently extracted data from individual studies, with disagreements being resolved through discussion. Ten investigators independently assessed methodological quality of included studies with tool developed by Joanna Briggs Institute for prevalence data and with Newcastle Ottawa Scale for comparative studies investigating the association between active tobacco smoking and ART adherence [9, 13]; with disagreements being resolved through discussion.

### Data synthesis and analysis

Meta-analyses were performed with the *meta, metafor,* and *dmetar* packages of the statistical software *R* (version 3.6.3, R foundation for Statistical Computing, Vienna, Austria). Prevalence estimates were reported with 95% confidence interval (95% *CI*). Prevalence pooling was done with single arcsine transformation using random-effects meta-analysis model [14]. Sensitivity analyses was performed including only studies with low risk of bias for each of defined items. We estimated an adjusted global prevalence taking in account the variability between UNAIDS region and country level of income. Publication bias was investigated with funnel plot and confirmed with the Egger’s test [15]. A *P*-value < 0.10 on the Egger test was considered indicative of statistically significant publication bias. We performed meta-regression analysis and estimated the variance explained by study and participants’ characteristics. In this analysis, the outcome of interest was the prevalence of active smoking in patients undergoing ART, included as a continuous variable. The final multivariable model was chosen based on the lowest corrected Akaike’s Information Criterion. For missing data, we have performed a non-parametric test of homoscedasticity and there was not sufficient evidence to reject that data were missing completely at random, *P* = 0.450. Therefore, we have performed multiple-imputation analyses using chained equations for studies who had a missing value. We generated 40 imputed-data sets with a maximum number of 10 iterations, with linear imputation for continuous variables [16]. Variables that were included in the imputation model were proportion of males, mean age, duration of since HIV diagnosis and duration on ART. We have also performed a meta-regression analysis with complete cases.

To measure the association between active tobacco smoking and non-adherence to ART, we did a meta-analysis using the random-effects method to pool weighted (adjusted) odds ratios (*OR*) of suboptimal ART adherence risk estimate. The symmetry of funnel plot was used to explore publication bias and Harbord test was done to assess the presence of publication bias [17].

Heterogeneity was evaluated by the *χ*^2^ test on Cochran’s Q statistic [18], which was quantified by *I*^*2*^ values. The *I*^*2*^ statistic estimates the percentage of total variation across studies due to true difference between-study differences rather than chance. In general, *I*^*2*^ values greater than 60–70% indicate the presence of substantial heterogeneity [19].

## Results

### Study selection and characteristics

Of the 2329 records identified, we included 290 studies with 329 prevalence data for estimating the prevalence of active smoking in PLHIV on ART and 19 studies for investigating the association between active tobacco smoking and ART adherence (Additional file 1: Figure S1).

Of the 290 studies included in the meta-analysis of prevalence, most of them were cross-sectional, hospital-based, and published between 2000 and 2019 (Additional file 1: Tables S2 and S3). According to the methodological quality, most of studies used non-probabilistic sampling, had low precision, had unclear description of response rate, were prospectively collected data, and used identical procedure to collect data among participants (Additional file 1: Tables S2 and S3).

Of the 19 studies included in the meta-analysis for the association between active tobacco smoking and adherence to ART, most of them were cross-sectional and hospital-based, had low risk of bias on the selection of participants, included comparable groups and outcome assessment, and were published between 2006 and 2019 (Additional file 1: Tables S4 and S5). All studies used non-probabilistic sampling.

### Prevalence of active tobacco smoking in the global population living with HIV

Of the 329 prevalence data, 216 (65.7%) were from high, 39 (11.9%) from upper-middle, 21 (6.4%) from lower-middle, and 30 (9.1%) from low-income countries. Twenty-three (7.0%) were not disagreeable. According to the UNAIDS regional distribution, 203 (61.7%) prevalence data were from West and Central Europe and North America, 34 (10.3%) from Eastern and Southern Africa, 27 (8.2%) form the Asia and Pacific, 24 (7.3%) from Latin America and the Caribbean, 12 (3.7%) from West and Central Africa, 3 (0.9%) from Middle-East and North Africa, and 2 (0.6%) from Eastern Europe and Central Asia. Twenty-four (7.3%) prevalence data were multiregional. According to participants’ characteristics, the mean or median age varied from 16.7 to 66 years (*n* = 290), the proportion of males varied from 0 to 100% (*n* = 301), the duration since HIV diagnosis varied from 0.5 to 22 years (*n* = 119), and the duration on ART varied from 0.7 to 18.4 years (*n* = 98).

The global prevalence of active smoking in a total of 462 104 PLHIV undergoing ART was 36.1% (95% *CI*: 33.7–37.2) with substantial heterogeneity (Table 1). There was asymmetry on the funnel plot corroborated by the Egger test (Additional file 1: Figure S2; Table 1). The prevalence from sensitivity analyses including studies with all patients undergoing ART, and only studies with low risk of bias were close to crude prevalence (Additional file 1: Tables S6). Although in the range of the global crude prevalence, the adjusted prevalence taking in account the variance between country level of income and UNAIDS regions was slightly lower: 27.3% (16.9–39.2).

## Sources of heterogeneity of the global prevalence

**Fig. 1:**
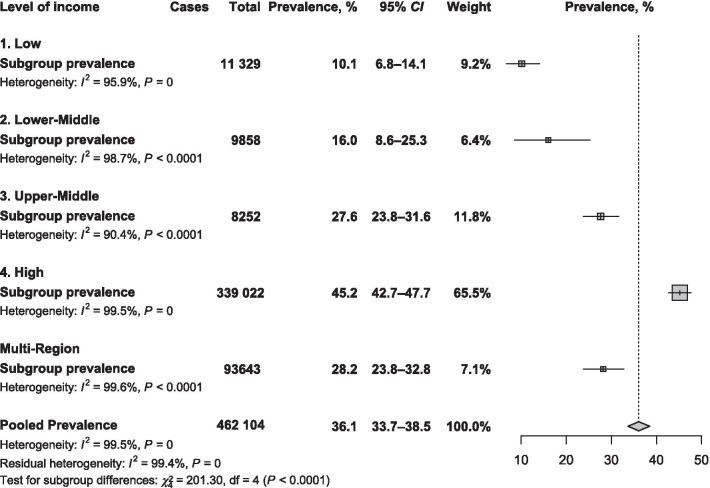
Meta-analysis prevalence of active smoking in people living with HIV on antiretroviral therapy by country level of income

**Fig. 2:**
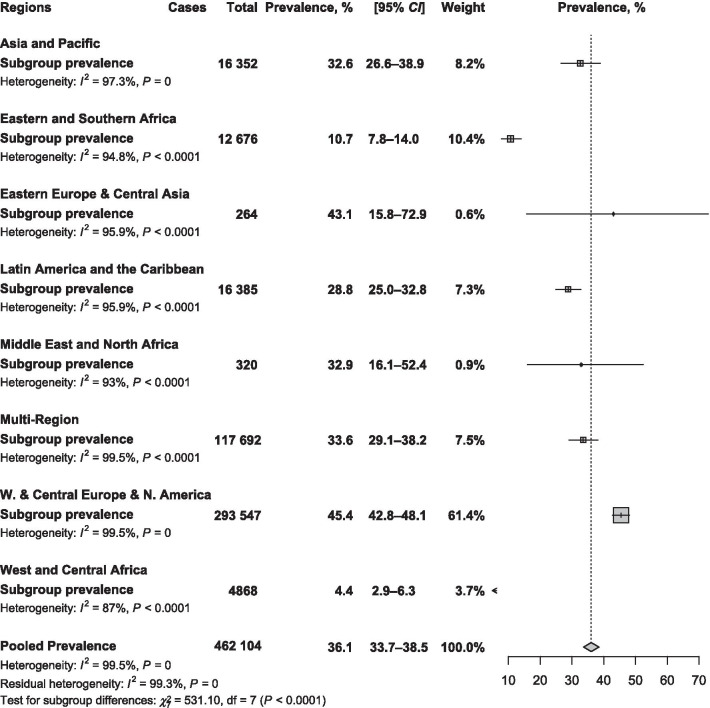
Meta-analysis prevalence of active smoking in people living with HIV on antiretroviral therapy by UNAIDS region

**Fig. 3:**
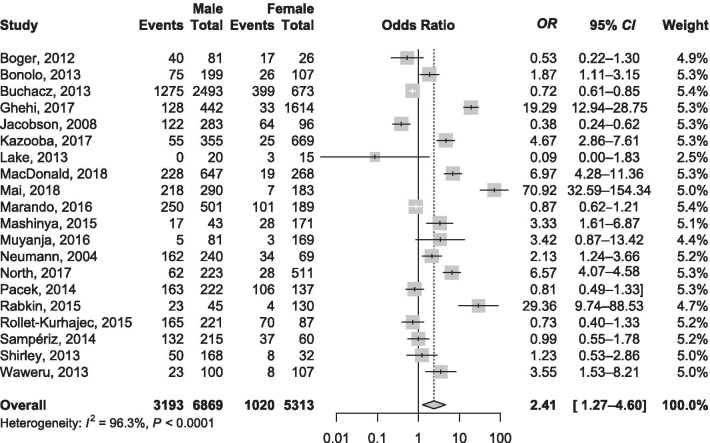
Association between male sex and active smoking in people living with HIV on antiretroviral therapy

In subgroup analysis, the prevalence increased with level of country income; from 10.1% (6.8–14.1) in low-income countries to 45.2% (42.7–47.7) in high-income countries, *P* < 0.0001 (Fig. 1). According to the UNAIDS region, the lowest prevalence was in sub-Saharan Africa regions: West and Central Africa 4.4% (2.9–6.9) and Eastern and Southern Africa 10.7% (7.8–14.0) and the highest prevalence was in West and Central Europe and North America 45.4% (42.7–48.1), *P* < 0.0001 (Fig. 2; Additional file 1: Figures S3 to S9). Males were more likely to smoke than females: pooled *OR* = 2.41 (95% *CI*: 1.27–4.60; *I*^2^ = 96.3%; 20 studies) (Fig. 3).

In the univariable meta-regression analysis, the explained variance in the global prevalence varied from 0.1% for response rate to 45.3% for UNAIDS region (Table 2). The variation in the global prevalence significantly increased with country level of income, proportion of males, duration since HIV infection diagnosis, duration on ART, increasing age, and was associated with UNAIDS region (Table 2). There was no difference with the analysis considering complete cases (Additional file 1: Tables S7).

**Table 2:** Meta-regression analysis of sources of heterogeneity and factors associated with the variation of prevalence of active tobacco smoking in people living with HIV ongoing antiretroviral therapy

	**No. studies**	**No. participants**	**Univariable model**			**Multivariable model**				
	***n = 329***	***n = 462 104***	**Prevalence difference (95% confidence intervals)**	***P value***	**R**^**2**^, %	**Adjusted prevalence difference (95% confidence intervals)**		***P value***		**LR test**
Country level of income				< 0.0001	39.4					0.023
Low	30	11 329	Ref			Ref				
Lower-Middle	21	9858	9.1 (− 1.4 to 20.7)	0.091		− 0.7 (− 4.3 to 3.0)		0.916		
Upper-Middle	39	8252	25.9 (15.4 to 37.2)	< 0.0001		10.0 (6.3 to 13.9)		0.092		
High	216	339 022	51.2 (41.1 to 62.0)	< 0.0001		13.8 (9.2 to 18.6)		0.047		
Multi-region	23	93 643	26.4 (14.7 to 39.4)	< 0.0001		− 2.0 (− 6.3 to 2.5)		0.776		
UNAIDS				< 0.0001	45.3					< 0.0001
West and Central Europe and North America	203	293 547	Ref			Ref				
West and Central Africa	12	4 868	− 40.9 (− 46.5 to − 34.6)	< 0.0001		− 32.0 (− 35.1 to − 28.8)		< 0.0001		
Eastern and Southern Africa	34	12,676	− 33.3 (− 37.4 to − 29.0)	< 0.0001		− 26.0 (− 28.9 to − 22.9)		< 0.0001		
Eastern Europe and Central Asia	2	264	− 2.2 (− 23.3 to 24.6)	0.865		5.5 (− 2.6 to 14.4)		0.650		
Latin America and the Caribbean	24	16 385	− 16.0 (− 21.9 to − 9.6)	< 0.0001		− 13.9 (− 17.1 to − 10.6)		0.020		
Asia and the Pacific	27	16 352	− 12.4 (− 18.2 to − 6.0)	0.0002		− 4.9 (− 7.6 to − 2.1)		0.388		
Middle East and North Africa	3	320	− 12.0 (− 28.1 to 7.6)	0.212		− 10.9 (− 17.1 to − 4.3)		0.401		
Multi− region	24	117 692	− 11.5 (− 17.7 to − 4.9)	0.0009		− 4.0 (− 7.1 to − 0.9)		0.622		
Percentage of males					13.4					
Increase of 10%	301*	446 434	3.3 (2.2 to 4.3)	< 0.0001						
Duration since diagnostic infection					5.7					
By increase of 5 years	119*	68 194	6.4 (3.0 to 10.0)	0.0004						
Duration of antiretroviral therapy					0.8					
By increase of 5 years	98*	117 811	4.1 (0.5 to 7.9)	0.0285						
Mean age					4.0					
By increase of 10 years	290*	380 280	6.0 (2.2 to 10.1)	0.0002						
Response rate					0.1					
< 80% or not described	172	268 253	Ref							
≥ 80%	157	193 851	− 1.2 (− 6.1 to 3.8)	0.628						
Sampling method					0.9					
Non probabilistic	277	436 880	Ref							
Probabilistic	52	25 224	− 5.5 (− 11.8 to 1.1)	0.102						
Timing of data collection and analysis				0.238	1.3					
Prospectively	261	317 315	Ref							
Retrospectively	50	129 628	4.6 (− 2.4 to 12.2)	0.205						
Both	1	957	42.4 (− 8.7 to 122.2)	0.119						
Unclear	17	14 204	4.0 (− 7.1 to 16.5)	0.493						
Precision					0.8					
Low	197	30 651	Ref							
Acceptable	132	431 453	− 4.1 (− 8.9 to 0.8)	0.102						

*ART* Antiretroviral therapy, *UNAIDS* Joint United Program on HIV, *NA* Not applicable, *LR* Likelihood ratio

*Missing data were imputed

In the final multivariable meta-regression model, the global prevalence increased with country level of income. The prevalence was lower in Eastern and Southern Africa, in West and Central Africa, in Latin America and the Caribbean compared to West and Central Europe and North America. There was no difference for other regions. These two variables accounted for 47.3% of the variation in the global prevalence (Table 2). The same variables were included in the final model considering completes cases (Additional file 1: Tables S7).

### Burden of active tobacco smoking in people undergoing antiretroviral treatment

Considering regional estimates, 35.9% of PLHIV undergoing ART were from Eastern and Southern Africa, followed by Asia and Pacific (25.4%) and West and Central Europe and North America (18.8%). Globally, 4 110 669 PLHIV undergoing ART were active smokers (Table 3).

**Table 3:** Global and regional estimates of active smoking in people living with HIV undergoing antiretroviral therapy

Regions	Estimated number of PLHIV undergoing ART with active smoking	Proportion among total regional estimable cases
Global	4 110 669	…
Eastern and Southern Africa	1 476 600	35.9%
Asia and the Pacific	1 043 200	25.4%
West and Central Europe and North America	771 800	18.8%
Latin America and the Caribbean	399 456	9.7%
Eastern Europe and Central Asia	279 288	6.8%
West and Central Africa	114 400	2.8%
Middle East and North Africa	25 925	0.6%

*HIV* Human Immunodeficiency Virus, *PLHIV* People living with HIV, *ART* Antiretroviral treatment

### Active tobacco smoking and adherence to antiretroviral therapy

**Fig. 4:**
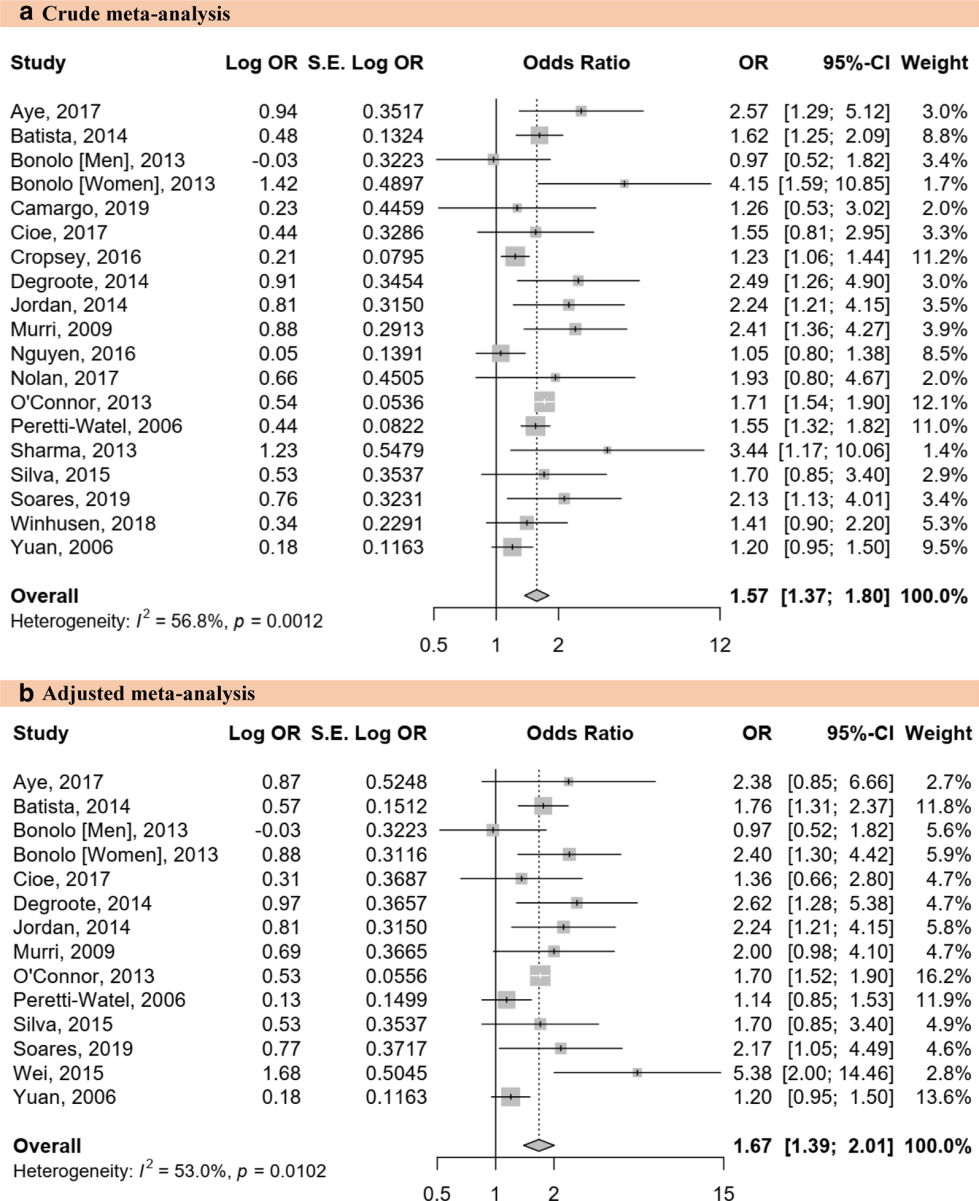
Association between active smoking and suboptimal adherence to antiretroviral therapy in people living with HIV. **a** Crude estimate, **b** Adjusted estimate

We included 19 studies with 48 450 PLHIV undergoing ART in the meta-analysis for investigating the association between active tobacco smoking and suboptimal adherence to ART. There was clinical heterogeneity in the definition of adherence to ART across included studies (Additional file 1: Tables S5). The risk for suboptimal adherence to ART was significantly higher in patients with active tobacco smoking than in non-active smoking: pooled *OR* = 1.57 (95% *CI*: 1.37–1.80; *I*^2^: 56.8%; 19 studies) (Fig. 4a). There was no asymmetry on the funnel plot (Additional file 1: Fig. S10), confirmed by the Harbord test (*P* = 0.293). The meta-analysis of adjusted *OR* yielded a pooled estimate of 1.67 (95% *CI*: 1.39–2.01; *I*^2^ = 53.0%; 14 studies) (Fig. 4b). There was no asymmetry on the funnel plot (Additional file 1: Fig. S11), confirmed by the Harbord test (*P* = 0.410).

## Discussion

In this meta-analysis of 329 prevalence data with 462 104 PLHIV on ART, we found that more than third of them actively smoke tobacco. The prevalence of active tobacco smoking in PLHIV in ART was higher in Western & Central Europe and North America UNAIDS region. However, the burden in terms of number of PLHIV on ART that actively smoke was higher in Eastern and Southern Africa. The prevalence increased with country level of income and was higher among males compared to females. We also found that active smoking was associated with suboptimal ART adherence.

The prevalence of active smoking in PLHIV on ART found in this study is close to the one reported among females in a previous meta-analysis of 51 studies (36.3%, 95% *CI*: 28.0–45.4%) and slightly lower compared to the one reported among males (50.3%, 95% *CI*: 44.4–56.2% in the same study [20]. Another meta-analysis of 31 studies found a prevalence 46.5% (95% *CI*: 40.9–52.1) among PLHIV regardless of their ART status [21]. Our review included more studies and focused on PLHIV undergoing ART. In addition, our review also represents a more contemporary and global estimate. Like in the general population [22, 23], the odds ratio of active smoking was higher among males compared to females in this study. In the global general population, about 40% of men smoke as compared to nearly 9% of women [22]. Indeed, results from neuroimaging studies suggest that smoking activates men’s reward pathways more than women [24]. This is reflected by higher odds of smoking among men compared to women in this meta-analysis and other meta-analyses of PLHIV regardless of their ART status [20, 21].

The prevalence increased with country level of income. We found a higher prevalence in the Western and Central Europe and North America UNAIDS region (where most of the countries are classified as high-income) and lower in all UNAIDS Africa regions (West and Central Africa and Eastern and Southern Africa, where most of the countries are classified as low and lower-middle income). This might be explained by the fact that tobacco is more affordable for populations with higher socioeconomic status. In addition, people have less chance to smoke in warm-weather context where most of low- and lower-middle income countries are located [25]. Furthermore, Western & Central Europe and North America countries might be culturally more prone to smoke. Indeed, in the general population, age-standardized prevalence of tobacco smoking among persons 15 years and older was higher in World Health Organization (WHO) Europe region in 2016 and may partly explain a higher prevalence of active smoking in PLHIV on ART in Western and Central Europe and North America UNAIDS countries [26]. Although, Western and Central Europe and North America UNAIDS region had the highest prevalence of active smoking, the burden in terms of number of active smokers in PLHIV on ART was higher in Eastern and Southern Africa, which accounted for almost one-third of all PLHIV with active smoking. Hence, prioritization of resources and implementation of context-specific public health interventions to curb the burden of active smoking in PLHIV should be designed to address sub-Saharan Africa in particular.

We found that the odd of suboptimal ART adherence was significantly higher in active smokers compared to non-active. Evidence indicate that structural and socioeconomic conditions, as well as psychosocial factors (depression, anxiety), that were associated with smoking behavior are also predictive of suboptimal ART adherence. Prior research indicates that smokers, compared with nonsmokers tended to have multiple and inter-related structural and socioeconomic conditions, as well as psychosocial factors, that were associated with suboptimal ART adherence [27]. Improved understanding of the ways in which these factors co-occur and affect health-related behaviors may improve the efforts to decrease the burden of tobacco smoking when integrating adherence interventions into clinical settings.

Our findings have important policy implications for the management of PLHIV on ART because among the 24.5 million PLHIV ongoing ART worldwide [1], more than 4 million are active smokers and may not achieve HIV viral suppression due to suboptimal ART adherence. Both HIV-itself and tobacco smoking are associated with numerous health concerns including but not limited to cancer, cardiovascular diseases, hypertension, lung diseases, diabetes, tuberculosis, immune system diseases, and chronic obstructive pulmonary disease [7, 28–31]. Therefore, curbing the burden of tobacco smoking in PLHIV on ART is paramount and requires efficient interventions against this modifiable behavior and risk factor since ART initiation do not favor quitting smoking [32]. The high active smoking prevalence found in this study calls all HIV healthcare providers to pay more attention to this dreadful public health concern and to integrate quitting tobacco interventions in already built HIV services. All PLHIV with active smoking should be identified and solutions to quit smoking should be implemented including but not limited to mobile phone-based, internet-based, pharmacotherapy-based, nicotine-based, incentives, counseling, and motivational interview interventions [33–44]. Future research should investigate quitting tobacco smoking in the specific population of PLHIV on ART. Countries should fully implement the six MPOWER guidelines issued by the WHO. To date, only Brazil and Turkey have had the highest level of achievement in terms of this guidelines implementation [5].

The findings of this review should be interpreted considering some limitations. First, we included some studies with moderate to high risk of bias. However, when pooling only studies having a low risk of bias, the overall estimate was close to that of the crude analysis. Second, we found a huge statistical heterogeneity between studies for which we undertook subgroup and meta-regression analyses to identify sources of heterogeneity. This is most common to meta-analyses of prevalence data [45]. However, some characteristics that may further explain heterogeneity were not consistently reported in the original studies such as HIV clinical stage, immunological status, HIV viral load and detailed ART regimen to further explore other sources of heterogeneity. Third, the various geographic regions and countries were variably represented. This may weaken the generalizability of our findings. To take in account this variability, we performed a hierarchical meta-analysis considering the variance from UNAIDS regions and country level of income. Fourth, there was methodological and clinical heterogeneity on the method to measure ART adherence, and ART regimen information was lacked or was not consistently reported across included studies. Therefore, we were not able to statistically quantify this heterogeneity although this was not substantial when measuring the association between active smoking and ART adherence. Finally, there were some missing data concerning study characteristics for which we have undertaken multiple imputation to address this limitation. Despite these limitations, this systematic review and meta-analysis provided a clear summary of the existing knowledge on the global burden of active smoking among PLHIV on ART at global and regional levels and according to the country level of income. It is also the first measuring the association between active smoking and ART adherence.

## Conclusions

This study suggests a high prevalence of active smoking in PLHIV on ART and an association between active smoking and ART suboptimal adherence. As such, active smoking in PLHIV should be prioritized among HIV health care providers, policy makers and stakeholders from the health sector for improved detection, overall proper management and efficient control. This study supports the need for specific strategies to reduce the burden of active smoking in PLHIV, with primer focus on males and on sub-Saharan Africa, the epicenter of HIV pandemic with the highest number of active smokers among PLHIV on ART.

## Supplementary Information

**Additional file 1.** Additional figures and tables.

## Data Availability

All data generated or analyzed during this study are included in this published article and its supplementary information files.

## Abbreviations

ART: Antiretroviral therapy
*CI*: Confidence interval
HIV: Human immunodeficiency virus
*OR*: Odds ratio
PLHIV: People living with HIV
UNAIDS: Joint United Program on HIV/AIDS
WHO: World Health Organization
