# Supplementary material for: Global burden of active smoking among people living with HIV on antiretroviral therapy: a systematic review and meta-analysis
Source: Infect Dis Poverty. 2021 Feb 12;10:12. doi: 10.1186/s40249-021-00799-3 (PMC7881452; doi:10.1186/s40249-021-00799-3)
Supplement: Supplementary file 1 — Additional file 1. Additional figures and tables. [file 40249_2021_799_MOESM1_ESM.pdf]

Global burden of active smoking among people living with HIV on antiretroviral therapy

a systematic review and meta-analysis

APPENDIX

Supplementary Tables

Supplementary Table 1. Search strategy in EMBASE 3

Supplementary Table 2. Characteristics of studies investigating the prevalence of active smoking in people living with HIV undergoing antiretroviral therapy 4

Supplementary Table 3. Individual characteristics of studies investigating the prevalence of active smoking in people living with HIV undergoing antiretroviral therapy 5

Supplementary Table 4. Characteristics of included studies investigating the association between active smoking and non-adherence to antiretroviral therapy 18

Supplementary Table 5. Individual characteristics of included studies investigating the association between active smoking and non-adherence to antiretroviral therapy 19

Supplementary Table 6. Sensitivity analysis of prevalence of active smoking in the global population living with HIV undergoing ART 22

Supplementary Figures

Supplementary Figure 1. PRISMA flow chart 25

Supplementary Figure 2. Funnel plot for meta-analysis of prevalence of active smoking in people undergoing antiretroviral therapy 26

Supplementary Figure 3. Meta-analysis prevalence of active tobacco smoking in people living with HIV undergoing antiretroviral therapy in Asia and Pacific 27

Supplementary Figure 4. Meta-analysis prevalence of active tobacco smoking in people living with HIV undergoing antiretroviral therapy in Eastern and Southern Africa 28
Supplementary Figure 5. Meta-analysis prevalence of active tobacco smoking in people living with HIV undergoing antiretroviral therapy in Eastern Europe and Central Asia 29
Supplementary Figure 6. Meta-analysis prevalence of active tobacco smoking in people living with HIV undergoing antiretroviral therapy in Latin America and the Caribbean 29
Supplementary Figure 7. Meta-analysis prevalence of active tobacco smoking in people living with HIV undergoing antiretroviral therapy in Middle-East and North Africa 30
Supplementary Figure 8. Meta-analysis prevalence of active tobacco smoking in people living with HIV undergoing antiretroviral therapy in West and Central Africa 30
Supplementary Figure 9. Meta-analysis prevalence of active tobacco smoking in people living with HIV undergoing antiretroviral therapy in West and Central Europe and North America 31
Supplementary Figure 10. Funnel plot of the meta-analysis of association between exposure to active smoking and non-adherence to antiretroviral therapy in people living with HIV, crude analysis 32
Supplementary Figure 11. Funnel plot of the meta-analysis of association between exposure to active smoking and non-adherence to antiretroviral therapy in people living with HIV, adjusted analysis 32

Supplementary References

Supplementary References 1. Articles included in the meta-analysis prevalence of active smoking in the global population undergoing antiretroviral therapy 33
Supplementary References 2. Articles included in the meta-of the association between active smoking and adherence to antiretroviral therapy in people living with HIV 56

Supplementary Table 1. Search strategy in EMBASE

	**Search terms**
#1	'human immunodeficiency virus infection':ab,ti OR hiv:ab,ti OR aids:ab,ti OR 'acquired immune deficiency syndrome':ab,ti
#2	'antiretrovirus agent':ab,ti OR 'antiretroviral therapy':ab,ti OR 'highly active antiretroviral therapy':ab,ti OR haart:ab,ti OR art:ab,ti OR 'combined antiretroviral therapy':ab,ti OR 'antiretroviral treatment':ab,ti
#3	#1 AND 2
#4	'tobacco'/exp OR tobacco OR 'smoking'/exp<br>OR smoking OR smok* OR 'cigarette'/exp<br>OR cigarette OR cigar* OR tabacum OR 'nicotine'/exp<br>OR nicotine OR 'nicotiana'/exp OR nicotiana
#5	#3 AND #4

Supplementary Table 2. Characteristics of studies investigating the prevalence of active smoking in people living with HIV undergoing antiretroviral therapy

**Variables**	**N = 290 studies**
**General characteristics**	
**Year of publication, range**	2000-2019
**Design, n (%)**	
- Cross-sectional	125 (43.1)
- Cohort	115 (39.7)
- Case control	28 (9.7)
- Clinical trial	22 (7.6)
**Setting, n (%)**	
- Hospital-based	271 (93.5)
- Population-based	9 (3.0)
- Both	4 (1.4)
- Unclear	6 (2.1)
**Methodological quality**	
**Sampling method, n (%)**	
- Non-probabilistic	249 (85.9)
- Probabilistic	41 (14.1)
**Precision, n (%)**	
- Acceptable	119 (41.0)
- Low	171 (59.0)
**Response rate, n (%)**	
- 80% or more	133 (45.9)
- < 80%	14 (4.8)
- Unclear	143 (49.3)
**Timing of data collection, n (%)**	
- Prospectively	230 (79.3)
- Retrospectively	43 (14.8)
- Both	1 (0.3)
- Unclear	16 (5.5)
**Data collection procedure, n (%)**	
- Identical for all participants	286 (98.6)
- Not identical	1 (0.4)
- Unclear	3 (1.0)

Supplementary Table 3. Individual characteristics of studies investigating the prevalence of active smoking in people living with HIV undergoing antiretroviral therapy

Study	Sample	% on ART	Mean age, years	% of Males	Duration since HIV diagnosis, years	Duration on ART, years	Probabilistic Sampling	Timing	Precision acceptable	Response rate adequate	Identical procedure for data collection	Country
**Aaron, 2012**	183	100	28	0	NR	NR	No	Prospectively	No	Yes	Yes	USA
**Abioye, 2015**	2038	100	38.1	31.8	NR	NR	No	Retrospectively	Yes	No	Yes	Tanzania
**Aboud, 2010**	705	100	NR	72.2	NR	NR	No	Prospectively	Yes	Yes	Yes	UK
**Acevedo, 2002**	90	100	42	NR	NR	3	No	Prospectively	No	No	Yes	USA
**Adefolalu, 2014**	232	100	40	29.7	NR	NR	Yes	Prospectively	Yes	Yes	Yes	South Africa
**Aderemi-Williams, 2017**	248	100	40.4	40.3	NR	NR	No	Prospectively	Yes	Yes	Yes	Nigeria
**Alemu, 2016**	296	90.5	33	36.5	NR	NR	No	Prospectively	No	No	Yes	Ethiopia
**Alemu, 2016**	114	90.5	32	46	NR	NR	No	Prospectively	No	No	Yes	Ethiopia
**Alencherry, 2019**	100	100	55	38	12	11	No	Prospectively	No	Yes	Yes	Uganda
**Alencherry, 2019**	167	100	50	78	15	8.8	No	Prospectively	No	Yes	Yes	USA
**Allavena, 2012**	334	100	38.1	72.5	NR	NR	No	Prospectively	No	Yes	Yes	France
**Allavena, 2012**	2660	100	45.8	72.3	NR	NR	No	Prospectively	No	Yes	Yes	France
**Almeida, 2009**	110	100	37.2	50.9	2.33	NR	Yes	Retrospectively	No	No	Yes	Brazil
**Amberbir, 2019**	820	100	NR	28.2	NR	NR	No	Prospectively	Yes	No	Yes	Malawi
**Anastos, 2005**	573	100	40.3	NR	NR	NR	No	Prospectively	Yes	Yes	Yes	USA
**Anastos, 2005**	184	100	38	NR	NR	NR	No	Prospectively	Yes	Yes	Yes	USA
**Anastos, 2005**	204	100	37	0	NR	NR	No	Prospectively	Yes	Yes	Yes	USA
**Anema, 2011**	457	100	46	74.8	NR	NR	No	Retrospectively	Yes	Yes	Yes	Canada
**Ansemant, 2013**	263	91.6	47.7	71.5	13	NR	No	Prospectively	Yes	No	Yes	France
**Arbune, 2017**	102	95	NR	54	NR	NR	No	Unclear	Yes	No	Yes	Romania
**Ayalew, 2016**	340	100	34.4	39.7	NR	NR	No	Retrospectively	No	Yes	Yes	Ethiopia
**Badie, 2017**	170	100	41	63.5	5.5	NR	No	Prospectively	No	Yes	Yes	Iran
**Bailey, 2014**	100	98	29.5	NR	NR	NR	No	Prospectively	Yes	No	Yes	Ukraine
**Baker, 2012**	43	100	48	97	NR	NR	Yes	Prospectively	No	Yes	Yes	USA
**Baker, 2016**	178	100	33	69.7	1.2	NR	No	Prospectively	Yes	Yes	Yes	Global

**Baker, 2016**	154	100	46	70.1	1.3	NR	No	Prospectively	Yes	Yes	Yes	Global (35 countries)
**Balasundaram, 2014**	130	100	NR	61	NR	NR	No	Prospectively	No	Yes	Yes	India
**Baranoski, 2014**	65	100	49	0	13	NR	No	Retrospectively	No	No	Yes	USA
**Barska, 2017**	121	90.9	40	66	8	NR	No	Prospectively	No	No	Yes	Poland
**Batista, 2014**	1380	100	40.6	64.1	NR	NR	No	Prospectively	Yes	No	Yes	Brazil
**Bednasz, 2016**	90	100	NR	72.2	NR	NR	No	Retrospectively	No	No	Yes	USA
**Begovac, 2015**	254	100	49	76	NR	6	No	Prospectively	No	No	Yes	Croatia, Serbia
**Bekolo, 2014**	114	100	43	17.2	NR	3	No	Prospectively	No	No	Yes	Cameroon
**Berg, 2007**	495	100	40	90	4.8	NR	No	Prospectively	Yes	No	Yes	Germany
**Bergersen, 2004**	219	100	41.6	81	7.15	3.75	No	Prospectively	No	No	Yes	Norway
**Bertisch, 2013**	59	95	NR	85	5	NR	No	Prospectively	No	No	Yes	Switzerland
**Besutti, 2016**	1446	100	48.4	71.2	17.5	10.6	No	Unclear	Yes	No	Yes	Italy
**Bhatta, 2018**	132	100	36.1	47	NR	NR	Yes	Prospectively	Yes	Yes	Yes	Nepal
**Bijker, 2019**	3703	100	46	69	NR	9.8	No	Prospectively	Yes	No	Yes	Asia
**Biraguma, 2018**	698	100	38	32.5	NR	NR	Yes	Prospectively	Yes	Yes	Yes	Rwanda
**Boccara, 2006**	42	100	46.2	40	12.4	4.4	No	Retrospectively	No	No	Yes	France
**Boccara, 2006**	42	100	48.7	40	11.2	4.3	No	Retrospectively	No	No	Yes	France
**Boger, 2012**	107	100	46	76	NR	NR	Yes	Prospectively	No	No	Yes	USA
**Bolland, 2019**	44	100	48.7	100	7.8	4.2	No	Prospectively	No	No	No	New Zealand
**Bonnet, 2004**	964	94.3	41	78	7.6	NR	No	Prospectively	Yes	Yes	Yes	France
**Bonolo, 2013**	306	100	33	65	NR	NR	No	Prospectively	No	No	Yes	Brazil
**Brites-Alves, 2018**	106	100	52.5	61.3	NR	12.5	No	Prospectively	No	No	Yes	Brazil
**Brites-Alves, 2018**	114	100	48.8	72.8	NR	11.1	No	Prospectively	No	No	Yes	Brazil
**Brown, 2017**	197	94	50	80	NR	7	No	Prospectively	No	No	Yes	UK
**Buchacz, 2013**	3166	100	47	79	NR	6.8	No	Prospectively	Yes	No	Yes	USA
**Bucher, 2012**	490	100	NR	61.2	NR	NR	No	Prospectively	Yes	Yes	Yes	Switzerland
**Bultum, 2018**	527	100	NR	40.6	NR	NR	No	Prospectively	Yes	No	Yes	Ethiopia
**Cahn, 2010**	4010	100	41.9	73.9	NR	2.1	Yes	Prospectively	Yes	No	Yes	Argentina; Brazil; Chile; Colombia; Ecuador; Peru; Venezuela

**Camargo, 2019**	112	100	42	66	NR	NR	No	Prospectively	Yes	Yes	Yes	Brazil
**Carballo, 2015**	133	90	51	85	12	11.5	No	Unclear	No	Yes	Yes	Switzerland
**Carr, 2015**	163	100	30	88	0.9	NR	Yes	Prospectively	No	Yes	Yes	South America
**Carr, 2015**	128	100	34	71	2.6	NR	Yes	Prospectively	No	Yes	Yes	Asia
**Carr, 2015**	71	100	40	87	2.2	NR	Yes	Prospectively	No	Yes	Yes	Australia; Europe
**Carr, 2015**	46	100	39	22	3.7	NR	Yes	Prospectively	No	Yes	Yes	South Africa
**Carrieri, 2012**	1154	100	37.7	78	NR	5.9	No	Prospectively	Yes	No	Yes	France
**Castley, 2016**	474	100	45	78.5	NR	NR	No	Prospectively	Yes	Yes	Yes	Australia
**Chireshe, 2019**	600	100	41.8	44	NR	6.5	No	Prospectively	Yes	Yes	Yes	Zimbabwe
**Chițu-Tișu, 2016**	60	100	34.6	58.3	4	4	No	Prospectively	No	Yes	Yes	Romania
**Chițu-Tișu, 2017**	60	100	34.81	61.6	5	4	No	Retrospectively	No	Yes	Yes	Romania
**Cioe, 2017**	185	100	42.2	100	NR	NR	No	Prospectively	No	No	Yes	USA
**Cockerham, 2010**	922	100	43	69	NR	NR	No	Prospectively	Yes	Yes	Yes	USA
**Cole, 2018**	134	100	56	93	15	13	No	Prospectively	No	Yes	Yes	Netherlands; UK
**Colon-Lopez, 2018**	6526	91.3	47.2	64.2	NR	NR	No	Prospectively	Yes	No	Yes	Puerto Rico
**Conley, 2015**	452	100	41	78	NR	2.9	No	Prospectively	Yes	Yes	Yes	USA
**Costiniuk, 2019**	101	97	54	87	NR	NR	No	Prospectively	No	No	Yes	Canada
**Cournil, 2012**	207	100	46.8	32.3	NR	8.8	No	Prospectively	No	Yes	Yes	Senegal
**Crane, 2017**	8567	91	46	85	NR	NR	No	Retrospectively	Yes	No	Yes	USA
**Da Silva, 2009**	243	100	41	59.7	5.8	NR	No	Prospectively	No	Yes	Yes	Brazil
**Daglan, 2013**	40	100	23	42.5	20.85	15.9	Yes	Prospectively	No	No	Yes	Romania
**D'Ascenzo, 2014**	206	100	54	88	3.9	1.4	Yes	Retrospectively	No	No	Unclear	Europe; South Africa; USA
**De Fátima Bonolo, 2008**	295	100	NR	65.8	2	2	No	Prospectively	No	Yes	Yes	Brazil
**Degroote, 2014**	218	92	46	79.4	NR	NR	No	Prospectively	No	Yes	Yes	Belgium
**Dentone, 2018**	158	100	39	88	NR	NR	No	Prospectively	No	No	Yes	Italy
**Depairon, 2001**	168	90.5	39	71.4	NR	NR	No	Prospectively	No	No	Yes	Switzerland
**Deshwal, 2019**	475	100	NR	NR	8.1	NR	No	Prospectively	Yes	Yes	Yes	India
**Dimala, 2016**	100	100	40.2	30	NR	NR	No	Prospectively	No	No	Yes	Cameroon
**Dirajlal-Fargo, 2018**	147	100	45.41	68.2	NR	7.2	No	Prospectively	No	Yes	Yes	USA

**Do T, 2016**	4274	100	40.7	70.7	NR	NR	No	Retrospectively	Yes	No	Yes	Thailand Vietnam Indonesia India Hong Kong SAR Philippines Taiwan Malaysia Japan South Korea Singapore China
**Do T, 2016**	1496	100	44	67.8	NR	NR	No	Retrospectively	Yes	No	Yes	Thailand Vietnam Indonesia India Hong Kong SAR Philippines Taiwan Malaysia Japan South Korea Singapore China
**Donald, 2017**	131	100	NR	0	NR	NR	No	Prospectively	No	No	Yes	South Africa
**Drozd, 2017**	28912	100	NR	81	NR	NR	No	Prospectively	Yes	Yes	Yes	Canada, USA
**Dubé, 2006**	33	100	43	100	NR	NR	Yes	Prospectively	No	No	Yes	USA
**Echeverría, 2014**	174	94.4	46.5	84.1	NR	NR	No	Prospectively	No	Yes	Yes	Spain
**Edward, 2013**	214	100	39.1	79.1	NR	NR	No	Prospectively	No	No	Yes	Nigeria
**Engeland, 2008**	193	100	NR	24	NR	NR	Yes	Retrospectively	No	Yes	Yes	USA
**Erlandson, 2012**	250	100	52	88	14	NR	No	Prospectively	No	Yes	Yes	USA
**Erlandson, 2012**	43	100	51.8	86	16.8	NR	No	Prospectively	No	Yes	Yes	USA
**Erlandson, 2012**	66	100	52.1	74	15.8	NR	No	Prospectively	No	Yes	Yes	USA
**Fabbiani, 2013**	245	93.9	46	75.5	11	8	No	Prospectively	No	Yes	Yes	Italy
**Farhadian, 2018**	108	97.3	55	100	NR	NR	No	Prospectively	No	Yes	Yes	USA
**Florindo, 2007**	220	100	NR	76.8	NR	NR	No	Prospectively	Yes	Yes	Yes	Brazil
**Fontela, 2018**	277	100	53	67	NR	NR	No	Prospectively	No	No	Yes	Spain
**Fontela, 2018**	62	100	66	85	NR	NR	No	Prospectively	No	No	Yes	Spain
**Freitas, 2012**	149	100	44.4	45.4	6.7	5	No	Unclear	No	Yes	Yes	Portugal
**Freitas, 2012**	215	100	47	63	9.2	8	No	Unclear	No	Yes	Yes	Portugal
**Fricke, 2012**	40	100	44	NR	14	9.2	No	Prospectively	No	No	Yes	Germany
**Friis-Møller, 2010**	22625	100	40	74.1	4.8	2.5	No	Prospectively	Yes	Yes	Yes	USA; Argentina; Australia; Europe
**Furuya-Kanamori, 2013**	314	100	48.1	100	NR	NR	Yes	Prospectively	No	Yes	Yes	Australia
**Fuster, 2016**	232	94.7	42.1	68.9	7.15	5.12	Yes	Prospectively	No	No	Yes	Chile
**Gaisa, 2014**	728	93.68	NR	NR	NR	NR	No	Prospectively	Yes	Yes	Yes	USA

**Galán, 2016**	89	100	50.04	76.4	NR	NR	No	Prospectively	No	Yes	Yes	Spain
**Gamarel, 2017**	377	100	46.15	100	13.45	9.85	No	Prospectively	No	Yes	Yes	USA
**Gangcuangco, 2016**	138	100	50.5	88	NR	NR	No	Retrospectively	No	No	Yes	USA
**García-Lázaro, 2007**	205	93.2	41.4	NR	NR	NR	Yes	Prospectively	No	No	Yes	Spain
**George, 2009**	195	100	44.1	82.5	8	NR	Yes	Prospectively	No	No	Yes	USA
**George, 2019**	318	100	39	25	NR	4	Yes	Prospectively	No	No	Yes	South Africa
**Ghadaki, 2016**	247	92	49	75.3	NR	NR	No	Prospectively	No	Yes	Yes	Canada
**Ghehi, 2017**	2056	100	35	21.5	NR	NR	No	Prospectively	Yes	Yes	Yes	Côte d'Ivoire
**Gingo, 2015**	936	100	36.8	NR	NR	0	No	Prospectively	Yes	No	Yes	USA
**Gingo, 2015**	1082	100	44.5	NR	NR	8	No	Prospectively	Yes	No	Yes	USA
**Glass, 2006**	8033	100	NR	NR	NR	NR	No	Prospectively	Yes	Yes	Yes	Switzerland
**Gonçalves, 2009**	120	100	NR	NR	NR	NR	No	Prospectively	No	No	Yes	Brazil
**Grint, 2014**	9535	100	41	73.3	NR	NR	No	Prospectively	Yes	No	Yes	Israel; Argentina ; Europe
**Grome, 2017**	70	100	45	57	NR	6.24	No	Prospectively	No	No	Yes	USA
**Guaraldi, 2012**	133	100	48.77	100	14.7	NR	No	Prospectively	No	No	Yes	Italy
**Guaraldi, 2014**	1446	100	49	71.2	NR	NR	No	Prospectively	Yes	Yes	Yes	Italy
**Guzmán-Fulgencio, 2011**	73	95.9	46	79.5	14	18.4	No	Prospectively	No	Yes	Yes	Spain
**Haberer, 2019**	483	100	31	31	NR	0	No	Prospectively	Yes	No	Yes	Uganda
**Haberer, 2019**	421	100	34	30	NR	0	No	Prospectively	Yes	No	Yes	South Africa
**Haeri, 2009**	151	100	27	0	NR	NR	No	Retrospectively	No	No	Yes	USA
**Haskelberg, 2014**	210	100	38.8	47.6	NR	3.3	Yes	Prospectively	No	Yes	Yes	Argentina, India, Malaysia, South Africa, Thailand
**Hatcher, 2012**	270	100	34	100	NR	NR	No	Prospectively	No	Yes	Yes	Uganda
**Heaps-Woodruff, 2016**	46	100	47	89.5	11.45	NR	No	Prospectively	No	No	Yes	USA
**Helleberg, 2015**	17995	100	NR	71.3	NR	NR	No	Prospectively	Yes	Yes	Yes	Europe; North America
**Helou, 2017**	92	100	53	66	NR	NR	No	Retrospectively	No	No	Yes	USA
**Hidalgo-Tenorio, 2018**	95	93.7	43.7	0	13.6	NR	No	Prospectively	No	No	Yes	Spain
**Hileman, 2012**	30	100	NR	86	0.6	NR	No	Retrospectively	No	No	Yes	USA

**Hileman, 2018**	147	100	45.4	78	12	NR	No	Prospectively	Yes	Yes	Yes	USA
**Hinojosa, 2018**	206	98.5	44	NR	8	NR	Yes	Prospectively	Yes	No	Unclear	Mexico
**Ho, 2012**	74	100	47	NR	7	NR	No	Prospectively	No	No	Yes	USA
**Hoffman, 2016**	122	100	49	95	NR	NR	No	Prospectively	No	No	Yes	USA
**Horizon, 2011**	30	100	49	100	NR	NR	No	Retrospectively	No	Yes	Yes	USA
**Hsu, 2016**	149	100	48.5	92.6	15.2	8.1	No	Prospectively	No	No	Yes	USA
**Hulgan, 2014**	37	100	43	0	NR	NR	Yes	Prospectively	Yes	No	Yes	North America
**Iloeje, 2005**	5787	100	39.4	88	NR	NR	No	Prospectively	Yes	Yes	Yes	USA
**Iloeje, 2005**	1755	100	38.7	80	NR	NR	No	Prospectively	Yes	Yes	Yes	USA
**Iqbal, 2010**	211	100	27.1	0	3	NR	No	Prospectively	No	No	Yes	USA
**Iyer, 2019**	254	100	37	82.6	NR	NR	No	Retrospectively	Yes	No	Yes	USA
**Jackiewicz, 2019**	80	100	40.6	NR	NR	NR	No	Prospectively	No	No	Yes	Poland
**Jacobson, 2008**	379	100	43	74.67	NR	NR	No	Prospectively	No	Yes	Yes	USA
**Jaime, 2006**	223	100	38.9	76.8	NR	NR	Yes	Prospectively	No	Yes	Yes	Brazil
**Jerome, 2017**	771	100	NR	28.4	NR	NR	No	Retrospectively	Yes	No	Yes	Benin
**Johs, 2017**	1015	100	51	81	NR	NR	No	Prospectively	Yes	No	Yes	USA
**Jordan, 2014**	100	100	29.86	100	NR	1.35	No	Prospectively	No	Yes	Yes	Vietnam
**Julnes, 2016**	80	100	50	64	18	NR	No	Prospectively	No	No	Yes	USA
**Julnes, 2016**	34	100	50	62	20	NR	No	Prospectively	No	No	Yes	USA
**Kaio, 2013**	182	100	43.76	62.1	11.49	10.08	No	Prospectively	No	No	Yes	Brazil
**Kakar, 2017**	180	100	NR	96	NR	NR	No	Retrospectively	No	No	Yes	Australia
**Katoto, 2018**	474	95.7	43	28	NR	5	Yes	Prospectively	Yes	No	Yes	DRC
**Kazooba, 2017**	1024	100	NR	35	NR	NR	No	Prospectively	Yes	Yes	Yes	Uganda
**Kerchberger, 2019**	1118	100	48.8	0	NR	10.9	No	Retrospectively	Yes	No	Yes	USA
**Kesselring, 2011**	11459	100	38	77	NR	4.8	No	Prospectively	Yes	No	Yes	Netherlands
**Ketut Agus Somia, 2019**	42	100	30	35.72	2.04	1.54	No	Prospectively	No	Yes	Yes	Indonesia
**Ketut Agus Somia, 2019**	42	100	39.5	35.72	3.29	2.71	No	Prospectively	No	Yes	Yes	Indonesia
**Knobel, 2019**	10897	100	48	71.4	15.8	13.1	No	Unclear	Yes	No	Yes	Spain
**Knudsen, 2014**	54	100	49	NR	11	6	No	Prospectively	No	No	Yes	Denmark

**Knudsen, 2014**	54	100	50	NR	9	6	No	Prospectively	No	No	Yes	Denmark
**Knudsen, 2015**	56	100	53	93	NR	NR	No	Prospectively	No	No	Yes	Denmark
**Knudsen, 2015**	102	94	52	75	12.5	NR	No	Prospectively	No	Yes	Yes	Denmark
**Kobayashi, 2019**	79	95	46	NR	NR	NR	Yes	Prospectively	No	Yes	Yes	USA
**Koethe, 2013**	158	100	45	29.8	NR	NR	No	Prospectively	Yes	Yes	Yes	USA
**Koethe, 2016**	70	100	NR	57.14	NR	NR	No	Prospectively	No	No	Yes	USA
**Kooij, 2016**	594	94.4	52.8	88.7	NR	NR	No	Prospectively	Yes	Yes	Yes	Netherlands
**Krentz, 2005**	124	100	NR	NR	NR	NR	No	Retrospectively	No	Yes	Yes	Canada
**Krishnan, 2011**	3158	100	38	82	NR	NR	No	Prospectively	Yes	No	Yes	Global
**Kristoffersen, 2013**	105	100	47.4	89	12.3	8.9	No	Prospectively	No	No	Yes	Denmark
**Krsak, 2015**	438	90	44.3	68	NR	NR	No	Prospectively	Yes	No	Yes	USA
**Kulkarni, 2016**	49	100	43	100	NR	NR	No	Prospectively	No	No	Yes	USA
**Kwong, 2006**	18603	100	36	82.79	NR	NR	No	Prospectively	Yes	Yes	Yes	Netherlands; USA
**Lake, 2013**	35	100	49	57	NR	NR	No	Prospectively	Yes	Yes	Yes	USA
**Lake, 2015**	82	100	NR	95	NR	NR	No	Prospectively	No	No	Yes	USA
**Lake, 2015**	40	100	NR	95	NR	NR	No	Prospectively	No	No	Yes	USA
**Lake, 2018**	1018	100	53.7	100	12.2	NR	No	Prospectively	Yes	No	Yes	USA
**Leader, 2016**	350	100	50.7	81	NR	NR	No	Prospectively	No	Yes	Yes	USA
**León, 2017**	84	100	42	75	NR	NR	No	Prospectively	No	Yes	Yes	Spain
**Leung, 2014**	199	99	49.3	100	12.05	NR	No	Prospectively	No	No	Yes	Canada
**Levy, 2019**	3368	93	50	78.4	13	NR	No	Prospectively	Yes	No	Yes	USA
**Li J, 2018**	957	100	46	62.5	NR	NR	No	Prospectively, Retrospectively	Yes	Yes	Yes	USA
**Lifson, 2015**	4685	100	NR	68	NR	NR	Yes	Prospectively	Yes	Yes	Yes	Africa; Asia; Europe; Israel; North America; South America; Mexico
**Lima, 2009**	87	100	52	91.9	NR	NR	No	Prospectively	Yes	No	Yes	Brazil
**Liu, 2015**	231	94	49.6	91	12.4	NR	No	Prospectively	No	No	Yes	Canada
**Llop, 2018**	128	100	57	73	21	18.3	No	Prospectively	No	No	Yes	Spain
**Lo, 2010**	78	95	46.5	100	13.5	7	No	Prospectively	No	No	Yes	USA
**Longenecker, 2018**	55	100	49	84	NR	1	No	Prospectively	No	No	Yes	USA

**Looby, 2018**	33	91	47	0	14	NR	No	Retrospectively	No	No	Yes	USA
**Lorenz, 2017**	520	93.2	40.8	100	1	NR	No	Prospectively	Yes	No	Yes	USA
**Ma, 2011**	275	100	46	62.9	NR	NR	Yes	Prospectively	No	No	Yes	USA
**MacDonald, 2018**	915	100	36	70.7	1.2	NR	No	Prospectively	Yes	Yes	Yes	Africa, Asia, Europe, Israel, Australia, Mexico, South America, USA
**Madeddu, 2008**	76	100	38.3	82.9	NR	NR	No	Retrospectively	No	Yes	Yes	italy
**Madeddu, 2013**	111	100	42.3	69.4	NR	NR	No	Prospectively	No	Yes	Yes	italy
**Maggi, 2000**	55	100	36	85.5	5	NR	No	Prospectively	No	Yes	Yes	Italy
**Maggi, 2004**	105	100	38	80.9	8	2.16	No	Prospectively	No	Yes	Yes	Italy
**Mai, 2018**	475	100	38.4	62	NR	NR	No	Prospectively	Yes	Yes	Yes	Vietnam
**Makhubele, 2016**	46	100	27	0	NR	NR	No	Prospectively	No	No	Yes	South Africa
**Makinson, 2011**	52	90	48	80.8	NR	NR	No	Retrospectively	No	No	Yes	France
**Mama, 2018**	291	100	37.4	28.5	NR	NR	Yes	Prospectively	Yes	Yes	Yes	Ethiopia
**Mandas, 2009**	86	100	40.9	54.7	NR	NR	No	Unclear	No	Yes	Yes	Italy
**Marando, 2016**	690	100	45	72.5	11.7	NR	Yes	Prospectively	Yes	Yes	Yes	Italy
**Marcellin, 2017**	955	92	45.6	70	16.2	10.3	No	Prospectively	Yes	Yes	Yes	France
**Martin, 2013**	210	100	21	47.6	NR	NR	No	Prospectively	No	Yes	Yes	India, Malaysia, Thailand, South Africa, Argentina
**Mashinya, 2015**	214	100	44.8	20	NR	3.2	No	Prospectively	No	Yes	Yes	South Africa
**May, 2007**	13100	100	50	100	NR	NR	No	Retrospectively	Yes	Yes	Yes	UK;USA
**Mccombe, 2013**	1320	100	43.8	81	NR	NR	No	Prospectively	Yes	Yes	Yes	Canada
**Melaku, 2019**	120	100	42.3	43.3	NR	NR	No	Prospectively	No	Yes	Yes	Ethiopia
**Melaku, 2019**	120	100	38.4	42.3	NR	NR	No	Prospectively	No	Yes	Yes	Ethiopia
**Mercié, 2002**	422	100	41	72.8	7	3	No	Prospectively	Yes	No	Yes	France
**Miller, 2014**	3570	100	NR	77.2	7.3	4	No	Prospectively	Yes	Yes	Yes	Europe, Americas, Western Pacific
**Misra, 2013**	199	100	43.6	NR	9.5	5.6	No	Unclear	Yes	No	Yes	USA
**Missailidis, 2015**	97	100	51	100	13	NR	Yes	Prospectively	No	No	Yes	Sweden
**Monnig, 2016**	124	92.7	42.8	100	9.9	NR	No	Prospectively	No	No	Yes	USA
**Monteiro, 2011**	53	100	43.4	50.94	NR	4.9	No	Unclear	No	No	Yes	Brazil

**Morell, 2016**	96	100	44.8	19.2	NR	7	No	Prospectively	No	No	Yes	Spain
**Morillo, 2018**	53	100	53.6	90.6	NR	NR	Yes	Prospectively	Yes	No	Yes	Spain
**Morojele, 2017**	130	100	NR	60.8	NR	NR	No	Prospectively	No	Yes	Yes	India
**Muiru, 2018**	105	100	49	46	NR	NR	No	Prospectively	No	Yes	Yes	Uganda
**Muramatsu, 2019**	306	96.1	49	95.1	NR	6.23	No	Retrospectively	No	No	Yes	Japan
**Muronya, 2011**	174	100	40.8	38.5	NR	2.96	Yes	Prospectively	No	Yes	Yes	Malawi
**Mussini, 2013**	4942	100	37	72.6	NR	NR	No	Prospectively	Yes	No	Yes	Italy
**Muyanja, 2016**	250	100	36	32	NR	2.6	No	Prospectively	No	Yes	Yes	Uganda
**Nakamura, 2011**	44	97.8	40	100	NR	5.8	No	Unclear	No	Yes	Yes	Japan
**Ndona, 2012**	49	100	43	42.9	NR	NR	No	Prospectively	No	Yes	Yes	RDC
**Nduka, 2016**	306	100	38.6	34.6	4.2	NR	No	Prospectively	Yes	Yes	Yes	Nigeria
**Neumann, 2010**	309	100	42.1	77.67	NR	NR	No	Prospectively	No	No	Yes	Germany
**Nguyen, 2008**	36	100	53	97	12	NR	Yes	Prospectively	No	Yes	Yes	USA
**Nguyen, 2016**	1050	100	35.6	58.4	6.2	4.4	No	Prospectively	Yes	No	Yes	Vietnam
**Niewoehner, 2015**	328	100	37	36	1.5	NR	Yes	Prospectively	No	Yes	Yes	Africa
**Niewoehner, 2015**	103	100	36	73	0.8	NR	Yes	Prospectively	No	Yes	Yes	Asia
**Niewoehner, 2015**	313	100	31.3	92	1.2	NR	Yes	Prospectively	No	Yes	Yes	Australia; Europe; Israel
**Niewoehner, 2015**	191	100	34	86	0.6	NR	Yes	Prospectively	No	Yes	Yes	Mexico; South America
**Nittayananta, 2014**	99	100	38.5	52	7	NR	No	Prospectively	No	Yes	Yes	Thailand
**Nkuize, 2012**	145	100	44.2	47.59	NR	NR	No	Prospectively	No	Yes	Yes	Belgium
**Nolan, 2017**	202	100	50	65.8	NR	NR	No	Retrospectively	No	No	Yes	USA
**North, 2017**	734	100	34	30	NR	NR	No	Prospectively	Yes	No	Yes	Uganda
**North, 2018**	269	100	52	54	NR	9	No	Prospectively	No	No	Yes	Uganda
**Nouaman, 2018**	795	100	34	46.3	NR	0	No	Prospectively	Yes	No	Yes	Zambia
**Obry-Roguet, 2018**	862	96	52.8	67.9	20.3	16	No	Retrospectively	Yes	No	Yes	France
**O'Halloran, 2019**	202	100	55	75	NR	NR	No	Prospectively	No	No	Yes	USA
**Okafor, 2017**	1902	100	40	69	NR	NR	No	Retrospectively	Yes	No	Yes	USA
**Önen, 2010**	122	91.8	55.8	82.8	10.4	NR	No	Prospectively	No	No	Yes	USA
**Pacek, 2014**	358	100	48.9	61.8	NR	0.6	No	Retrospectively	No	No	Yes	USA
**Palacios, 2006**	95	100	40	82	4.7	NR	No	Prospectively	No	Yes	Yes	Spain

**Palella, 2018**	917	100	NR	100	NR	NR	No	Prospectively	Yes	Yes	Yes	USA
**Parikh, 2015**	150	100	52	88	NR	0.5	Yes	Unclear	Yes	No	Yes	USA
**Park, 2017**	147	100	46	78	11.6	5.5	Yes	Prospectively	Yes	No	Yes	USA
**Parrinello, 2012**	226	99	41.7	NR	NR	NR	No	Prospectively	No	Yes	Yes	USA
**Parrinello, 2012**	148	99	42.8	NR	NR	NR	No	Prospectively	No	Yes	Yes	USA
**Passos, 2012**	73	94.5	43	100	8.5	7.7	No	Prospectively	No	No	Yes	Brazil
**Pastori, 2015**	36	100	42.6	69.4	NR	11.4	No	Unclear	No	Yes	Yes	Italy
**Payam, 2011**	68	91.2	48.5	94.1	NR	NR	No	Retrospectively	No	Yes	Yes	USA
**Petoumenos, 2011**	27136	100	38	77	NR	1.8	No	Prospectively	Yes	No	Yes	Argentina; Australia;Europe; USA.
**Petraglia, 2017**	164	90	45.1	70.1	NR	NR	No	Prospectively	No	Yes	Yes	Russia
**Pinzone, 2019**	72	100	47	80.6	10.2	6	No	Prospectively	Yes	No	Yes	Italy
**Pool, 2019**	819	98.1	52	NR	8	NR	No	Prospectively	Yes	Yes	Yes	Ireland, UK
**Poudel, 2014**	233	100	35.6	52.4	NR	NR	No	Prospectively	No	No	Yes	Nepal
**PrayGod, 2017**	273	100	41.5	34.8	NR	NR	No	Retrospectively	No	No	Yes	Tanzania
**Protopopescu, 2012**	599	100	37.6	77.7	NR	NR	No	Prospectively	Yes	No	Yes	France
**Quirico, 2018**	79	100	64.4	51	16	11.3	No	Prospectively	No	Yes	Yes	Italy
**Quiros-Roldan, 2005**	36	100	NR	75	NR	NR	No	Prospectively	No	Yes	Yes	Italy
**Rabkin, 2015**	175	100	45.4	25.7	4	NR	No	Prospectively	No	No	Yes	South Africa
**Rabkin, 2018**	1826	100	47	38	NR	NR	No	Prospectively	Yes	Yes	Yes	Swaziland
**Rasmussen, 2016**	55	100	49	91	10	NR	No	Retrospectively	Yes	No	Yes	Denmark
**Rasmussen, 2016**	182	100	50	92	10	NR	No	Retrospectively	Yes	No	Yes	Denmark
**Reid, 2017**	225	100	46.5	NR	NR	13	No	Prospectively	No	Yes	Yes	USA
**Reid, 2019**	148	100	50	53	NR	8	No	Prospectively	No	No	Yes	USA
**Rezaei-Soufi, 2014**	50	100	36	71.4	3	1	No	Prospectively	No	No	Yes	Iran
**Rezaei-Soufi, 2014**	100	100	38	81.8	3	1	No	Prospectively	No	No	Yes	Iran
**Rocha-Brischiliari, 2014**	178	100	NR	0	NR	NR	No	Retrospectively	Yes	Yes	Yes	Brazil
**Rollet-Kurhajec, 2015**	308	100	44	72	11	6	Yes	Prospectively	No	Yes	Yes	Canada

**Ronit, 2018**	4576	100	36.8	73.4	NR	NR	Yes	Prospectively	Yes	Yes	Yes	Africa;Asia;Europe;Israel;South America;USA
**Ryom, 2018**	1157	93.7	44	88.3	NR	NR	No	Prospectively	Yes	No	Yes	Australia, Europe, and the USA
**Ryom, 2019**	2467	96	60	77.2	NR	NR	No	Prospectively	Yes	Yes	Yes	Europe; USA; Australia
**Sabin, 2008**	517	98.4	49	92	NR	NR	No	Prospectively	Yes	No	Yes	Europe, the USA, Australia
**Salyer, 2006**	95	100	41.3	83	14	11	No	Unclear	No	Yes	Yes	USA
**Sampériz, 2014**	275	92	49	78.2	11.9	NR	No	Prospectively	No	Yes	Yes	Spain
**Sansores, 2008**	207	100	41.3	90	6.92	5.39	No	Unclear	No	No	Yes	Mexico
**Sarfo, 2019**	250	100	45.7	18.8	8.6	NR	No	Unclear	No	Yes	Yes	Ghana
**Seang, 2018**	57	100	57	100	22	11	No	Prospectively	No	Yes	Yes	USA
**Serrano-Villar, 2014**	132	100	47	78	11	7.5	No	Prospectively	No	No	Yes	Spain
**Shahmanesh, 2016**	8762	100	42	76.28	NR	NR	No	Prospectively	Yes	Yes	Yes	Europe; Israel; Argentina
**Sharma, 2013**	100	100	NR	61	NR	NR	No	Prospectively	No	No	Yes	Nepal
**Sherer, 2014**	2035	100	NR	63	NR	NR	No	Prospectively	Yes	No	Yes	North America (the United States); Latin American (Brazil); Europe (France, Germany, Italy, Russia, Spain, and the United Kingdom); Asia (Australia and Korea); and Africa (Cote d'Ivoire and South Africa)
**Shin, 2019**	598	100	37	0	6	NR	No	Retrospectively	Yes	No	Yes	India
**Shirley, 2013**	200	97	49	84	15.2	NR	No	Prospectively	No	Yes	Yes	USA
**Short, 2014**	131	100	NR	NR	NR	NR	No	Prospectively	No	Yes	Yes	UK
**Shuter, 2008**	64	100	NR	55	NR	NR	No	Prospectively	No	No	Yes	USA
**Siedner, 2016**	105	100	49	49	NR	7	No	Prospectively	No	No	Yes	Uganda
**Siemieniuk, 2011**	1946	100	NR	78.5	NR	NR	No	Prospectively	Yes	No	Yes	Canada
**Smit, 2018**	8791	100	43.8	77.9	NR	4.1	No	Prospectively	Yes	No	Yes	Netherlands
**Socías, 2016**	397	100	41	58.9	NR	NR	Yes	Prospectively	Yes	Yes	Yes	Canada
**Søgaard, 2010**	75	100	49.1	85.3	NR	NR	No	Unclear	No	Yes	Yes	Denmark
**Soliman, 2015**	456	100	37	80	1.4	NR	Yes	Prospectively	Yes	Yes	Yes	North America
**Soliman, 2015**	1428	100	38	92	1.1	NR	Yes	Prospectively	Yes	Yes	Yes	Australia, Europe, Israel
**Soliman, 2015**	1048	100	32	86	0.5	NR	Yes	Prospectively	Yes	Yes	Yes	South America

**Soliman, 2015**	154	100	30	80	0.6	NR	Yes	Prospectively	Yes	Yes	Yes	Asia
**Spinelli, 2019**	156	91.03	NR	92.95	NR	NR	No	Retrospectively	No	Yes	Yes	USA
**Sutton, 2019**	26526	100	49.3	97	NR	NR	No	Retrospectively	Yes	No	Yes	USA
**Sutton, 2019**	53052	100	49.3	97	NR	NR	No	Retrospectively	Yes	No	Yes	USA
**Syed, 2013**	67	100	16.7	NR	NR	NR	No	Unclear	No	Yes	Yes	USA
**Szymanek-Pasternak, 2016**	98	100	41	NR	NR	NR	No	Prospectively	No	No	Yes	Poland
**Tarancon-Diez, 2018**	253	96	29	75	19	NR	No	Prospectively	No	No	Yes	Spain
**Thudium, 2018**	1082	98.6	50.7	84.8	NR	NR	No	Retrospectively	Yes	No	Yes	Denmark
**Tomažič, 2007**	37	100	43.8	100	11.5	5.5	Yes	Prospectively	No	No	Yes	Slovenia
**Tomažič, 2007**	35	100	45.5	100	11	5.1	Yes	Prospectively	No	No	Yes	Slovenia
**Troy, 2016**	208	100	46	67	NR	NR	No	Retrospectively	No	Yes	Yes	USA
**Troy, 2016**	191	100	45	66	NR	NR	No	Retrospectively	No	Yes	Yes	USA
**Troy, 2016**	33	100	53	70	NR	NR	No	Retrospectively	No	Yes	Yes	USA
**Troy, 2016**	193	100	46	68	NR	NR	No	Retrospectively	No	Yes	Yes	USA
**Turčinov, 2011**	130	100	43	79	NR	NR	No	Retrospectively	No	No	Yes	Croatia
**van Zoest, 2016**	499	94.7	52.9	88.6	12.2	NR	No	Prospectively	Yes	No	Yes	Netherlands
**Vernon, 2009**	101	90	42	74	7.2	1.2	No	Prospectively	No	No	Yes	USA
**Volpe, 2013**	211	100	45	73	11	3.1	No	Prospectively	No	Yes	Yes	USA
**Waweru, 2013**	207	100	39.9	47.8	NR	NR	Yes	Prospectively	No	No	Yes	South Africa
**Weldehaweria, 2017**	170	100	39.3	42.4	NR	NR	Yes	Prospectively	Yes	No	Yes	Ethiopia
**Weldehaweria, 2017**	170	100	39.2	42.4	NR	NR	Yes	Prospectively	Yes	No	Yes	Ethiopia
**Willig, 2017**	42	100	45.5	0	NR	NR	No	Prospectively	No	No	Yes	USA
**Winston, 2013**	557	100	44	77	NR	NR	No	Prospectively	Yes	Yes	Yes	UK
**Wójtowicz, 2019**	3495	99	32.8	77	NR	NR	No	Prospectively	Yes	Yes	Yes	Switzerland
**Worm, 2009**	23202	100	38	74	NR	NR	No	Prospectively	Yes	No	Yes	USA; Australia; Europe
**Wu, 2019**	1006	98.5	49.3	93.2	NR	NR	No	Prospectively	Yes	No	Yes	Taiwan
**Yanagisawa, 2012**	520	100	47.6	NR	NR	NR	No	Prospectively	Yes	Yes	Yes	Japan
**Yuan, 2006**	3414	100	39.3	86	NR	NR	No	Retrospectively	Yes	Yes	Yes	USA
**Zachry, 2013**	5530	100	NR	85.79	NR	NR	No	Retrospectively	Yes	No	Yes	USA

**Zannou, 2009**	79	100	38	40.5	NR	1.9	No	Prospectively	Yes	No	Yes	Benin
**Zhang, 2018**	1799	100	42	0	NR	NR	No	Prospectively	Yes	No	Unclear	USA

NR: Not reported

Supplementary Table 4. Characteristics of included studies investigating the association between active smoking and non-adherence to antiretroviral therapy

**Variables**	**N = 20**
**General characteristics**	
**Year of publication, range**	2006-2019
**Design, n (%)**	
- Cross-sectional	15 (75)
- Cohort	5 (25)
**Setting, n (%)**	
- Hospital-based	19 (95)
- Population-based	1 (5)
**Sampling**	
- Non-probabilistic	20 (100)
**Methodological quality**	
**Selection of participants, n (%)**	
- Low	10 (50)
- Moderate	8 (40)
- High	2 (10)
**Comparability of included group, n (%)**	
- Low	15 (75)
- Moderate	2 (10)
- High	3 (15)
**Outcome assessment, n (%)**	
- Low	5 (25)
- Moderate	5 (25)
- High	10 (50)

Supplementary Table 5. Individual characteristics of included studies investigating the association between active smoking and non-adherence to antiretroviral therapy

Author	Year	Design	Sampling	Setting	Period of inclusion	Country	Definition of non-adherence	Sample	Variables for adjustment	Selection	Comparability	Outcome
**Sharma**	2013	Cross sectional	Non-Probabilistic	Hospital-based	2011-2012	Nepal	Missing of ART regimen (< 95%)	100		Mod	Low	Low
**Winhusen**	2018	Cohort	Non-Probabilistic	Hospital-based	NR	USA	HIV medication adherence was measured by self-report as the % of pills taken in the last 30 days; "high adherence" was defned as self-reporting taking ≥ 90% of prescribed ART.	623		Low	High	Low
**O'Connor**	2013	Cohort	Non-Probabilistic	Hospital-based	NR	SMART Countries	Community Programs for Clinical Research on AIDS Antiretroviral Medication Adherence Self-Report Form (form 065)	35695	Study arm, sex, age, ethnicity, mode of transmission, education, residence, calendar year, CD4 count, NRTI, PI, NNRTI in the regimen, No of ART classes experienced, event in past year, history of of AIDS defining illness, concomitant drugs,	Low	Low	Low
**Bonolo [Women]**	2013	Cohort	Non-Probabilistic	Hospital-based	2001-2002	Brazil	Intake of less than 95% of the prescribed number of doses in the last 3 days	100	Marital status, Alcohol, Self-reported difficulty for ART, Adverse events, Time between HIV and first ART, education, race, income, job, disclosure of HIV status, lifetime IDU, lifetime illicit drug use, psychological support, understanding medical counseling and ART prescription, Number of pills, CD4 count baseline, CDC classification of clinical presentation, baseline anxiety and depression, self-perceived quality of life	Low	Low	Mod
**Bonolo [Men]**	2013	Cohort	Non-Probabilistic	Hospital-based	2001-2002	Brazil	Intake of less than 95% of the prescribed number of doses in the last 3 days	195	Marital status, Alcohol, Self-reported difficulty for ART, Adverse events, Time between HIV and first ART, education, race, income, job, disclosure of HIV status, lifetime IDU, lifetime illicit drug use, psychological support, understanding medical counseling and ART prescription, Number of pills, CD4 count baseline, CDC classification of clinical presentation, baseline anxiety and depression, self-perceived quality of life	Low	Low	Mod
**Yuan**	2006	Cohort	Non-Probabilistic	Hospital-based	1996-2003	USA	Non-compliance attributable to patient preference, general non-compliance, including non-compliance on the physician's directive	1341		Low	High	Low
**Jordan**	2014	Cross sectional	Non-Probabilistic	Hospital-based	2005	VietNam	Adherence to currently prescribed ART was assessed using the patient's subjective rating on a one-item Likert scale of how well he was able to take all his medications in the past 30 days (perfect, very good, good, fair, or poor).	528	CD4 count, live alone, drug use last 6 mo, alcohol, duration on ART, ever TB, hepatitis B and C, Bothersome symptoms	Mod	Low	High
**Cioe**	2017	Cross sectional	Non-Probabilistic	Hospital-based	2011-2015	USA	An interviewer asked participants to reflect back on the past 30 days, mark memorable events (e.g., vacations, birthdays, paycheck days, parties) on the calendar as anchor points, and then recall day by day whether or not they had missed any doses of their HIV medications. The authors dichotomized 30-day ART adherence at greater than (perfect/near perfect) or less than (imperfect) 95 % adherence	166	MSM color, income, education, marital status, drug use, cocaine use, methamphetamine use, popper use, marijuana use, age, timing living with HIV, age at smoking initiation, drinking behaviors	High	Low	High

**Nolan**	2017	Cross sectional	Non-Probabilistic	Hospital-based	2012-2014	USA	Self reported past 30-day ART adherence (<90% vs. ≥90%) measured using a validated Visual Analogue Scale (VAS)	200		Low	Low	High
**Batista**	2014	Cross sectional	Non-Probabilistic	Hospital-based	2007-2009	Brazil	Self-reported irregular use of combined antiretroviral therapy (cART), categorized in 'yes' - when patients reported a discontinuation of treatment at some point on their own - and 'no'.	1380	Age, use of crack	Low	Low	High
**Peretti-Watel**	2006	Cross sectional	Non-Probabilistic	Hospital-based	2003	France	Adherence to HAART was assessed with a dichotomous outcome (highly adherent versus non-adherent). Adherence was assessed by four survey questions about dose-taking during the previous 7 days: (1) Did you scrupulously follow prescribed doses of HAART? (2) Did you miss at least one dose during the last week? (3) Did you take the entire daily dose at one time rather than at the prescribed intervals? [This question was eliminated for patients following once-daily regimens.] and (4) Did you fail to follow the prescribed intervals for at least one dose? Patients were defined as highly adherent (versus poorly adherent) if they answered "yes" to the first question and "no" to the second, third, and fourth questions	2484	Age, Living with a partner, Being migrant, financial difficulties in household, transmission group, Number of years since HIV diagnosis, HAART adverse effect, Alcohol abuse, Patients on Drug Maintenance therapy, Multiple addictions	Low	Low	High
**Soares**	2019	Cross sectional	Non-Probabilistic	Hospital-based	2012-2013	Brazil	Nonadherence was defined as intake of less than 90% of the medications prescribed in the last week, considering the number of pills taken by asking the patients and the timetable for each intake	253	Age, Physical activity, Disclosure HIV status with partner, Lack of ART. Others: Alcohol, religious activity, illicit drug use, ART regimens, Partner infected with HIV, Number of casual partners over the last 12 months, Casual partners over the last 12 months, Long-term partners	Mod	Low	High
**Nguyen**	2016	Cross sectional	Non-Probabilistic	Hospital-based	2013	VietNam	Adherence during the last 30 days was measured by the 100-point visual analog scale (VAS), where 0 indicates complete nonadherence and 100 indicates complete adherence. The established cut point of 95 or above was used to indicate adequate adherence.	1050		Mod	Low	High
**Degroote**	2014	Cross sectional	Non-Probabilistic	Hospital-based	2012	Belgium	Simplified Medication Adherence Questionnaire: A patient is considered to be not adherent if one or more of the four first questions were answered affirmatively, if he/she missed more than two doses in the past week or if there were more than 2 days without medication during the past 3 months.	218	Sex, Neurocognitive complaints	Mod	Low	High
**Camargo**	2019	Cross sectional	Non-Probabilistic	Hospital-based	2014-2017	Brazil	Adherence to therapy was evaluated using the Brazilian adaptation of ''Assessment of Adherence to Antiretroviral Therapy Questionnaire'' (CEAT-VIH), which has 20 self-reported questions. The total score for the questionnaire ranges from 17 to 89 points; the higher the score is the better the adherence, that is, low/insufficient adherence (score <70), intermediate adherence (score between 71 and 78), or optimal/adequate adherence (score >79).	112		Mod	Low	High
**Wei**	2015	Cross sectional	Non-Probabilistic	Hospital-based	2014	China	Self-administrated questionnaire was used to collect the data about HAART compliance	276	demographics, ART status, social support and discrimination, WHO staging, ART side effects	Low	Mod	High

**Cropsey**	2016	Cross sectional	Non-Probabilistic	Hospital-based	2005-2008	USA	No missed doses or missed any doses in the past 3 months	2928		Mod	High	Low
**Aye**	2017	Cross sectional	Non-Probabilistic	Hospital-based	2016	Maynnar	The authors used the Adult AIDS Clinical Trials Group (AACTG) adherence instrument and 30 days visual analogue scale (VAS) to assess adherence. AACTG is four days recall method for measuring adherence that also assesses reasons of non-adherence. VAS adherence assessment is the Medication Self-report Inventory that can be used to assess adherence to a single antiretroviral medication. Adherence refers to the PLHIV took ≥95% of doses taken over the past 30 days. On the other hand, patients with <95% of doses taken over the past 30 days, were defined as non-adherence	300	Gender, age, marital status, education, occupation, duration of ART, CD4 count, line of ART, ART regimen, diagnosis of chronic disease, Information, Motivation and Behavioral skills on adherence information, adherence on motivation, adherence on behavioural skills, alcohol, disclosure, stigma, social support, partner who is not on ART, depression, erectile dysfunction	High	Low	Mod
**Silva**	2015	Cross sectional	Non-Probabilistic	Hospital-based	2010	Brazil	This outcome variable was constructed based on the pharmacy's drug dispensation records, according to the dates scheduled for each patient. A seven-day delay per month for the collection of drugs was allowed. For each patient, a binary indicator was constructed with "0" and "1" values to classify adherence status. The "0" value was failure to show up at the pharmacy to collect the drugs prescribed on the scheduled date; the "1" value meant going to the pharmacy on the scheduled date. For each patient, at the completion of a six-month follow-up, an index ranging from 0 to 6 was obtained from the sum of the binary indicator. For analytical purposes, patients were grouped in two categories: "adherence" – for those whose score was ≥ 5, and nonadherence" – for those with score < 5	205	Sex, Age, Employment status, marital status, sexual orientation, alcohol, use of illicit drugs, CD4 count, clinical status at HAART initiation, number of HAART, type of HAART, adverse drugs	Mod	Low	Mod
**Murri**	2009	Cross sectional	Non-Probabilistic	Population-based	2006	Italy	Having discontinued therapy for at least 1 day	296	Age, sex, education, IDU, hepatitis B or C, CD4 count, HIV RNA, PIs, NNRTIs, Number of daily doses, Third or more scheme, number of pills, Missing at least one dose in the previous week, Self-reporting suboptimal adherence (Taking therapy not so well or bad), Uncorrect timing, Believing therapy has ''nothing,'' ''poor'' or ''enough efficacy'', Seek for information on AIDS=HIV, Take homeopathic drugs, Physical Health Summary, Mental Health Summary, Symptom score	Low	Mod	Mod

Supplementary Table 6. Sensitivity analysis of prevalence of active smoking in the global population living with HIV undergoing ART

	Prevalence, %	95% confidence intervals	N studies	N participants	Heterogeneity		Egger test
					I^2, %	P value	
**Global**	**36.1**	**33.7-38.5**	**329**	**462,104**	**99.5**	**< 0.0001**	**0.0002**
- All participants on ART	34.5	31.8-37.2	268	420,575	99.6	< 0.0001	0.0001
- Acceptable precision	33.7	30.1-37.4	132	431,453	99.8	< 0.0001	0.0007
- Probabilistic sampling	31.5	26.0-37.3	52	25,224	98.0	< 0.0001	0.324
- Prospective data collection	35.1	32.5-37.7	261	317,315	99.5	< 0.0001	< 0.0001
- Response rate ≥ 80%	35.4	32.2-38.8	157	193,851	99.5	< 0.0001	< 0.0001
- Identical data collection procedure	36.1	33.7-38.6	325	459,849	99.5	< 0.0001	0.0002

Supplementary Table 7. Complete cases meta-regression analysis of sources of heterogeneity and factors associated with the variation of prevalence of active tobacco smoking in people living with HIV ongoing antiretroviral therapy

	N studies	N participants	Univariable model			Multivariable model		
	N = 329	N = 462,104	Prevalence difference (95% confidence intervals)	*P* value	R^2	Adjusted prevalence difference (95% confidence intervals)	*P* value	LR test
**Country level of income**				< 0.0001	39.4%			0.023
- Low	30	11,329	Ref			Ref		
- Lower-Middle	21	9,858	9.1 (-1.4; 20.7)	0.091		-0.7 (-4.3; 3.0)	0.916	
- Upper-Middle	39	8,252	25.9 (15.4; 37.2)	< 0.0001		10.0 (6.3; 13.9)	0.092	
- High	216	339,022	51.2 (41.1; 62.0)	< 0.0001		13.8 (9.2; 18.6)	0.047	
- Multi-region	23	93,643	26.4 (14.7; 39.4)	< 0.0001		-2.0 (-6.3; 2.5)	0.776	
**UNAIDS**				< 0.0001	45.3%			< 0.0001
- West and Central Europe and North America	203	293,547	Ref			Ref		
- West and Central Africa	12	4,868	-40.9 (-46.5; -34.6)	< 0.0001		-32.0 (-35.1; -28.8)	< 0.0001	
- Eastern and Southern Africa	34	12,676	-33.3 (-37.4; -29.0)	< 0.0001		-26.0 (-28.9; -22.9)	< 0.0001	
- Eastern Europe and Central Asia	2	264	-2.2 (-23.3; 24.6)	0.865		5.5 (-2.6; 14.4)	0.650	
- Latin America and the Caribbean	24	16,385	-16.0 (-21.9; -9.6)	< 0.0001		-13.9 (-17.1; -10.6)	0.020	
- Asia and the Pacific	27	16,352	-12.4 (-18.2; -6.0)	0.0002		-4.9 (-7.6; -2.1)	0.388	
- Middle East and North Africa	3	320	-12.0 (-28.1; 7.6)	0.212		-10.9 (-17.1; -4.3)	0.401	
- Multi-region	24	117,692	-11.5 (-17.7; -4.9)	0.0009		-4.0 (-7.1; -0.9)	0.622	
**% of males**					14.0%			
- Increase of 10%	301	446,434	3.4 (2.4-4.4)	< 0.0001				
**Duration since diagnostic infection**					9.4%			
- By increase of 5 years	119	68,194	12.5 (5.2-20.3)	0.0006				
**Duration of antiretroviral therapy**					0.2%			
- By increase of 5 years	98	117,811	2.7 (-0.9; 15.3)	0.625				
**Mean age**					3.4%			
- By increase of 10 years	290	380,280	6.2 (2.3-10.3)	0.0018				
**Response rate**					0.1%			
- < 80% or not described	172	268,253	Ref					
- ≥ 80%	157	193,851	-1.2 (-6.1; 3.8)	0.628				
**Sampling method**					0.9%			

- Non probabilistic	277	436,880	Ref					
- Probabilistic	52	25,224	-5.5 (-11.8; 1.1)	0.102				
**Timing of data collection and analysis**				0.238	1.3%			
- Prospectively	261	317,315	Ref					
- Retrospectively	50	129,628	4.6 (-2.4; 12.2)	0.205				
- Both	1	957	42.4 (-8.7; 122.2)	0.119				
- Unclear	17	14,204	4.0 (-7.1; 16.5)	0.493				
**Precision**					0.8%			
- Low	197	30,651	Ref					
- Acceptable	132	431,453	-4.1 (-8.9; 0.8)	0.102				

ART: antiretroviral therapy; UNAIDS: Joint United Program on HIV; NA: not applicable; LR: likelihood ratio
*Missing data were imputed

Supplementary Figure 1. PRISMA flow chart

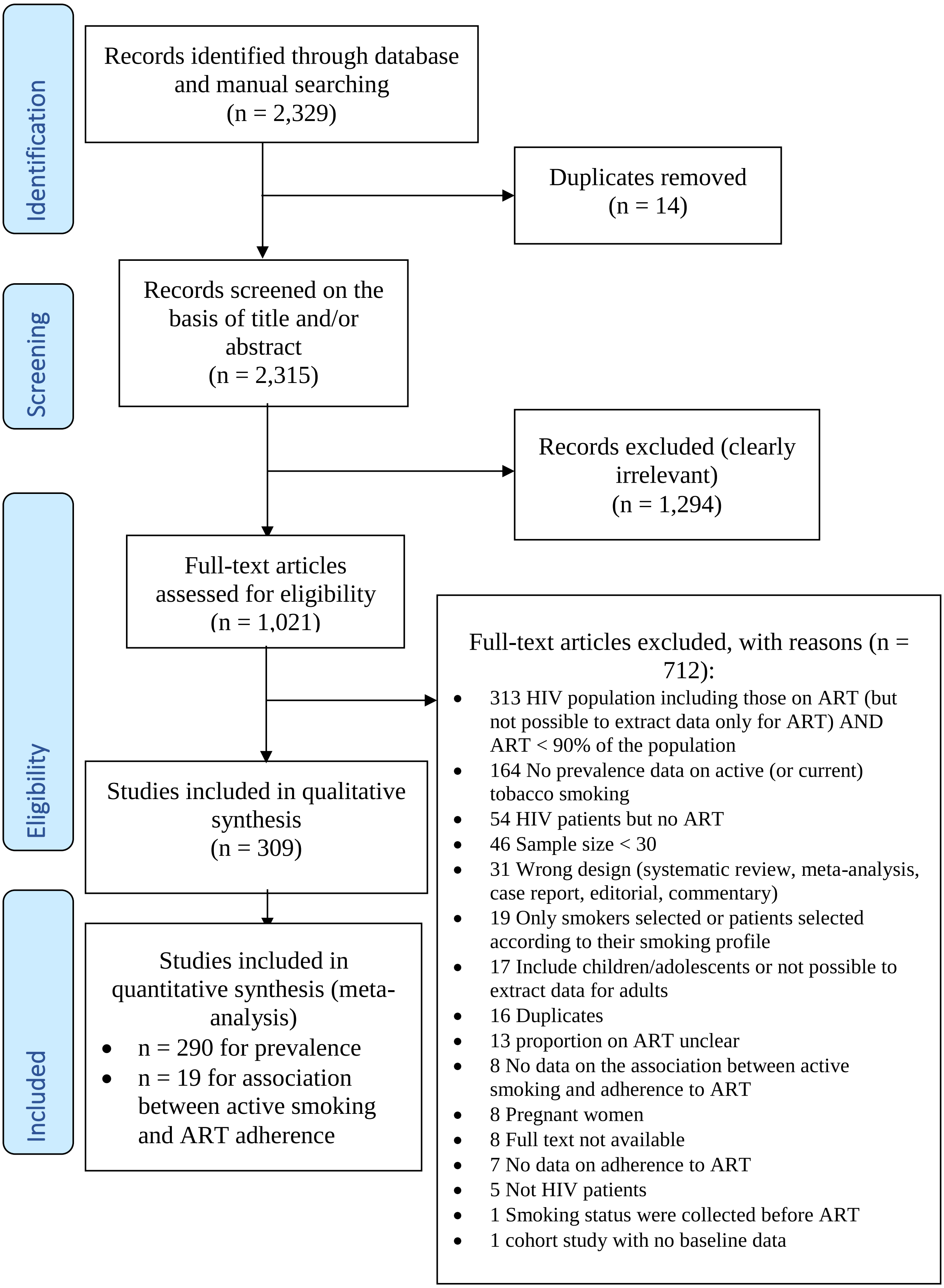

Supplementary Figure 2. Funnel plot for meta-analysis of prevalence of active smoking in people undergoing antiretroviral therapy

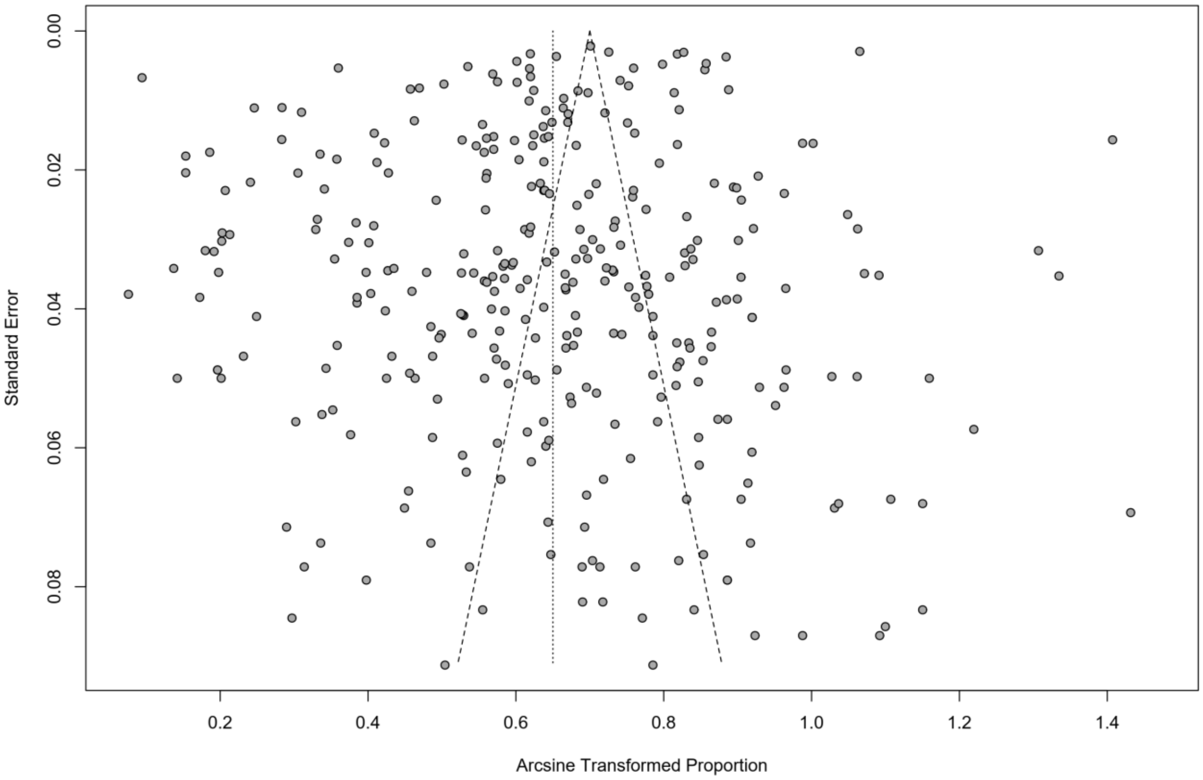

Supplementary Figure 3. Meta-analysis prevalence of active tobacco smoking in people living with HIV undergoing antiretroviral therapy in Asia and Pacific

Author, Year	Cases	Total	Prevalence, %	[95% C.I.]	Weight
Balasundaram, 2014	50	130	38.5	[30.1; 47.4]	3.7%
Bhatta, 2018	35	132	26.5	[19.2; 34.9]	3.7%
Bijker, 2019	758	3703	20.5	[19.2; 21.8]	3.9%
Bolland, 2019	16	44	36.4	[22.4; 52.2]	3.3%
Carr, 2015	29	128	22.7	[15.7; 30.9]	3.7%
Castley, 2016	168	474	35.4	[31.1; 39.9]	3.9%
Deshwal, 2019	169	475	35.6	[31.3; 40.1]	3.9%
Do T, 2016	992	4274	23.2	[22.0; 24.5]	3.9%
Do T, 2016	298	1496	19.9	[17.9; 22.0]	3.9%
Furuya-Kanamori, 2013	106	314	33.8	[28.5; 39.3]	3.8%
Jordan, 2014	84	100	84.0	[75.3; 90.6]	3.6%
Kakar, 2017	69	180	38.3	[31.2; 45.9]	3.7%
Ketut Agus Somia, 2019	4	42	9.5	[2.7; 22.6]	3.3%
Ketut Agus Somia, 2019	11	42	26.2	[13.9; 42.0]	3.3%
Mai, 2018	225	475	47.4	[42.8; 52.0]	3.9%
Morojele, 2017	50	130	38.5	[30.1; 47.4]	3.7%
Muramatsu, 2019	123	306	40.2	[34.7; 45.9]	3.8%
Nakamura, 2011	25	44	56.8	[41.0; 71.7]	3.3%
Nguyen, 2016	373	1050	35.5	[32.6; 38.5]	3.9%
Niewoehner, 2015	20	103	19.4	[12.3; 28.4]	3.6%
Nittayananta, 2014	34	99	34.3	[25.1; 44.6]	3.6%
Poudel, 2014	96	233	41.2	[34.8; 47.8]	3.8%
Sharma, 2013	20	100	20.0	[12.7; 29.2]	3.6%
Shin, 2019	54	598	9.0	[6.9; 11.6]	3.9%
Soliman, 2015	26	154	16.9	[11.3; 23.8]	3.7%
Wu, 2019	319	1006	31.7	[28.8; 34.7]	3.9%
Yanagisawa, 2012	303	520	58.3	[53.9; 62.5]	3.9%
**Pooled Prevalence**		**16352**	**32.6**	**[26.6; 38.9]**	**100.0%**

Heterogeneity: $I^2 = 97.3\%$, $p < 0.0001$

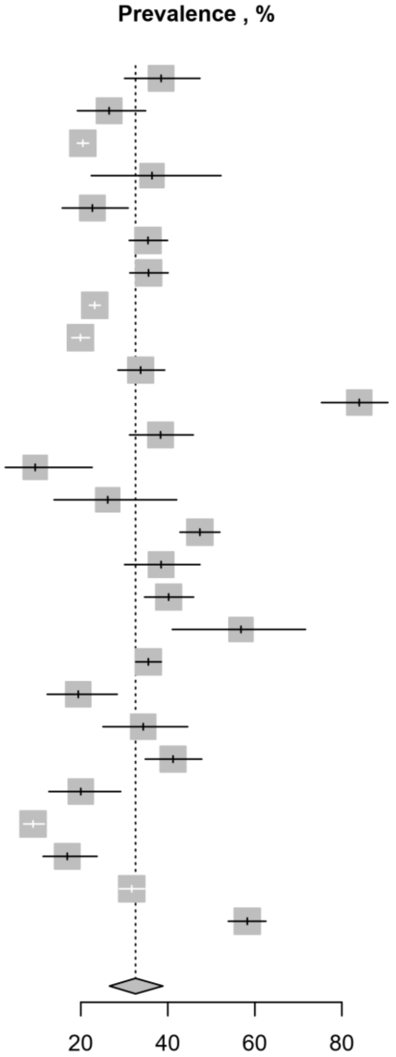

Supplementary Figure 4. Meta-analysis prevalence of active tobacco smoking in people living with HIV undergoing antiretroviral therapy in Eastern and Southern Africa

Author, Year	Cases	Total	Prevalence, %	[95% C.I.]	Weight
Abioye, 2015	121	2038	5.9	[5.0; 7.1]	3.1%
Adefolalu, 2014	28	232	12.1	[8.2; 17.0]	3.0%
Alemu, 2016	12	296	4.1	[2.1; 7.0]	3.0%
Alemu, 2016	25	114	21.9	[14.7; 30.6]	2.8%
Alencherry, 2019	4	100	4.0	[1.1; 9.9]	2.8%
Amberbir, 2019	28	820	3.4	[2.3; 4.9]	3.1%
Ayalew, 2016	36	340	10.6	[7.5; 14.4]	3.0%
Biraguma, 2018	112	698	16.0	[13.4; 19.0]	3.1%
Bultum, 2018	30	527	5.7	[3.9; 8.0]	3.1%
Carr, 2015	5	46	10.9	[3.6; 23.6]	2.5%
Chireshe, 2019	14	600	2.3	[1.3; 3.9]	3.1%
Donald, 2017	30	131	22.9	[16.0; 31.1]	2.9%
George, 2019	50	318	15.7	[11.9; 20.2]	3.0%
Haberer, 2019	54	483	11.2	[8.5; 14.3]	3.1%
Haberer, 2019	94	421	22.3	[18.4; 26.6]	3.0%
Hatcher, 2012	36	270	13.3	[9.5; 18.0]	3.0%
Kazooba, 2017	80	1024	7.8	[6.2; 9.6]	3.1%
Makhubele, 2016	10	46	21.7	[10.9; 36.4]	2.5%
Mama, 2018	13	291	4.5	[2.4; 7.5]	3.0%
Mashinya, 2015	38	214	17.8	[12.9; 23.5]	3.0%
Melaku, 2019	35	120	29.2	[21.2; 38.2]	2.8%
Melaku, 2019	66	120	55.0	[45.7; 64.1]	2.8%
Muiru, 2018	4	105	3.8	[1.0; 9.5]	2.8%
Muronya, 2011	1	174	0.6	[0.0; 3.2]	2.9%
Muyanja, 2016	8	250	3.2	[1.4; 6.2]	3.0%
North, 2017	90	734	12.3	[10.0; 14.9]	3.1%
North, 2018	41	269	15.2	[11.2; 20.1]	3.0%
Nouaman, 2018	86	795	10.8	[8.7; 13.2]	3.1%
PrayGod, 2017	11	273	4.0	[2.0; 7.1]	3.0%
Rabkin, 2015	27	175	15.4	[10.4; 21.6]	2.9%
Siedner, 2016	4	105	3.8	[1.0; 9.5]	2.8%
Waweru, 2013	31	207	15.0	[10.4; 20.6]	3.0%
Weldehaweria, 2017	24	170	14.1	[9.3; 20.3]	2.9%
Weldehaweria, 2017	5	170	2.9	[1.0; 6.7]	2.9%
**Pooled Prevalence**		**12676**	**10.7**	**[7.8; 14.0]**	**100.0%**

Heterogeneity: $I^2 = 94.8\%$, $p < 0.0001$

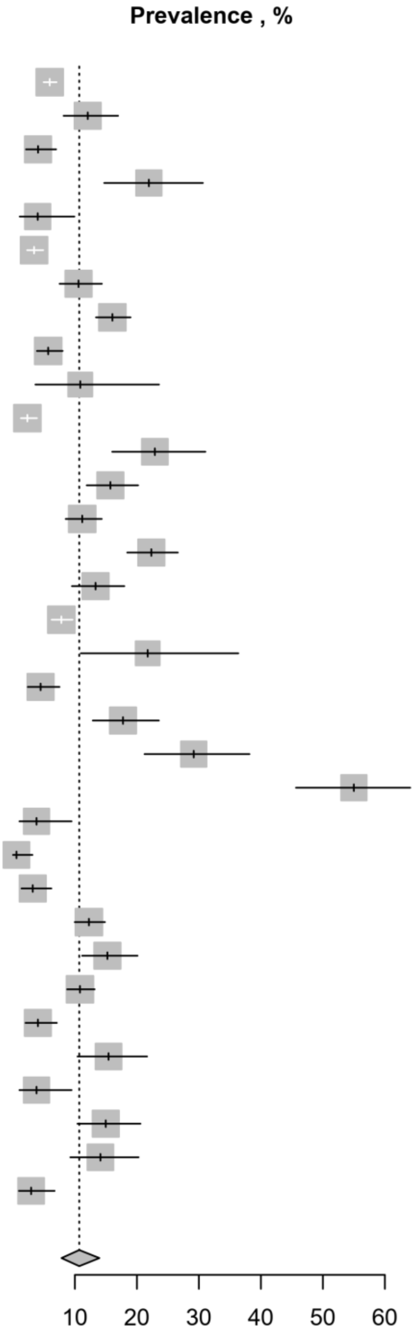

Supplementary Figure 5. Meta-analysis prevalence of active tobacco smoking in people living with HIV undergoing antiretroviral therapy in Eastern Europe and Central Asia

Author, Year	Cases	Total	Prevalence, %	[95% C.I.]	Weight
Bailey, 2014	28	100	28.0	[19.5; 37.9]	49.5%
Petraglia, 2017	96	164	58.5	[50.6; 66.2]	50.5%
**Pooled Prevalence**		**264**	**43.1**	**[15.8; 72.9]**	**100.0%**

Heterogeneity: I^2 = 95.9%, p < 0.0001

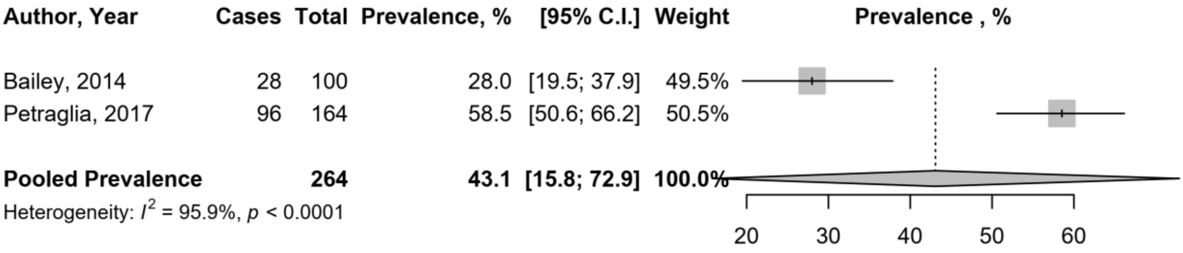

Supplementary Figure 6. Meta-analysis prevalence of active tobacco smoking in people living with HIV undergoing antiretroviral therapy in Latin America and the Caribbean

Author, Year	Cases	Total	Prevalence, %	[95% C.I.]	Weight
Almeida, 2009	59	110	53.6	[43.9; 63.2]	3.9%
Batista, 2014	383	1380	27.8	[25.4; 30.2]	4.7%
Bonolo, 2013	101	306	33.0	[27.8; 38.6]	4.4%
Brites-Alves, 2018	12	106	11.3	[6.0; 18.9]	3.9%
Brites-Alves, 2018	20	114	17.5	[11.1; 25.8]	3.9%
Cahn, 2010	1873	4010	46.7	[45.2; 48.3]	4.7%
Camargo, 2019	33	112	29.5	[21.2; 38.8]	3.9%
Carr, 2015	23	163	14.1	[9.2; 20.4]	4.1%
Colon-Lopez, 2018	1893	6526	29.0	[27.9; 30.1]	4.7%
Da Silva, 2009	62	243	25.5	[20.2; 31.5]	4.3%
De Fátima Bonolo, 2008	99	295	33.6	[28.2; 39.3]	4.4%
Florindo, 2007	69	220	31.4	[25.3; 37.9]	4.3%
Fuster, 2016	92	232	39.7	[33.3; 46.3]	4.3%
Gonçalves, 2009	46	120	38.3	[29.6; 47.6]	3.9%
Hinojosa, 2018	55	206	26.7	[20.8; 33.3]	4.2%
Jaime, 2006	68	223	30.5	[24.5; 37.0]	4.3%
Kaio, 2013	59	182	32.4	[25.7; 39.7]	4.2%
Lima, 2009	34	87	39.1	[28.8; 50.1]	3.7%
Monteiro, 2011	10	53	18.9	[9.4; 32.0]	3.3%
Niewoehner, 2015	54	191	28.3	[22.0; 35.2]	4.2%
Passos, 2012	16	73	21.9	[13.1; 33.1]	3.6%
Rocha-Brischiliari, 2014	35	178	19.7	[14.1; 26.3]	4.2%
Sansores, 2008	44	207	21.3	[15.9; 27.5]	4.3%
Soliman, 2015	296	1048	28.2	[25.5; 31.1]	4.6%
**Pooled Prevalence**		**16385**	**28.8**	**[25.0; 32.8]**	**100.0%**

Heterogeneity: I^2 = 95.9%, p < 0.0001

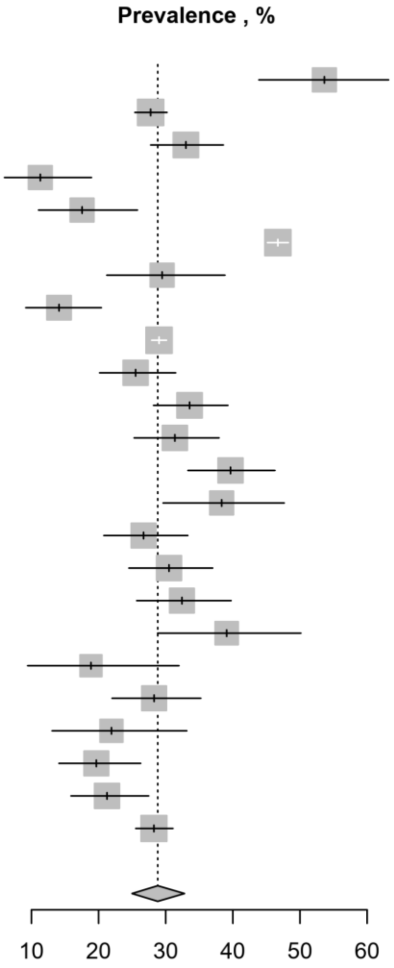

Supplementary Figure 7. Meta-analysis prevalence of active tobacco smoking in people living with HIV undergoing antiretroviral therapy in Middle-East and North Africa

Author, Year	Cases	Total	Prevalence, %	[95% C.I.]	Weight
Badie, 2017	81	170	47.6	[39.9; 55.4]	35.0%
Rezaei-Soufi, 2014	18	50	36.0	[22.9; 50.8]	31.2%
Rezaei-Soufi, 2014	17	100	17.0	[10.2; 25.8]	33.8%
**Pooled Prevalence**		**320**	**32.9**	**[16.1; 52.4]**	**100.0%**

Heterogeneity: $I^2 = 93.0\%$, $p < 0.0001$

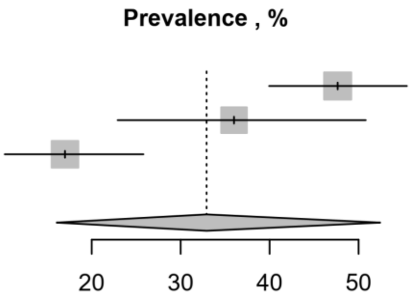

Supplementary Figure 8. Meta-analysis prevalence of active tobacco smoking in people living with HIV undergoing antiretroviral therapy in West and Central Africa

Author, Year	Cases	Total	Prevalence, %	[95% C.I.]	Weight
Aderemi-Williams, 2017	9	248	3.6	[1.7; 6.8]	8.9%
Bekolo, 2014	6	114	5.3	[2.0; 11.1]	7.2%
Cournil, 2012	8	207	3.9	[1.7; 7.5]	8.5%
Dimala, 2016	2	100	2.0	[0.2; 7.0]	6.9%
Edward, 2013	4	214	1.9	[0.5; 4.7]	8.6%
Ghehi, 2017	161	2056	7.8	[6.7; 9.1]	10.7%
Jerome, 2017	18	771	2.3	[1.4; 3.7]	10.2%
Katoto, 2018	20	474	4.2	[2.6; 6.4]	9.8%
Ndona, 2012	4	49	8.2	[2.3; 19.6]	4.9%
Nduka, 2016	32	306	10.5	[7.3; 14.4]	9.2%
Sarfo, 2019	3	250	1.2	[0.2; 3.5]	8.9%
Zannou, 2009	7	79	8.9	[3.6; 17.4]	6.2%
**Pooled Prevalence**		**4868**	**4.4**	**[2.9; 6.3]**	**100.0%**

Heterogeneity: $I^2 = 87.0\%$, $p < 0.0001$

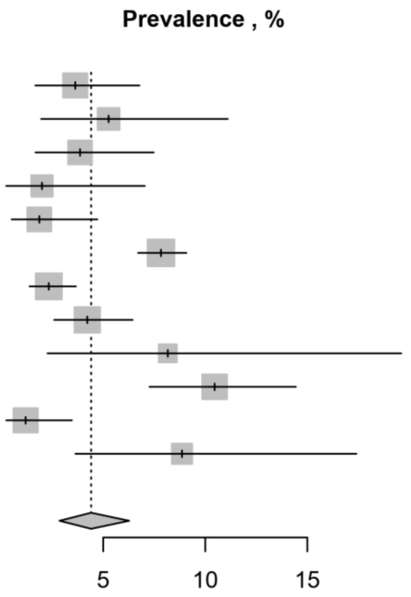

Supplementary Figure 9. Meta-analysis prevalence of active tobacco smoking in people living with HIV undergoing antiretroviral therapy in West and Central Europe and North America

Author, Year	Cases	Total	Prevalence, %	[95% C.I.]	Weight
Aaron, 2012	70	183	38.3	[31.2; 45.7]	0.5%
Aboud, 2010	250	705	35.5	[31.9; 39.1]	0.5%
Acevedo, 2002	46	90	51.1	[40.3; 61.8]	0.5%
Alencherry, 2019	100	167	59.9	[52.0; 67.4]	0.5%
Allavena, 2012	150	334	44.9	[39.5; 50.4]	0.5%
Allavena, 2012	1012	2660	38.0	[36.2; 39.9]	0.5%
Anastos, 2005	367	573	64.0	[60.0; 68.0]	0.5%
Anastos, 2005	86	184	46.7	[39.4; 54.2]	0.5%
Anastos, 2005	78	204	38.2	[31.5; 45.3]	0.5%
Anema, 2011	308	457	67.4	[62.9; 71.7]	0.5%
Ansemant, 2013	120	263	45.6	[39.5; 51.9]	0.5%
Arbune, 2017	51	102	50.0	[39.9; 60.1]	0.5%
Baker, 2012	23	43	53.5	[37.7; 68.8]	0.4%
Baranoski, 2014	22	65	33.8	[22.6; 46.6]	0.5%
Barska, 2017	70	121	57.9	[48.5; 66.8]	0.5%
Bednasz, 2016	35	90	38.9	[28.8; 49.7]	0.5%
Berg, 2007	301	495	60.8	[56.4; 65.1]	0.5%
Bergersen, 2004	119	219	54.3	[47.5; 61.1]	0.5%
Bertisch, 2013	37	59	62.7	[49.1; 75.0]	0.5%
Besutti, 2016	558	1446	38.6	[36.1; 41.2]	0.5%
Boccara, 2006	17	42	40.5	[25.6; 56.7]	0.4%
Boccara, 2006	18	42	42.9	[27.7; 59.0]	0.4%
Boger, 2012	57	107	53.3	[43.4; 63.0]	0.5%
Bonnet, 2004	162	964	16.8	[14.5; 19.3]	0.5%
Brown, 2017	60	197	30.5	[24.1; 37.4]	0.5%
Buchacz, 2013	1674	3166	52.9	[51.1; 54.6]	0.5%
Bucher, 2012	300	490	61.2	[56.8; 65.6]	0.5%
Carballo, 2015	77	133	57.9	[49.0; 66.4]	0.5%
Carrieri, 2012	182	1154	15.8	[13.7; 18.0]	0.5%
Chițu-Tișu, 2017	26	60	43.3	[30.6; 56.8]	0.5%
Chiţu-Tişu, 2016	18	60	30.0	[18.8; 43.2]	0.5%
Cioe, 2017	91	185	49.2	[41.8; 56.6]	0.5%
Cockerham, 2010	366	922	39.7	[36.5; 42.9]	0.5%
Cole, 2018	40	134	29.9	[22.3; 38.4]	0.5%
Conley, 2015	187	452	41.4	[36.8; 46.1]	0.5%
Costiniuk, 2019	74	101	73.3	[63.5; 81.6]	0.5%
Crane, 2017	2880	8567	33.6	[32.6; 34.6]	0.5%
Daglan, 2013	24	40	60.0	[43.3; 75.1]	0.4%
Degroote, 2014	66	218	30.3	[24.3; 36.8]	0.5%
Dentone, 2018	56	158	35.4	[28.0; 43.4]	0.5%
Depairon, 2001	103	168	61.3	[53.5; 68.7]	0.5%
Dirajlal-Fargo, 2018	93	147	63.3	[54.9; 71.1]	0.5%
Drozd, 2017	22130	28912	76.5	[76.0; 77.0]	0.5%
Dubé, 2006	21	33	63.6	[45.1; 79.6]	0.4%
Echeverría, 2014	86	174	49.4	[41.8; 57.1]	0.5%
Engeland, 2008	54	193	28.0	[21.8; 34.9]	0.5%
Erlandson, 2012	74	250	29.6	[24.0; 35.7]	0.5%
Erlandson, 2012	18	43	41.9	[27.0; 57.9]	0.4%
Erlandson, 2012	31	66	47.0	[34.6; 59.7]	0.5%
Fabbiani, 2013	133	245	54.3	[47.8; 60.6]	0.5%
Farhadian, 2018	33	108	30.6	[22.1; 40.2]	0.5%
Fontela, 2018	116	277	41.9	[36.0; 47.9]	0.5%
Fontela, 2018	16	62	25.8	[15.5; 38.5]	0.5%
Freitas, 2012	59	149	39.6	[31.7; 47.9]	0.5%
Freitas, 2012	94	215	43.7	[37.0; 50.6]	0.5%
Fricke, 2012	24	40	60.0	[43.3; 75.1]	0.4%
Gaisa, 2014	235	728	32.3	[28.9; 35.8]	0.5%
Galán, 2016	20	89	22.5	[14.3; 32.6]	0.5%
Gamarel, 2017	106	377	28.1	[23.6; 32.9]	0.5%
Gangcuangco, 2016	30	138	21.7	[15.2; 29.6]	0.5%
García-Lázaro, 2007	158	205	77.1	[70.7; 82.6]	0.5%
George, 2009	65	195	33.3	[26.8; 40.4]	0.5%
Ghadaki, 2016	91	247	36.8	[30.8; 43.2]	0.5%
Gingo, 2015	499	936	53.3	[50.1; 56.5]	0.5%
Gingo, 2015	390	1082	36.0	[33.2; 39.0]	0.5%
Glass, 2006	4579	8033	57.0	[55.9; 58.1]	0.5%
Grome, 2017	25	70	35.7	[24.6; 48.1]	0.5%
Guaraldi, 2012	53	133	39.8	[31.5; 48.7]	0.5%
Guaraldi, 2014	528	1446	36.5	[34.0; 39.1]	0.5%
Guzmán-Fulgencio, 2011	41	73	56.2	[44.1; 67.8]	0.5%
Haeri, 2009	38	151	25.2	[18.5; 32.9]	0.5%
Heaps-Woodruff, 2016	29	46	63.0	[47.5; 76.8]	0.5%
Helleberg, 2015	10767	17995	59.8	[59.1; 60.6]	0.5%
Helou, 2017	39	92	42.4	[32.1; 53.1]	0.5%
Hidalgo-Tenorio, 2018	61	95	64.2	[53.7; 73.8]	0.5%
Hileman, 2012	15	30	50.0	[31.3; 68.7]	0.4%
Hileman, 2018	93	147	63.3	[54.9; 71.1]	0.5%
Ho, 2012	10	74	13.5	[6.7; 23.5]	0.5%
Hoffman, 2016	15	122	12.3	[7.0; 19.5]	0.5%
Horizon, 2011	7	30	23.3	[9.9; 42.3]	0.4%
Hsu, 2016	38	149	25.5	[18.7; 33.3]	0.5%
Hulgan, 2014	16	37	43.2	[27.1; 60.5]	0.4%
Iloeje, 2005	1953	5787	33.7	[32.5; 35.0]	0.5%
Iloeje, 2005	678	1755	38.6	[36.3; 41.0]	0.5%
Iqbal, 2010	94	211	44.5	[37.7; 51.5]	0.5%
Iyer, 2019	140	254	55.1	[48.8; 61.3]	0.5%
Jackiewicz, 2019	47	80	58.8	[47.2; 69.6]	0.5%
Jacobson, 2008	186	379	49.1	[43.9; 54.2]	0.5%
Johs, 2017	257	1015	25.3	[22.7; 28.1]	0.5%
Julnes, 2016	48	80	60.0	[48.4; 70.8]	0.5%
Julnes, 2016	27	34	79.4	[62.1; 91.3]	0.4%
Kerchberger, 2019	382	1118	34.2	[31.4; 37.0]	0.5%
Kesselring, 2011	6552	11459	57.2	[56.3; 58.1]	0.5%
Knobel, 2019	5590	10897	51.3	[50.4; 52.2]	0.5%
Knudsen, 2014	45	54	83.3	[70.7; 92.1]	0.5%
Knudsen, 2014	40	54	74.1	[60.3; 85.0]	0.5%
Knudsen, 2015	34	102	33.3	[24.3; 43.4]	0.5%
Knudsen, 2015	23	56	41.1	[28.1; 55.0]	0.5%
Kobayashi, 2019	28	79	35.4	[25.0; 47.0]	0.5%
Koethe, 2013	76	158	48.1	[40.1; 56.2]	0.5%
Koethe, 2016	25	70	35.7	[24.6; 48.1]	0.5%
Kooij, 2016	168	594	28.3	[24.7; 32.1]	0.5%
Krentz, 2005	68	124	54.8	[45.7; 63.8]	0.5%
Kristoffersen, 2013	39	105	37.1	[27.9; 47.1]	0.5%
Krsak, 2015	207	438	47.3	[42.5; 52.1]	0.5%
Kulkarni, 2016	20	49	40.8	[27.0; 55.8]	0.5%
Kwong, 2006	6898	18603	37.1	[36.4; 37.8]	0.5%
Lake, 2013	3	35	8.6	[1.8; 23.1]	0.4%
Lake, 2015	9	82	11.0	[5.1; 19.8]	0.5%
Lake, 2015	6	40	15.0	[5.7; 29.8]	0.4%
Lake, 2018	991	1018	97.3	[96.2; 98.2]	0.5%
Leader, 2016	191	350	54.6	[49.2; 59.9]	0.5%
León, 2017	10	84	11.9	[5.9; 20.8]	0.5%
Leung, 2014	104	199	52.3	[45.1; 59.4]	0.5%
Levy, 2019	1345	3368	39.9	[38.3; 41.6]	0.5%
Li J, 2018	667	957	69.7	[66.7; 72.6]	0.5%
Liu, 2015	128	231	55.4	[48.8; 61.9]	0.5%
Llop, 2018	44	128	34.4	[26.2; 43.3]	0.5%
Lo, 2010	35	78	44.9	[33.6; 56.6]	0.5%
Longenecker, 2018	30	55	54.5	[40.6; 68.0]	0.5%
Looby, 2018	23	33	69.7	[51.3; 84.4]	0.4%
Lorenz, 2017	182	520	35.0	[30.9; 39.3]	0.5%
Ma, 2011	154	275	56.0	[49.9; 62.0]	0.5%
Madeddu, 2008	67	76	88.2	[78.7; 94.4]	0.5%
Madeddu, 2013	63	111	56.8	[47.0; 66.1]	0.5%
Maggi, 2000	34	55	61.8	[47.7; 74.6]	0.5%
Maggi, 2004	71	105	67.6	[57.8; 76.4]	0.5%
Makinson, 2011	51	52	98.1	[89.7; 100.0]	0.5%
Mandas, 2009	57	86	66.3	[55.3; 76.1]	0.5%
Marando, 2016	351	690	50.9	[47.1; 54.7]	0.5%
Marcellin, 2017	678	955	71.0	[68.0; 73.9]	0.5%
May, 2007	4192	13100	32.0	[31.2; 32.8]	0.5%
Mccombe, 2013	467	1320	35.4	[32.8; 38.0]	0.5%
Mercié, 2002	261	422	61.8	[57.0; 66.5]	0.5%
Misra, 2013	123	199	61.8	[54.7; 68.6]	0.5%
Missailidis, 2015	30	97	30.9	[21.9; 41.1]	0.5%
Monnig, 2016	66	124	53.2	[44.1; 62.2]	0.5%
Morell, 2016	51	96	53.1	[42.7; 63.4]	0.5%
Morillo, 2018	39	53	73.6	[59.7; 84.7]	0.5%
Mussini, 2013	2252	4942	45.6	[44.2; 47.0]	0.5%
Neumann, 429	196	309	63.4	[57.8; 68.8]	0.5%
Nguyen, 2008	10	36	27.8	[14.2; 45.2]	0.4%
Nkuize, 2012	48	145	33.1	[25.5; 41.4]	0.5%
Nolan, 2017	159	202	78.7	[72.4; 84.1]	0.5%
O'Halloran, 2019	99	202	49.0	[41.9; 56.1]	0.5%
Obry-Roguet, 2018	251	862	29.1	[26.1; 32.3]	0.5%
Okafor, 2017	679	1902	35.7	[33.5; 37.9]	0.5%
Önen, 2010	48	122	39.3	[30.6; 48.6]	0.5%
Pacek, 2014	269	358	75.1	[70.3; 79.5]	0.5%
Palacios, 2006	64	95	67.4	[57.0; 76.6]	0.5%
Palella, 2018	312	917	34.0	[31.0; 37.2]	0.5%
Parikh, 2015	38	150	25.3	[18.6; 33.1]	0.5%
Park, 2017	93	147	63.3	[54.9; 71.1]	0.5%
Parrinello, 2012	81	226	35.8	[29.6; 42.5]	0.5%
Parrinello, 2012	74	148	50.0	[41.7; 58.3]	0.5%
Pastori, 2015	20	36	55.6	[38.1; 72.1]	0.4%
Payam, 2011	43	68	63.2	[50.7; 74.6]	0.5%
Pinzone, 2019	26	72	36.1	[25.1; 48.3]	0.5%
Pool, 2019	229	819	28.0	[24.9; 31.2]	0.5%
Protopopescu, 2012	103	599	17.2	[14.3; 20.5]	0.5%
Quirico, 2018	40	79	50.6	[39.1; 62.1]	0.5%
Quiros-Roldan, 2005	30	36	83.3	[67.2; 93.6]	0.4%
Rabkin, 2018	170	1826	9.3	[8.0; 10.7]	0.5%
Rasmussen, 2016	44	55	80.0	[67.0; 89.6]	0.5%
Rasmussen, 2016	123	182	67.6	[60.3; 74.3]	0.5%
Reid, 2017	71	225	31.6	[25.5; 38.1]	0.5%
Reid, 2019	9	148	6.1	[2.8; 11.2]	0.5%
Rollet-Kurhajec, 2015	235	308	76.3	[71.1; 80.9]	0.5%
Salyer, 2006	39	95	41.1	[31.1; 51.6]	0.5%
Sampériz, 2014	169	275	61.5	[55.4; 67.2]	0.5%
Seang, 2018	11	57	19.3	[10.0; 31.9]	0.5%
Serrano-Villar, 2014	59	132	44.7	[36.0; 53.6]	0.5%
Shirley, 2013	58	200	29.0	[22.8; 35.8]	0.5%
Short, 2014	60	131	45.8	[37.1; 54.7]	0.5%
Shuter, 2008	36	64	56.2	[43.3; 68.6]	0.5%
Siemieniuk, 2011	1042	1946	53.5	[51.3; 55.8]	0.5%
Smit, 2018	1090	8791	12.4	[11.7; 13.1]	0.5%
Socías, 2016	158	397	39.8	[34.9; 44.8]	0.5%
Søgaard, 2010	25	75	33.3	[22.9; 45.2]	0.5%
Soliman, 2015	165	456	36.2	[31.8; 40.8]	0.5%
Spinelli, 2019	45	156	28.8	[21.9; 36.6]	0.5%
Sutton, 2019	14366	26526	54.2	[53.6; 54.8]	0.5%
Sutton, 2019	22070	53052	41.6	[41.2; 42.0]	0.5%
Syed, 2013	17	67	25.4	[15.5; 37.5]	0.5%
Szymanek-Pasternak, 2016	55	98	56.1	[45.7; 66.1]	0.5%
Tarancon-Diez, 2018	103	253	40.7	[34.6; 47.0]	0.5%
Thudium, 2018	315	1082	29.1	[26.4; 31.9]	0.5%
Tomažič, 2007	15	37	40.5	[24.8; 57.9]	0.4%
Tomažič, 2007	17	35	48.6	[31.4; 66.0]	0.4%
Troy, 2016	93	208	44.7	[37.8; 51.7]	0.5%
Troy, 2016	75	191	39.3	[32.3; 46.6]	0.5%
Troy, 2016	26	33	78.8	[61.1; 91.0]	0.4%
Troy, 2016	84	193	43.5	[36.4; 50.8]	0.5%
Turčinov, 2011	65	130	50.0	[41.1; 58.9]	0.5%
van Zoest, 2016	169	499	33.9	[29.7; 38.2]	0.5%
Vernon, 2009	77	101	76.2	[66.7; 84.1]	0.5%
Volpe, 2013	94	211	44.5	[37.7; 51.5]	0.5%
Willig, 2017	20	42	47.6	[32.0; 63.6]	0.4%
Winston, 2013	157	557	28.2	[24.5; 32.1]	0.5%
Wójtowicz, 2019	2103	3495	60.2	[58.5; 61.8]	0.5%
Yuan, 2006	1166	3414	34.2	[32.6; 35.8]	0.5%
Zachry, 2013	49	5530	0.9	[0.7; 1.2]	0.5%
Zhang, 2018	783	1799	43.5	[41.2; 45.9]	0.5%
**Pooled Prevalence**		**293547**	**45.4**	**[42.8; 48.1]**	**100.0%**

Heterogeneity: $I^2 = 99.5\%$, $p = 0$

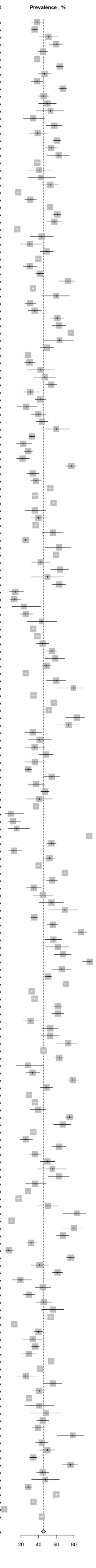

Supplementary Figure 10. Funnel plot of the meta-analysis of association between exposure to active smoking and non-adherence to antiretroviral therapy in people living with HIV, crude analysis

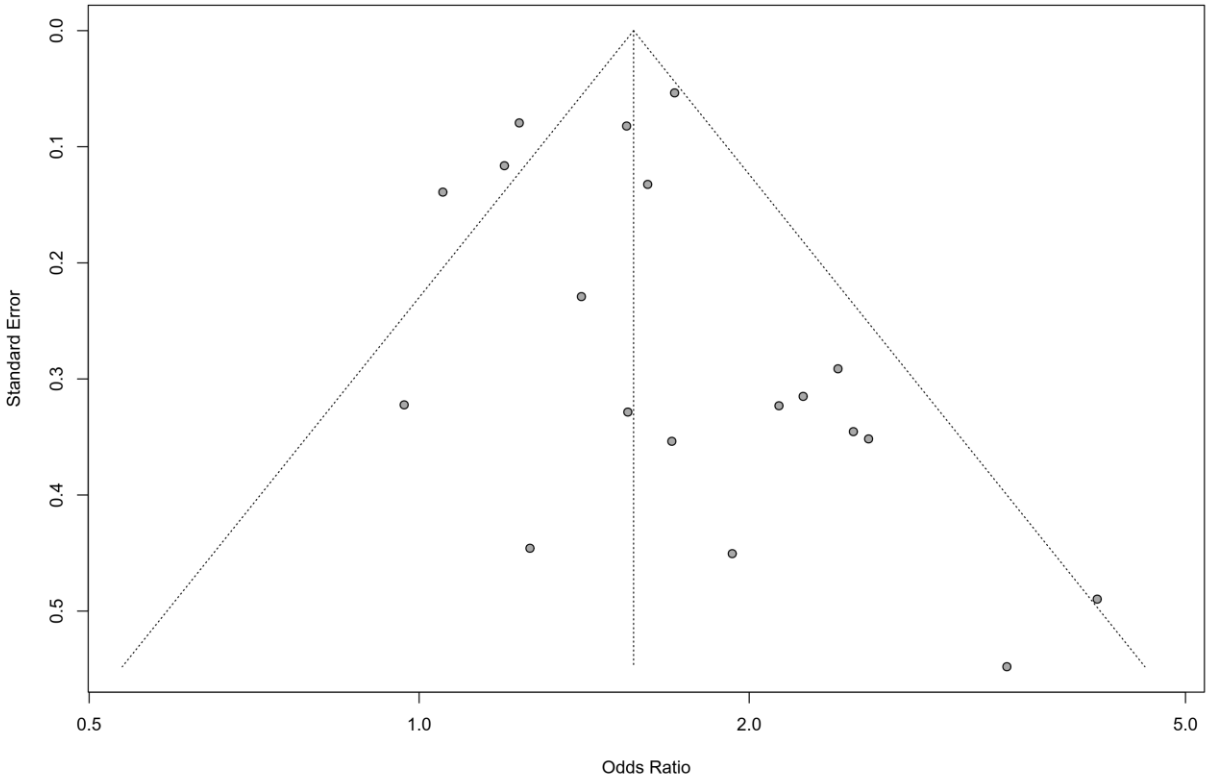

Supplementary Figure 11. Funnel plot of the meta-analysis of association between exposure to active smoking and non-adherence to antiretroviral therapy in people living with HIV, adjusted analysis

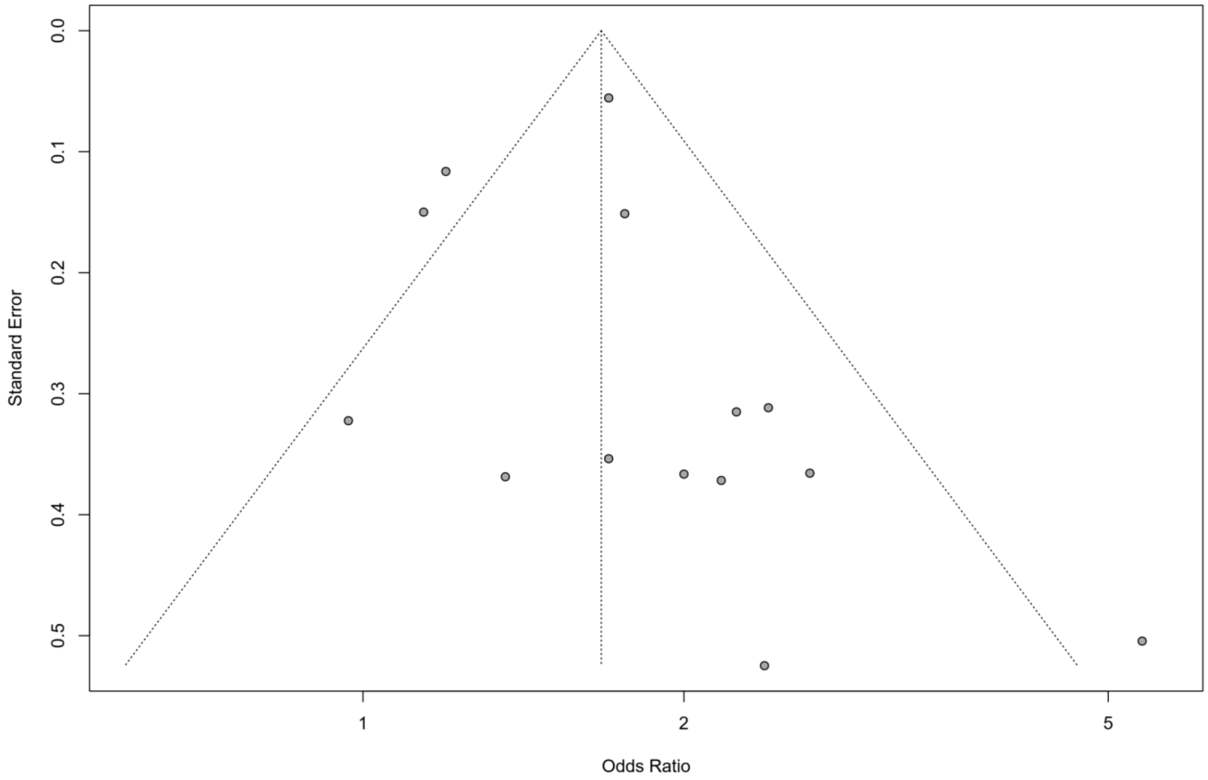

Supplementary References 1. Articles included in the meta-analysis prevalence of active smoking in the global population undergoing antiretroviral therapy

Degroote S., D., Vogelaers, P., Vermeir, A., Mariman, A., De Rick, B., Van Der Gucht, et al.
Determinants of adherence in a cohort of Belgian HIV patients: A pilot study.
Acta Clin Belg. 2014;69(2):111-5.

Chițu-Tișu C.E., E.C., Barbu, M., Lazăr, M., Bojincă, A.-M., Tudor, A., Hristea, et al.
Body composition in HIV-infected patients receiving highly active antiretroviral therapy.
Acta Clin Belg Int J Clin Lab Med. 2017;72(1):55-62.

Gamarel K.E., T.B., Neilands, A.A., Conroy, S.E., Dilworth, N., Lisha, J.M., Taylor, et al.
A longitudinal study of persistent smoking among HIV-positive gay and bisexual men in primary relationships.
Addict Behav. 2017;66:118-24.

Chireshe R., K., Naidoo, R., Nyamakura.
Hypertension among human immunodeficiency virus infected patients on treatment at Parirenyatwa Hospital: A descriptive study.
Afr J Prim Health Care Fam Med. 2019;11(1):e1-e8.

Knudsen A., T.L., Katzenstein, T., Benfield, N.R., Jørgensen, G., Kronborg, J., Gerstoft, et al.
Plasma plasminogen activator inhibitor-1 predicts myocardial infarction in HIV-1-infected individuals.
AIDS. 2014;28(8):1171-9.

Wójtowicz A., S., Bibert, P., Taffé, E., Bernasconi, H., Furrer, H.F., Günthard, et al.
IL-4 polymorphism influences susceptibility to Pneumocystis jirovecii pneumonia in HIV-positive patients.
AIDS. 2019;33(11):1719-27.

Longenecker C.T., C.E., Sullivan, J., Morrison, C.O., Hileman, D.A., Zidar, R., Gilkeson, et al.
The effects of HIV and smoking on aortic and splenic inflammation.
AIDS. 2018;32(1):89-94.

Grint D., L., Peters, J.K., Rockstroh, S., De Wit, V.M., Mitsura, B., Knysz, et al.
Increased incidence of antiretroviral drug discontinuation among patients with viremic hepatitis C virus coinfection and high hyaluronic acid, a marker of liver fibrosis.
AIDS. 2014;28(4):577-87.

Hsu D.C., Y.F., Ma, S., Hur, D., Li, A., Rupert, R., Scherzer, et al.
Plasma IL-6 levels are independently associated with atherosclerosis and mortality in HIV-infected individuals on suppressive antiretroviral therapy.
AIDS. 2016;30(13):2065-74.

Boccara F., T., Simon, K., Lacombe, A., Cohen, B., Laloux, E., Bozec, et al.
Influence of pravastatin on carotid artery structure and function in dyslipidemic HIV-infected patients receiving antiretroviral therapy.
AIDS. 2006;20(18):2395-8.

Palella F.J., X., Li, S.K., Gupta, M.M., Estrella, J.P., Phair, J.B., Margolick, et al.
Long-Term kidney function, proteinuria, and associated risks among HIV-infected and uninfected men.
AIDS. 2018;32(10):1247-56.

Kwong G.P.S., A.C., Ghani, R.A., Rode, L.M., Bartley, B.J., Cowling, B., Da Silva, et al.
Comparison of the risks of atherosclerotic events versus death from other causes associated with antiretroviral use.
AIDS. 2006;20(15):1941-50.

Lo J., S., Abbara, L., Shturman, A., Soni, J., Wei, J.A., Rocha-Filho, et al.
Increased prevalence of subclinical coronary atherosclerosis detected by coronary computed tomography angiography in hiv-infected men.
AIDS. 2010;24(2):243-53.

Ho J.E., R., Scherzer, F.M., Hecht, K., Maka, V., Selby, J.N., Martin, et al.

The association of CD4+ T-cell counts and cardiovascular risk in treated HIV disease.
AIDS. 2012;26(9):1115-20.

O'Halloran J.A., S.A., Cooley, J.F., Strain, A., Boerwinkle, R., Paul, R.M., Presti, et al.
Altered neuropsychological performance and reduced brain volumetrics in people living with HIV on integrase strand transfer inhibitors.
AIDS. 2019;33(9):1477-83.

Ho J.E., R., Scherzer, F.M., Hecht, K., Maka, V., Selby, J.N., Martin, et al.
The association of CD4+ T-cell counts and cardiovascular risk in treated HIV disease.
AIDS. 2012;26(9):1115-20.

Lake J.E., X., Li, F.J., Palella, K.M., Erlandson, D., Wiley, L., Kingsley, et al.
Metabolic health across the BMI spectrum in HIV-infected and HIV-uninfected men.
AIDS. 2018;32(1):49-57.

Koethe J.R., H., Grome, C.A., Jenkins, S.A., Kalams, T.R., Sterling.
The metabolic and cardiovascular consequences of obesity in persons with HIV on long-term antiretroviral therapy.
AIDS. 2016;30(1):83-91.

Kooij K.W., F.W.N.M., Wit, J., Schouten, M., Van Der Valk, M.H., Godfried, I.G., Stolte, et al.
HIV infection is independently associated with frailty in middle-aged HIV type 1-infected individuals compared with similar but uninfected controls.
AIDS. 2016;30(2):241-50.

Ryom L., J.D., Lundgren, M., Law, O., Kirk, W., El-Sadr, F., Bonnet, et al.
Serious clinical events in HIV-positive persons with chronic kidney disease (CKD).
AIDS. 2019.

Tarancon-Diez L., R.S., De Pablo-Bernal, J.L., Jiménez, A.I., Álvarez-Ríos, M., Genebat, I., Rosado-Sánchez, et al.
Role of toll-like receptor 4 Asp299Gly polymorphism in the development of cardiovascular diseases in HIV-infected patients.
AIDS. 2018;32(8):1035-41.

Conley L.J., T.J., Bush, A.W., Rupert, I., Sereti, P., Patel, J.T., Brooks, et al.
Obesity is associated with greater inflammation and monocyte activation among HIV-infected adults receiving antiretroviral therapy.
AIDS. 2015;29(16):2201-7.

Depairon M., S., Chessex, P., Sudre, N., Rodondi, N., Doser, J.-P., Chave, et al.
Premature atherosclerosis in hiv-infected individuals focus on protease inhibitor therapy.
AIDS. 2001;15(3):329-34.

Gaisa M., K., Sigel, J., Hand, S., Goldstone.
High rates of anal dysplasia in HIV-infected men who have sex with men, women, and heterosexual men.
AIDS. 2014;28(2):215-22.

Helleberg M., M.T., May, S.M., Ingle, F., Dabis, P., Reiss, G., Fätkenheuer, et al.
Smoking and life expectancy among HIV-infected individuals on antiretroviral therapy in Europe and North America.
AIDS. 2015;29(2):221-9.

Rabkin M., A., Palma, M.L., McNairy, A.B., Gachuhi, S., Simelane, H., Nuwagaba-Biribonwoha, et al.
Integrating cardiovascular disease risk factor screening into HIV services in Swaziland: Lessons from an implementation science study.
AIDS. 2018;32:S43-S6.

Shahmanesh M., A., Schultze, F., Burns, O., Kirk, J., Lundgren, C., Mussini, et al.

The cardiovascular risk management for people living with HIV in Europe: How well are we doing?
AIDS. 2016;30(16):2505-18.

Spinelli M.A., N.A., Hessol, S., Schwarcz, L., Hsu, M.-K., Parisi, S., Pipkin, et al.
Homelessness at diagnosis is associated with death among people with HIV in a population-based study of a US city.
AIDS. 2019;33(11):1789-94.

Søgaard O.S., H.C., Schønheyder, A.R., Bukh, Z.B., Harboe, T.A., Rasmussen, L., Østergaard, et al.
Pneumococcal conjugate vaccination in persons with HIV: The effect of highly active antiretroviral therapy.
AIDS. 2010;24(9):1315-22.

Maggi P., A., Lillo, F., Perilli, R., Maserati, A., Chirianni.
Colour-Doppler ultrasonography of carotid vessels in patients treated with antiretroviral therapy: A comparative study.
AIDS. 2004;18(7):1023-8.

Maggi P., G., Serio, G., Epifani, G., Fiorentino, A., Saracino, C., Fico, et al.
Premature lesions of the carotid vessels in HIV-1-infected patients treated with protease inhibitors.
AIDS. 2000;14(16):F123-F8.

Crane H.M., M.E., McCaul, G., Chander, H., Hutton, R.M., Nance, J.A.C., Delaney, et al.
Prevalence and Factors Associated with Hazardous Alcohol Use Among Persons Living with HIV Across the US in the Current Era of Antiretroviral Treatment.
AIDS Behav. 2017;21(7):1914-25.

Pacek L.R., C., Latkin, R.M., Crum, E.A., Stuart, A.R., Knowlton.
Current cigarette smoking among HIV-positive current and former drug users: associations with individual and social characteristics.
AIDS Behav. 2014;18(7):1368-77.

Cioe P.A., K.E., Gamarel, D.W., Pantalone, P.M., Monti, K.H., Mayer, C.W., Kahler.
Cigarette Smoking and Antiretroviral Therapy (ART) Adherence in a Sample of Heavy Drinking HIV-Infected Men Who Have Sex with Men (MSM).
AIDS Behav. 2017;21(7):1956-63.

Zhang Y., T.E., Wilson, A., Adedimeji, D., Merenstein, J., Milam, J., Cohen, et al.
The Impact of Substance Use on Adherence to Antiretroviral Therapy Among HIV-Infected Women in the United States.
AIDS Behav. 2018;22(3):896-908.

Anema A., S.D., Weiser, K.A., Fernandes, E., Ding, E.K., Brandson, A., Palmer, et al.
High prevalence of food insecurity among HIV-infected individuals receiving HAART in a resource-rich setting.
AIDS Care Psychol Socio-Med Asp AIDS HIV. 2011;23(2):221-30.

Abioye A.I., S., Isanaka, E., Liu, R.S., Mwiru, R.A., Noor, D., Spiegelman, et al.
Gender differences in diet and nutrition among adults initiating antiretroviral therapy in Dar es Salaam, Tanzania.
AIDS Care Psychol Socio-Med Asp AIDS HIV. 2015;27(6):706-15.

Ghadaki B., N., Kronfli, T., Vanniyasingam, S., Haider.
Chronic obstructive pulmonary disease and HIV: are we appropriately screening?
AIDS Care Psychol Socio-Med Asp AIDS HIV. 2016;28(10):1338-43.

Marando F., G., Gualberti, A.M., Costanzo, U., Di Luzio Paparatti, M., Franzetti, A., Ammassari, et al.
Discrepancies between physician's perception of depression in HIV patients and self-reported CES-D-20 assessment: The DHIVA study.
AIDS Care Psychol Socio-Med Asp AIDS HIV. 2016;28(2):147-59.
Monnig M.A., C.W., Kahler, H., Lee, D.W., Pantalone, K.H., Mayer, R.A., Cohen, et al.
Effects of smoking and alcohol use on neurocognitive functioning in heavy drinking, HIV-positive men who have sex with men.

AIDS Care Psychol Socio-Med Asp AIDS HIV. 2016;28(3):300-5.

Nguyen N.T.P., B.X., Tran, L.Y., Hwang, C.M., Markham, M.D., Swartz, J.I., Vidrine, et al.
Effects of cigarette smoking and nicotine dependence on adherence to antiretroviral therapy among HIV-positive patients in Vietnam.
AIDS Care Psychol Socio-Med Asp AIDS HIV. 2016;28(3):359-64.

Nolan S., A.Y., Walley, T.C., Heeren, G.J., Patts, A.S., Ventura, M.M., Sullivan, et al.
HIV-infected individuals who use alcohol and other drugs, and virologic suppression.
AIDS Care Psychol Socio-Med Asp AIDS HIV. 2017;29(9):1129-36.

Muyanja D., C., Muzoora, A., Muyingo, W., Muyindike, M.J., Siedner.
High Prevalence of Metabolic Syndrome and Cardiovascular Disease Risk among People with HIV on Stable ART in Southwestern Uganda.
AIDS Patient Care STDs. 2016;30(1):4-10.

Shirley D.K., R.K., Kesari, M.J., Glesby.
Factors associated with smoking in HIV-infected patients and potential barriers to cessation.
AIDS Patient Care STDs. 2013;27(11):604-12.

Leung J.M., J.C., Liu, A., Mtambo, D., Ngan, N., Nashta, S., Guillemi, et al.
The determinants of poor respiratory health status in adults living with human immunodeficiency virus infection.
AIDS Patient Care STDs. 2014;28(5):240-7.

Krsak M., D.M., Kent, N., Terrin, C., Holcroft, S.C., Skinner, C., Wanke.
Myocardial Infarction, Stroke, and Mortality in cART-Treated HIV Patients on Statins.
AIDS Patient Care STDs. 2015;29(6):307-13.

Reid M.J.A., Y., Ma, I., Golovaty, S., Okello, R., Sentongo, M., Feng, et al.
Association of Gut Intestinal Integrity and Inflammation with Insulin Resistance in Adults Living with HIV in Uganda.
AIDS Patient Care STDs. 2019;33(7):299-307.

Donald K.A.M., A., Fernandez, K., Claborn, C., Kuo, N., Koen, H., Zar, et al.
The developmental effects of HIV and alcohol: a comparison of gestational outcomes among babies from South African communities with high prevalence of HIV and alcohol use.
AIDS Res Ther. 2017;14(1):28.

Camargo C.C., N.R.V., Cavassan, K.I., Tasca, S., Meneguin, H.A., Miot, L.R., Souza.
Depression and Coping Are Associated with Failure of Adherence to Antiretroviral Therapy Among People Living with HIV/AIDS.
AIDS Res Hum Retroviruses. 2019.

Hileman C.O., D.A., Wohl, D.J., Tisch, S.M., Debanne, G.A., McComsey.
Short communication: Initiation of an abacavir-containing regimen in HIV-infected adults is associated with a smaller decrease in inflammation and endothelial activation markers compared to non-abacavir-containing regimens.
AIDS Res Hum Retroviruses. 2012;28(12):1561-4.

Hileman C.O., G.A., McComsey.
Short Communication: The Effect of Rosuvastatin on Vascular Disease Differs by Smoking Status in Treated HIV Infection.
AIDS Res Hum Retroviruses. 2018;34(3):282-5.

Morell E.B., J.S., Cabeza, A., Muñoz, I., Marín, M., Masiá, F., Gutiérrez, et al.
The CD4/CD8 Ratio is Inversely Associated with Carotid Intima-Media Thickness Progression in Human Immunodeficiency Virus-Infected Patients on Antiretroviral Treatment.
AIDS Res Hum Retroviruses. 2016;32(7):648-53.

Grome H.N., L., Barnett, C.C., Hagar, D.G., Harrison, S.A., Kalams, J.R., Koethe.

Association of T Cell and Macrophage Activation with Arterial Vascular Health in HIV.
AIDS Res Hum Retroviruses. 2017;33(2):181-6.

Koethe J.R., K., Dee, A., Bian, A., Shintani, M., Turner, S., Bebawy, et al.
Circulating interleukin-6, soluble CD14, and other inflammation biomarker levels differ between obese and nonobese HIV-infected adults on antiretroviral therapy.
AIDS Res Hum Retroviruses. 2013;29(7):1019-25.

Levy M.E., A.E., Greenberg, M., Magnus, N., Younes, A., Castel.
Immunosuppression and HIV Viremia Associated with More Atherogenic Lipid Profile in Older People with HIV.
AIDS Res Hum Retroviruses. 2019;35(1):81-91.

Hoffman R.M., J.E., Lake, H.M., Wilhalme, C.-H., Tseng, J.S., Currier.
Vitamin D levels and markers of inflammation and metabolism in HIV-infected individuals on suppressive antiretroviral therapy.
AIDS Res Hum Retroviruses. 2016;32(3):247-54.

Seang S., T., Kelesidis, D., Huynh, S., Park, A.A., Moe, J.S., Currier, et al.
Low Levels of Endothelial Progenitor Cells and Their Association with Systemic Inflammation and Monocyte Activation in Older HIV-Infected Men.
AIDS Res Hum Retroviruses. 2018;34(1):39-45.

Syed S.S., R.S., Balluz, E.K., Kabagambe, W.A., Meyer, S., Lukas, C.M., Wilson, et al.
Assessment of biomarkers of cardiovascular risk among HIV type 1-infected adolescents: Role of soluble vascular cell adhesion molecule as an early indicator of endothelial inflammation.
AIDS Res Hum Retroviruses. 2013;29(3):493-500.

Carballo D., C., Delhumeau, S., Carballo, C., Bähler, D., Radovanovic, B., Hirschel, et al.
Increased mortality after a first myocardial infarction in human immunodeficiency virus-infected patients; a nested cohort study.
AIDS Res Ther. 2015;12(1).

Mashinya F., M., Alberts, J.-P., Van geertruyden, R., Colebunders.
Assessment of cardiovascular risk factors in people with HIV infection treated with ART in rural South Africa: A cross sectional study.
AIDS Res Ther. 2015;12(1).

Hidalgo-Tenorio C., S.E., de Jesus, J., Esquivias, J., Pasquau.
High prevalence and incidence of HPV-related anal cancer precursor lesions in HIV-positive women in the late HAART era.
Alta prevalencia e incidencia de lesiones precursoras de cáncer anal asociada a la infección por VPH en mujeres VIH positivas en la era tardía del TAR. 2018;36(9):555-62.

Okafor C.N., Z., Zhou, L.E., Burrell, N.E., Kelso, N.E., Whitehead, J.S., Harman, et al.
Marijuana use and viral suppression in persons receiving medical care for HIV-infection.
Am J Drug Alcohol Abuse. 2017;43(1):103-10.

Bertisch B., S., Franceschi, M., Lise, P., Vernazza, O., Keiser, F., Schöni-Affolter, et al.
Risk factors for anal cancer in persons infected with HIV: a nested case-control study in the Swiss HIV Cohort Study.
Am J Epidemiol. 2013;178(6):877-84.

Iyer J.R., A., Van Rie, S.A., Haberlen, M., Mudavanhu, L., Mutunga, J., Bassett, et al.
Subfertility among HIV-affected couples in a safer conception cohort in South Africa.
Am J Obstet Gynecol. 2019;221(1):48.e1-.e18.

Haeri S., M., Shauer, M., Dale, J., Leslie, A.M., Baker, S., Saddlemire, et al.
Obstetric and newborn infant outcomes in human immunodeficiency virus-infected women who receive highly active antiretroviral therapy.
Am J Obstet Gynecol. 2009;201(3):315.e1-.e5.

Iqbal S.N., J., Kriebs, C., Harman, L., Alger, J., Kopelman, O., Turan, et al.
Predictors of fetal growth in maternal HIV disease.
Am J Perinatol. 2010;27(7):517-23.

Mercié P., R., Thiébaut, V., Lavignolle, J.-L., Pellegrin, M.-C., Yvorra-Vives, P., Morlat, et al.
Evaluation of cardiovascular risk factors in HIV-1 infected patients using carotid intima-media thickness measurement.
Ann Med. 2002;34(1):55-63.

Morillo-Verdugo R., M.D.L.A., Robustillo-Cortés, M.T., Martín-Conde, G., Callejón-Callejón, P., Cid-Silva, C., Moriel-Sánchez, et al.
Effect of a Structured Pharmaceutical Care Intervention Versus Usual Care on Cardiovascular Risk in HIV Patients on Antiretroviral Therapy: INFAMERICA Study.
Ann Pharmacother. 2018;52(11):1098-108.

Bijker R., N., Kumarasamy, S., Kiertiburanakul, S., Pujari, W., Lam, R., Chaiwarith, et al.
Cardiovascular disease incidence projections in the TREAT Asia HIV Observational Database (TAHOD).
Antivir Ther (Lond). 2019.

Protopopescu C., F., Raffi, C., Brunet-François, D., Salmon, R., Verdon, P., Reboud, et al.
Incidence, medical and socio-behavioural predictors of psychiatric events in an 11-year follow-up of HIV-infected patients on antiretroviral therapy.
Antiviral Ther. 2012;17(6):1079-83.

Zannou D.M., L., Denoeud, K., Lacombe, D., Amoussou-Guenou, J., Bashi, J., Akakpo, et al.
Incidence of lipodystrophy and metabolic disorders in patients starting non-nucleoside reverse transcriptase inhibitors in Benin.
Antiviral Ther. 2009;14(3):371-80.

Buchacz K., R.K., Baker, F.J., Palella Jr., L., Shaw, P., Patel, K.A., Lichtenstein, et al.
Disparities in prevalence of key chronic diseases by gender and race/ethnicity among antiretroviraltreated HIV-infected adults in the US.
Antiviral Ther. 2013;18(1):65-75.

Dubé M.P., J.W., Wu, J.A., Aberg, M.A., Deeg, B.L., Alston-Smith, M.E., McGovern, et al.
Safety and efficacy of extended-release niacin for the treatment of dyslipidaemia in patients with HIV infection: AIDS Clinical Trials Group Study A5148.
Antiviral Ther. 2006;11(8):1081-9.

Boger M.S., A., Bian, A., Shintani, G.L., Milne, J.D., Morrow, H., Erdem, et al.
Sex differences in urinary biomarkers of vascular and endothelial function in HIV-infected persons receiving antiretroviral therapy.
Antiviral Ther. 2012;17(3):485-93.

Park M.S., C.O., Hileman, A., Sattar, R., Gilkeson, G.A., McComsey.
Incidental findings on chest computed tomography are common and linked to inflammation in HIV-infected adults.
Antiviral Ther. 2017;22(2):127-33.

Llop M., W.A., Sifuentes, S., Bañón, C., Macia-Villa, M.J., Perez-Elías, M., Rosillo, et al.
Increased prevalence of asymptomatic vertebral fractures in HIV-infected patients over 50 years of age.
Arch Osteoporosis. 2018;13(1).

Monteiro V.S., H.R., Lacerda, M., Uellendahl, T.M., Chang, V.M., de Albuquerque, J.C., Zirpoli, et al.
Calcium score in the evaluation of atherosclerosis in patients with HIV/Aids.
Arq Bras Cardiol. 2011;97(5):427-33.

Arbune M., I.-M., Dumitru, M., Cretu-Stuparu.
Characteristics of sleep disorders in Romanian adults infected with human immunodeficiency virus.
ARS Medica Tomitana. 2017;23(3):126-31.

Batista J.L., M.F.P.M., de Albuquerque, M.L., Santos, D.B., Miranda-Filho, H.R., Lacerda, M., Maruza, et al.
Association between smoking, crack cocaine abuse and the discontinuation of combination antiretroviral therapy in recife, pernambuco, Brazil.
Associação entre tabagismo e o uso de crack com a descontinuidade da terapia antirretroviral combinada em Recife, Pernambuco, Brasil. 2014;56(2):127-32.

Lima E.M.O., D.M., Gualandro, P.C., Yu, I.d.C.B., Giuliano, A.C., Marques, D., Calderaro, et al.
Cardiovascular prevention in HIV patients: Results from a successful intervention program.
Atherosclerosis. 2009;204(1):229-32.

Acevedo M., D.L., Sprecher, L., Calabrese, G.L., Pearce, D.L., Coyner, S.S., Halliburton, et al.
Pilot study of coronary atherosclerotic risk and plaque burden in HIV patients: 'A call for cardiovascular prevention'.
Atherosclerosis. 2002;163(2):349-54.

Parikh N.I., M., Gerschenson, K., Bennett, L.M.M., Gangcuangco, M.S., Lopez, N.N., Mehta, et al.
Lipoprotein concentration, particle number, size and cholesterol efflux capacity are associated with mitochondrial oxidative stress and function in an HIV positive cohort.
Atherosclerosis. 2015;239(1):50-4.

Pinzone M.R., M., Ceccarelli, E.V., Rullo, M., Maresca, R., Bruno, F., Condorelli, et al.
Circulating angiopoietin-like protein 2 levels are associated with decreased renal function in HIV+ subjects on cART: A potential marker of kidney disease.
Biomed Rep. 2019;10(2):140-4.

Misra R., P., Chandra, S.E., Riechman, D.M., Long, S., Shinde, H.J., Pownall, et al.
Relationship of ethnicity and CD4 Count with glucose metabolism among HIV patients on Highly-Active Antiretroviral Therapy (HAART).
BMC Endocr Disord. 2013;13.

PrayGod G., J., Changalucha, S., Kapiga, R., Peck, J., Todd, S., Filteau.
Dysglycemia associations with adipose tissue among HIV-infected patients after 2 years of antiretroviral therapy in Mwanza: A follow-up cross-sectional study.
BMC Infect Dis. 2017;17(1).

Siemieniuk R.A.C., D.B., Gregson, M.J., Gill.
The persisting burden of invasive pneumococcal disease in HIV patients: An observational cohort study.
BMC Infect Dis. 2011;11.

George S., N., McGrath, T., Oni.
The association between a detectable HIV viral load and non-communicable diseases comorbidity in HIV positive adults on antiretroviral therapy in Western Cape, South Africa.
BMC Infect Dis. 2019;19(1).

Ansemant T., S., Mahy, C., Piroth, P., Ornetti, S., Ewing, J.-C., Guilland, et al.
Severe hypovitaminosis D correlates with increased inflammatory markers in HIV infected patients.
BMC Infect Dis. 2013;13(1).

Bekolo C.E., M.B., Nguena, L., Ewane, P.S., Bekoule, B., Kollo.
The lipid profile of HIV-infected patients receiving antiretroviral therapy in a rural Cameroonian population.
BMC Public Health. 2014;14:236.

Bailey H., C., Thorne, R., Malyuta, C.L., Townsend, I., Semenenko, M., Cortina-Borja.
Adherence to antiretroviral therapy during pregnancy and the first year postpartum among HIV-positive women in Ukraine.
BMC Public Health. 2014;14:993.

Carrieri M.P., C., Protopopescu, V., Le Moing, P., Reboud, F., Raffi, S., Mahy, et al.
Impact of immunodepression and moderate alcohol consumption on coronary and other arterial disease events in

an 11-year cohort of HIV-infected patients on antiretroviral therapy.
BMJ Open. 2012;2(6).

Alemu Y.M., W., Awoke, A., Wilder-Smith.
Determinants for tuberculosis in HIV-infected adults in Northwest Ethiopia:multicentre case-control Study.
BMJ Open. 2016;6(4).

Cahn P., O., Leite, A., Rosales, R., Cabello, C.A., Alvarez, C., Seas, et al.
Metabolic profile and cardiovascular risk factors among Latin American HIV-infected patients receiving HAART.
Braz J Infect Dis. 2010;14(2):158-66.

De Fátima Bonolo P., C.J., Machado, C.C., César, M., Das Graças Braga Ceccato, M.D.C., Guimarães.
Vulnerability and non-adherence to antiretroviral therapy among HIV patients, Minas Gerais State, Brazil.
Cad Saude Publica. 2008;24(11):2603-13.

Bolland M.J., A.M., Horne, S.E., Briggs, M.G., Thomas, I.R., Reid, G.D., Gamble, et al.
Long-Term Stable Bone Mineral Density in HIV-Infected Men Without Risk Factors for Osteoporosis Treated with Antiretroviral Therapy.
Calcif Tissue Int. 2019;105(4):423-9.

Bonnet F., C., Lewden, T., May, L., Heripret, E., Jougla, S., Bevilacqua, et al.
Malignancy-related causes of death in human immunodeficiency virus-infected patients in the era of highly active antiretroviral therapy.
Cancer. 2004;101(2):317-24.

Costiniuk C.T., Z., Saneei, S., Salahuddin, J., Cox, J.-P., Routy, S., Rueda, et al.
Cannabis Consumption in People Living with HIV: Reasons for Use, Secondary Effects, and Opportunities for Health Education.
Cannabis and Cannabinoid Res. 2019;4(3):204-13.

Rezaei-Soufi L., P., Davoodi, H.R., Abdolsamadi, M., Jazaeri, H., Malekzadeh.
Dental caries prevalence in Human Immunodeficiency Virus infected patients receiving highly active anti-retroviral therapy in Kermanshah, Iran.
Cell J. 2014;16(1):73-8.

Kesselring A., L., Gras, C., Smit, G., Van Twillert, A., Verbon, F., De Wolf, et al.
Immunodeficiency as a risk factor for non-AIDS-defining malignancies in HIV-1-infected patients receiving combination antiretroviral therapy.
Clin Infect Dis. 2011;52(12):1458-65.

Kerchberger A.M., A.N., Sheth, C.D., Angert, C.C., Mehta, N.A., Summers, I., Ofotokun, et al.
Weight Gain Associated with Integrase Stand Transfer Inhibitor Use in Women.
Clin Infect Dis. 2019.

Lorenz D.R., A., Dutta, S.S., Mukerji, A., Holman, H., Uno, D., Gabuzda.
Marijuana Use Impacts Midlife Cardiovascular Events in HIV-Infected Men.
Clin Infect Dis. 2017;65(4):626-35.

Cole J.H., M.W.A., Caan, J., Underwood, D., De Francesco, R.A., Van Zoest, F.W.N.M., Wit, et al.
No Evidence for Accelerated Aging-Related Brain Pathology in Treated Human Immunodeficiency Virus: Longitudinal Neuroimaging Results from the Comorbidity in Relation to AIDS (COBRA) Project.
Clin Infect Dis. 2018;66(12):1899-909.

Smit M., R.A., van Zoest, B.E., Nichols, I., Vaartjes, C., Smit, M., van der Valk, et al.
Cardiovascular disease prevention policy in human immunodeficiency virus: Recommendations from a modeling study.
Clin Infect Dis. 2018;66(5):743-50.

Johs N.A., K., Wu, K., Tassiopoulos, S.L., Koletar, R.C., Kalayjian, R.J., Ellis, et al.
Disability among Middle-Aged and Older Persons with Human Immunodeficiency Virus Infection.

Clin Infect Dis. 2017;65(1):83-91.

van Zoest R.A., F.W., Wit, K.W., Kooij, M., van der Valk, J., Schouten, N.A., Kootstra, et al.
Higher Prevalence of Hypertension in HIV-1-Infected Patients on Combination Antiretroviral Therapy Is Associated With Changes in Body Composition and Prior Stavudine Exposure.
Clin Infect Dis. 2016;63(2):205-13.

Bonolo P.F., M.G.B., Ceccato, G.M., Rocha, F.A., Acúrcio, L.N., Campos, M.D., Crosland Guimarães.
Gender differences in non-adherence among Brazilian patients initiating antiretroviral therapy.
Clinics. 2013;68(5):612-20.

Vernon L.T., C.A., Demko, C.C., Whalen, M.M., Lederman, Z., Toossi, M., Wu, et al.
Characterizing traditionally defined periodontal disease in HIV+ adults.
Community Dent Oral Epidemiol. 2009;37(5):427-37.

Begovac J., G., Dragović, K., Višković, J., Kušić, M.P., Mihanović, D., Lukas, et al.
Comparison of four international cardiovascular disease prediction models and the prevalence of eligibility for lipid lowering therapy in HIV infected patients on antiretroviral therapy.
Croat Med J. 2015;56(1):14-23.

Sutton S.S., J., Magagnoli, T.H., Cummings, J.W., Hardin, B., Edun, A., Beaubrun.
Chronic kidney disease, cardiovascular disease, and osteoporotic fractures in patients with and without HIV in the US Veteran's Affairs Administration System.
Curr Med Res Opin. 2019;35(1):117-25.

Bednasz C., A.E., Luque, B.S., Zingman, M.A., Fischl, B.M., Gripshover, C.S., Venuto, et al.
Lipid-lowering therapy in HIV-infected patients: Relationship with antiretroviral agents and impact of substance-related disorders.
Curr Vasc Pharmacol. 2016;14(3):280-7.

Worm S.W., C.A., Sabin, P., Reiss, W., El-Sadr, A.D., Monforte, C., Pradier, et al.
Presence of the metabolic syndrome is not a better predictor of cardiovascular disease than the sum of its components in HIV-infected individuals.
Diabetes Care. 2009;32(3):474-80.

Kobayashi T., K., Sigel, T., Kalir, I.J., MacLeod, Y., Liu, M., Gaisa.
Anal cancer precursor lesions in HIV-infected persons: Tissue human papillomavirus type distribution and impact on treatment response.
Dis Colon Rectum. 2019;62(5):579-85.

Socías M.E., E., Wood, W., Small, H., Dong, J., Shoveller, T., Kerr, et al.
Methadone maintenance therapy and viral suppression among HIV-infected opioid users: The impacts of crack and injection cocaine use.
Drug Alcohol Depend. 2016;168:211-8.

Marcellin F., C., Lions, E., Rosenthal, P., Roux, P., Sogni, L., Wittkop, et al.
No significant effect of cannabis use on the count and percentage of circulating CD4 T-cells in HIV-HCV co-infected patients (ANRS CO13-HEPAVIH French cohort).
Drug Alcohol Rev. 2017;36(2):227-38.

North C.M., L., Valeri, P.W., Hunt, A.R., Mocello, J.N., Martin, Y., Boum, et al.
Cooking fuel and respiratory symptoms among people living with HIV in rural Uganda.
ERJ Open Res. 2017;3(2).

Friis-Møller N., R., Thiébaut, P., Reiss, R., Weber, A.D., Monforte, S., De Wit, et al.
Predicting the risk of cardiovascular disease in HIV-infected patients: The Data collection on Adverse Effects of Anti-HIV Drugs Study.
Eur J Cardiovasc Prev Rehabil. 2010;17(5):491-501.

León R., S., Reus, N., López, I., Portilla, J., Sánchez-Payá, L., Giner, et al.

Subclinical atherosclerosis in low Framingham risk HIV patients.
Eur J Clin Invest. 2017;47(8):591-9.

Bergersen B.M., L., Sandvik, J.N., Bruun, S., Tonstad.
Elevated Framingham risk in HIV-positive on highly active antiretroviral therapy: Results from a Norwegian study of 721 subjects.
Eur J Clin Microbiol Infect Dis. 2004;23(8):625-30.

Berg T., D., Zdunek, J., Stalke, S., Dupke, A., Baumgarten, A., Carganico, et al.
N-terminal pro-B-type natriuretic peptide (NT-proBNP) in HIV-1 infected individuals on HAART.
Eur J Med Res. 2007;12(4):152-60.

Neumann T., T., Woiwod, A., Neumann, B., Ross, C., Von Birgelen, L., Volbracht, et al.
Cardiovascular risk factors and probability for cardiovascular events in HIV-infected patients. Part II: gender differences.
Eur J Med Res. 2004;9(2):55-60.

Fricke U., W., Geurtsen, I., Staufenbiel, A., Rahman.
Periodontal status of HIV-infected patients undergoing antiretroviral therapy compared to HIV-therapy naive patients: a case control study.
Eur J Med Res. 2012;17:2.

Galán R.J., I.M.M., Escalante, R.M., Verdugo.
Influence of pharmacotherapy complexity on compliance with the therapeutic objectives for HIV+ patients on antiretroviral treatment concomitant with therapy for dyslipidemia. INCOFAR Project.
Farm Hosp. 2016;40(2):90-101.

Brites-Alves C., E., Luz, E.M., Netto, T., Ferreira, R.S., Diaz, C., Pedroso, et al.
Immune activation, proinflammatory cytokines, and conventional risks for cardiovascular disease in HIV patients: A case-control study in Bahia, Brazil.
Front Immunol. 2018;9.

Zachry III W.M., J.M., Griffith, C.M., Wegzyn, R., D'Amico, W.C., Woodward, Q.X., Qian.
Cardiovascular risk among HIV-positive subjects preceding exposure to HAART: A retrospective claims analysis.
Future Virol. 2013;8(9):849-60.

Chiţu-Tişu C.-E., E.-C., Barbu, M., Lazăr, D.A., Ion, I.A., Bădărău.
Low bone mineral density and associated risk factors in HIV-infected patients.
GERMS. 2016;6(2):50-9.

Daglan E., D., Yamin, B., Manu, A., Streinu-Cercel.
Cardiac involvement in HIV-positive patients.
GERMS. 2013;3(1):8-13.

Nkuize M., S., De Wit, V., Muls, R., Ntounda, M., Gomez-Galdon, M., Buset.
Comparison of Demographic Characteristics and Upper Gastrointestinal Endoscopy Findings in HIV-Positive, Antiretroviral-Treated Patients With and Without Helicobacter pylori Coinfection.
Helicobacter. 2012;17(2):153-9.

Jackiewicz A., M., Czarnecki, A., Przeliorz-Pyszczek, B., Knysz.
Effect of fatty acid content in the diet on lipid profile in HIV-infected patients treated with antiretroviral drugs.
HIV AIDS Rev. 2019;18(1):25-32.

Dentone C., A., Di Biagio, A., Cozzi Lepri, D., Fenoglio, G., Filaci, M., Lichtner, et al.
Inflammatory effects of atazanavir/ritonavir versus darunavir/ritonavir in treatment naïve, HIV-1-infected patients.
HIV Clin Trials. 2018;19(4):158-62.

Quirico M., R., Valeria, M., Lorenza, P., Umberto, M., Laura, P.M., Cristina, et al.
Bone mass preservation with high-dose cholecalciferol and dietary calcium in HIV patients following antiretroviral therapy. Is it possible?

HIV Clin Trials. 2018.

Önen N., E., Overton, W., Seyfried, E., Stumm, M., Snell, K., Mondy, et al.
Aging and HIV infection: A comparison between older HIV-infected persons and the general population.
HIV Clin Trials. 2010;11(2):100-9.

Carr A., B., Grund, J., Neuhaus, A., Schwartz, J., Bernardino, D., White, et al.
Prevalence of and risk factors for low bone mineral density in untreated HIV infection: A substudy of the INSIGHT Strategic Timing of AntiRetroviral Treatment (START) trial.
HIV Med. 2015;16:137-46.

Lifson A., G., Grandits, E., Gardner, M., Wolff, P., Pulik, I., Williams, et al.
Quality of life assessment among HIV-positive persons entering the INSIGHT Strategic Timing of AntiRetroviral Treatment (START) trial.
HIV Med. 2015;16:88-96.

Niewoehner D., G., Collins, D., Nixon, E., Tedaldi, C., Akolo, C., Kityo, et al.
Pulmonary function in an international sample of HIV-positive, treatment-naïve adults with CD4 counts >500 cells/μL: A substudy of the INSIGHT Strategic Timing of AntiRetroviral Treatment (START) trial.
HIV Med. 2015;16:119-28.

Soliman E., S., Sharma, K., Arastéh, D., Wohl, A., Achhra, G., Tambussi, et al.
Baseline cardiovascular risk in the INSIGHT Strategic Timing of AntiRetroviral Treatment (START) trial.
HIV Med. 2015;16:46-54.

Pool E.R.M., A., Winston, E., Bagkeris, J.H., Vera, P.W.G., Mallon, M., Sachikonye, et al.
High-risk behaviours, and their associations with mental health, adherence to antiretroviral therapy and HIV parameters, in HIV-positive men who have sex with men.
HIV Med. 2019;20(2):131-6.

Besutti G., P., Raggi, S., Zona, R., Scaglioni, A., Santoro, G., Orlando, et al.
Independent association of subclinical coronary artery disease and emphysema in HIV-infected patients.
HIV Med. 2016;17(3):178-87.

Sampériz G., D., Guerrero, M., López, J., Valera, A., Iglesias, A., Ríos, et al.
Prevalence of and risk factors for pulmonary abnormalities in HIV-infected patients treated with antiretroviral therapy.
HIV Med. 2014;15(6):321-9.

Krentz H.B., G., Kliewer, M.J., Gill.
Changing mortality rates and causes of death for HIV-infected individuals living in Southern Alberta, Canada from 1984 to 2003.
HIV Med. 2005;6(2):99-106.

Brown J., J.A., McGowan, H., Chouial, S., Capocci, C., Smith, D., Ivens, et al.
Respiratory health status is impaired in UK HIV-positive adults with virologically suppressed HIV infection.
HIV Med. 2017;18(8):604-12.

Mccombe J., P., Vivithanaporn, M., Gill, C., Power.
Predictors of symptomatic HIV-associated neurocognitive disorders in universal health care.
HIV Med. 2013;14(2):99-107.

Petoumenos K., S., Worm, P., Reiss, S., de Wit, A., d'Arminio Monforte, C., Sabin, et al.
Rates of cardiovascular disease following smoking cessation in patients with HIV infection: Results from the D:A:D study.
HIV Med. 2011;12(7):412-21.

Rasmussen L.H., A., Knudsen, T.L., Katzenstein, J., Gerstoft, N., Obel, N.R., Jørgensen, et al.
Soluble urokinase plasminogen activator receptor (suPAR) is a novel, independent predictive marker of myocardial infarction in HIV-1-infected patients: A nested case-control study.

HIV Med. 2016;17(5):350-7.

Fabbiani M., N., Ciccarelli, M., Tana, S., Farina, E., Baldonero, V., Di Cristo, et al.
Cardiovascular risk factors and carotid intima-media thickness are associated with lower cognitive performance in HIV-infected patients.
HIV Med. 2013;14(3):136-44.

Palacios Muñoz R., J., Santos, A., García, E., Castells, M., González, J., Ruiz, et al.
Impact of highly active antiretroviral therapy on blood pressure in HIV-infected patients. A prospective study in a cohort of naive patients.
HIV Med. 2006;7(1):10-5.

Thudium R.F., J., Lundgren, T., Benfield, B.G., Nordestgaard, Á.H., Borges, J., Gerstoft, et al.
HIV infection is independently associated with a higher concentration of alpha-1 antitrypsin.
HIV Med. 2018;19(10):745-50.

Serrano-Villar S., S., Moreno, M., Fuentes-Ferrer, C., Sánchez-Marcos, M., Ávila, T., Sainz, et al.
The CD4: CD8 ratio is associated with markers of age-associated disease in virally suppressed HIV-infected patients with immunological recovery.
HIV Med. 2014;15(1):40-9.

Do T.C., D., Boettiger, M., Law, S., Pujari, F., Zhang, R., Chaiwarith, et al.
Smoking and projected cardiovascular risk in an HIV-positive Asian regional cohort.
HIV Med. 2016;17(7):542-9.

Glass T.R., C., Ungsedhapand, M., Wolbers, R., Weber, P.L., Vernazza, M., Rickenbach, et al.
Prevalence of risk factors for cardiovascular disease in HIV-infected patients over time: The Swiss HIV Cohort Study.
HIV Med. 2006;7(6):404-10.

Iloeje U.H., Y., Yuan, G., L'Italien, J., Mauskopf, S.D., Holmberg, A.C., Moorman, et al.
Protease inhibitor exposure and increased risk of cardiovascular disease in HIV-infected patients.
HIV Med. 2005;6(1):37-44.

Yuan Y., G., L'Italien, J., Mukherjee, U.H., Iloeje.
Determinants of discontinuation of initial highly active antiretroviral therapy regimens in a US HIV-infected patient cohort.
HIV Med. 2006;7(3):156-62.

Horizon A.A., R.J., Joseph, Q., Liao, S.T., Ross, G.E., Pakes.
Characteristics of foot fractures in HIV-infected patients previously treated with tenofovir versus non-tenofovir-containing highly active antiretroviral therapy.
HIV/AIDS - Res Palliative Care. 2011;3:53-9.

Ketut Agus Somia I., T.P., Merati, I., Made Bakta, I.B.P., Manuaba, W.P.S., Yasa, I., Dewa Made Sukrama, et al.
High levels of serum IL-6 and serum hepcidin and low CD4 cell count were risk factors of anemia of chronic disease in HIV patients on the combination of antiretroviral therapy.
HIV/AIDS - Res Palliative Care. 2019;11:133-9.

Ayalew M.B., D., Kumilachew, A., Belay, S., Getu, D., Teju, D., Endale, et al.
First-line antiretroviral treatment failure and associated factors in HIV patients at the university of Gondar teaching hospital, Gondar, Northwest Ethiopia.
HIV/AIDS - Res Palliative Care. 2016;8:141-6.

Troy S.B., A.E.B., Rossheim, J., Siik, T.D., Cunningham, J.A., Kerry.
Association of CMV, HBV, or HCV co-infection with vaccine response in adults with well-controlled HIV infection.
Hum Vaccines Immunother. 2016;12(5):1295-9.

Rocha-Brischiliari S.C., F., Gimenes, A.L.P., De Abreu, M.M.T., Irie, R.P., Souza, R.G., Santana, et al.

Risk factors for cervical HPV infection and genotypes distribution in HIV-infected South Brazilian women.
Infect Agents Cancer. 2014;9(1).

Knudsen A., C.A.E., Malmberg, A., KjÆr, A.-M., Lebech.
Low prevalence of peripheral arterial disease in a cross-sectional study of Danish HIV-infected patients.
Infect Dis. 2015;47(11):776-82.

Aaron E., A., Bonacquisti, L., Mathew, G., Alleyne, L.P., Bamford, J.F., Culhane.
Small-for-gestational-age births in pregnant women with HIV, due to severity of HIV disease, not antiretroviral therapy.
Infect Dis Obstet Gynecol. 2012;2012.

Nduka C.U., O.A., Uthman, P.K., Kimani, A.O., Malu, S., Stranges.
Impact of body fat changes in mediating the effects of antiretroviral therapy on blood pressure in HIV-infected persons in a sub-Saharan African setting.
Infect Dis Pover. 2016;5(1).

Badie S.M., M., Rasoulinejad, M.R., Salehi, H.E., Kochak, S.A.S., Alinaghi, S.A.D., Manshadi, et al.
Evaluation of echocardiographic abnormalities in HIV positive patients treated with antiretroviral medications.
Infect Disord Drug Targets. 2017;17(1):43-51.

Madeddu G., A.G., Fois, G.M., Calia, S., Babudieri, V., Soddu, F., Becciu, et al.
Chronic obstructive pulmonary disease: An emerging comorbidity in HIV-infected patients in the HAART era?
Infection. 2013;41(2):347-53.

Madeddu G., E.M., Porqueddu, F., Cambosu, F., Saba, A.G., Fois, P., Pirina, et al.
Bacterial community acquired pneumonia in HIV-infected inpatients in the highly active antiretroviral therapy era.
Infection. 2008;36(3):231-6.

Aboud M., A., Elgalib, L., Pomeroy, G., Panayiotakopoulos, E., Skopelitis, R., Kulasegaram, et al.
Cardiovascular risk evaluation and antiretroviral therapy effects in an HIV cohort: Implications for clinical management: The CREATE 1 study.
Int J Clin Pract. 2010;64(9):1252-9.

May M., J.A.C., Sterne, M., Shipley, E., Brunner, R., D'Agostino, P., Whincup, et al.
A coronary heart disease risk model for predicting the effect of potent antiretroviral therapy in HIV-1 infected men.
Int J Epidemiol. 2007;36(6):1309-18.

Ndona M.M., B., Longo-Mbenza, R., Wumba, B.T., Umba, B., Buassa-bu-Tsumbu, M.M., Mambimbi, et al.
Nadir CD4+, religion, antiretroviral therapy, incidence of type 2 diabetes mellitus, and increasing rates of obesity among black Africans with HIV disease.
Int J Gen Med. 2012;5:983-90.

Florindo A.A., M.D.R.D.D.O., Latorre, P.C., Jaime, A.A.C., Segurado.
Leisure time physical activity prevents accumulation of central fat in HIV/AIDS subjects on highly active antiretroviral therapy.
Int J STD AIDS. 2007;18(10):692-6.

Short C.-E.S., S.G., Shaw, M.J., Fisher, K., Walker-Bone, Y.C., Gilleece.
Prevalence of and risk factors for osteoporosis and fracture among a male HIV-infected population in the UK.
Int J STD AIDS. 2014;25(2):113-21.

Veld D.H., S., Pengpid, R., Colebunders, L., Skaal, K., Peltzer.
High-risk alcohol use and associated socio-demographic, health and psychosocial factors in patients with HIV infection in three primary health care clinics in South Africa.
Int J STD AIDS. 2017;28(7):651-9.

Quiros-Roldan E., C., Torti, C., Tinelli, F., Moretti, B., Zanini, V., Tirelli, et al.
Risk factors for myocardial infarction in HIV-positive patients.

Int J STD AIDS. 2005;16(1):14-8.

Li J., H., Lai, S., Chen, S., Lai.
Impact of cocaine use on protease inhibitor-associated dyslipidemia in HIV-infected adults.
Int J STD AIDS. 2018;29(8):781-9.

Jordan M.R., Y., Obeng-Aduasare, H., Sheehan, S.Y., Hong, N., Terrin, D.V., Duong, et al.
Correlates of non-adherence to antiretroviral therapy in a cohort of HIV-positive drug users receiving antiretroviral therapy in Hanoi, Vietnam.
Int J STD AIDS. 2014;25(9):662-8.

Shin S.S., C.L., Carpenter, M.L., Ekstrand, Q., Wang, S., Grover, N.M., Zetola, et al.
Cervical cancer awareness and presence of abnormal cytology among HIV-infected women on antiretroviral therapy in rural Andhra Pradesh, India.
Int J STD AIDS. 2019;30(6):586-95.

Deshwal R., S., Arora.
High Prevalence of Vitamin D Deficiency in HIV Infected on Antiretroviral Therapy in a Cohort of Indian Patients.
J Assoc Physicians India. 2019;67(3):42-5.

Balasundaram A., S., Sarkar, A., Hamide, S., Lakshminarayanan.
Socioepidemiologic profile and treatment-seeking behaviour of HIV/AIDS patients in a tertiary-care hospital in south India.
J Health Popul Nutr. 2014;32(4):587-94.

Morojele N.K., S., Nkosi, C.T., Kekwaletswe, P.A., Shuper, S.O., Manda, B., Myers, et al.
Utility of Brief Versions of the Alcohol Use Disorders Identification Test (AUDIT) to Identify Excessive Drinking Among Patients in HIV Care in South Africa.
J Stud Alcohol Drugs. 2017;78(1):88-96.

Muiru A.N., P., Bibangambah, L., Hemphill, R., Sentongo, J.-H., Kim, V.A., Triant, et al.
Distribution and Performance of Cardiovascular Risk Scores in a Mixed Population of HIV-Infected and Community-Based HIV-Uninfected Individuals in Uganda.
J Acquir Immune Defic Syndr. 2018;78(4):458-64.

Farhadian S., E., Jalbert, Y., Deng, M.B., Goetz, L.S., Park, A.C., Justice, et al.
HIV and Age Do Not Synergistically Affect Age-Related T-Cell Markers.
J Acquir Immune Defic Syndr. 2018;77(3):337-44.

Mussini C., L., Galli, A.C., Lepri, A., De Luca, A., Antinori, R., Libertone, et al.
Incidence, timing, and determinants of bacterial pneumonia among HIV-infected patients: Data from the ICONA foundation cohort.
J Acquired Immune Defic Syndr. 2013;63(3):339-45.

Jacobson D.L., D., Spiegelman, T.K., Knox, I.B., Wilson.
Evolution and predictors of change in total bone mineral density over time in HIV-infected men and women in the nutrition for healthy living study.
J Acquired Immune Defic Syndr. 2008;49(3):298-308.

MacDonald D.M., A.C., Melzer, G., Collins, A., Avihingsanon, K., Crothers, N.E., Ingraham, et al.
Smoking and accelerated lung function decline in HIV-positive individuals: A secondary analysis of the START pulmonary substudy.
J Acquired Immune Defic Syndr. 2018;79(3):E85-E92.

Drozd D.R., M.M., Kitahata, K.N., Althoff, J., Zhang, S.J., Gange, S., Napravnik, et al.
Increased Risk of Myocardial Infarction in HIV-Infected Individuals in North America Compared with the General Population.
J Acquired Immune Defic Syndr. 2017;75(5):568-76.

Volpe G.E., A.M., Tang, J.F., Polak, A., Mangili, S.C., Skinner, C.A., Wanke.

Progression of carotid intima-media thickness and coronary artery calcium over 6 years in an HIV-infected cohort.
J Acquired Immune Defic Syndr. 2013;64(1):51-7.

Haskelberg H., P.W.G., Mallon, J., Hoy, J., Amin, C., Moore, P., Phanuphak, et al.
Bone mineral density over 96 weeks in adults failing first-line therapy randomized to raltegravir/lopinavir/ritonavir compared with standard second-line therapy.
J Acquired Immune Defic Syndr. 2014;67(2):161-8.

Bucher H.C., W., Richter, T.R., Glass, L., Magenta, Q., Wang, M., Cavassini, et al.
Small dense lipoproteins, apolipoprotein B, and risk of coronary events in HIV-infected patients on antiretroviral therapy: The Swiss HIV Cohort Study.
J Acquired Immune Defic Syndr. 2012;60(2):135-42.

Leader J.K., K., Crothers, L., Huang, M.A., King, A., Morris, B.W., Thompson, et al.
Risk factors associated with quantitative evidence of lung emphysema and fibrosis in an HIV-infected cohort.
J Acquired Immune Defic Syndr. 2016;71(4):420-7.

Anastos K., M.F., Schneider, S.J., Gange, H., Minkoff, R.M., Greenblatt, J., Feldman, et al.
The association of race, sociodemographic, and behavioral characteristics with response to highly active antiretroviral therapy in women.
J Acquired Immune Defic Syndr. 2005;39(5):537-44.

Erlandson K.M., A.A., Allshouse, C.M., Jankowski, S., Duong, S., Mawhinney, W.M., Kohrt, et al.
Risk factors for falls in HIV-infected persons.
J Acquired Immune Defic Syndr. 2012;61(4):484-9.

Cockerham L., R., Scherzer, A., Zolopa, D., Rimland, C.E., Lewis, P., Bacchetti, et al.
Association of HIV infection, demographic and cardiovascular risk factors with all-cause mortality in the recent haart Era.
J Acquired Immune Defic Syndr. 2010;53(1):102-6.

Reid M.J.A., S.M., Baxi, L.A., Sheira, A.L., Landay, E.A., Frongillo, A., Adedimeji, et al.
Higher Body Mass Index Is Associated with Greater Proportions of Effector CD8+ T Cells Expressing CD57 in Women Living with HIV.
J Acquired Immune Defic Syndr. 2017;75(5):e132-e41.

Echeverría P., A., Bonjoch, J., Moltó, A., Jou, J., Puig, A., Ornelas, et al.
Pulse wave velocity as index of arterial stiffness in hiv-infected patients compared with a healthy population.
J Acquired Immune Defic Syndr. 2014;65(1):50-6.

Allavena C., C., Delpierre, L., Cuzin, D., Rey, N., Viget, J., Bernard, et al.
High frequency of vitamin D deficiency in HIV-infected patients: Effects of HIV-related factors and antiretroviral drugs.
J Antimicrob Chemother. 2012;67(9):2222-30.

Yanagisawa N., M., Ando, K., Tsuchiya, K., Nitta.
HIV-infected men with an elevated level of serum cystatin C have a high likelihood of developing cancers.
J Antivirals Antiretrovirals. 2012;4(2):0038-42.

Salyer J., D.E., Lyon, J., Settle, R.K., Elswick, D., Rackley.
Coronary heart disease risks and lifestyle behaviors in persons with HIV infection.
J Assoc Nurses AIDS Care. 2006;17(3):3-17.

Mandas A., E.L., Iorio, M.G., Congiu, C., Balestrieri, A., Mereu, D., Cau, et al.
Oxidative imbalance in HIV-1 infected patients treated with antiretroviral therapy.
J Biomed Biotechnol. 2009;2009.

Sarfo F.S., M., Nichols, A., Singh, Y., Hardy, B., Norman, G., Mensah, et al.
Characteristics of hypertension among people living with HIV in Ghana: Impact of new hypertension guideline.
J Clin Hypertens. 2019;21(6):838-50.

Freitas P., D., Carvalho, A.C., Santos, A.J., Madureira, S., Xerinda, E., Martinez, et al.
Central/peripheral fat mass ratio is associated with increased risk of hypertension in hiv-infected patients.
J Clin Hypertens. 2012;14(9):593-600.

Payam A., B., Shiramizu, C., Shikuma, C., Milne, K., Terada, E., Kajioka, et al.
Hiv-associated anal dysplasia: Experience from a multiethnic-HIV clinic in Hawaii.
J Health Care Poor Underserved. 2011;22(4):16-22.

Pastori D., A., Esposito, R., Carnevale, S., Bartimoccia, M., Novo, A., Fantauzzi, et al.
HIV-1 induces invivo platelet activation by enhancing platelet NOX2 activity.
J Infect. 2015;70(6):651-8.

Guzmán-Fulgencio M., J., Medrano, N., Rallón, A., Echeverria-Urabayen, J., Miguel Benito, C., Restrepo, et al.
Soluble markers of inflammation are associated with Framingham scores in HIV-infected patients on suppressive antiretroviral therapy.
J Infect. 2011;63(5):382-90.

Nakamura H., M., Tateyama, D., Tasato, S., Haranaga, T., Ishimine, F., Higa, et al.
The prevalence of airway obstruction among Japanese HIV-positive male patients compared with general population; A case-control study of single center analysis.
J Infect Chemother. 2014;20(6):361-4.

Ronit A., S., Sharma, J.V., Baker, R., Mngqibisa, T., Delory, L., Caldeira, et al.
Serum Albumin as a Prognostic Marker for Serious Non-AIDS Endpoints in the Strategic Timing of Antiretroviral Treatment (START) Study.
J Infect Dis. 2018;217(3):405-12.

Parrinello C.M., E., Sinclair, A.L., Landay, N., Lurain, A.R., Sharrett, S.J., Gange, et al.
Cytomegalovirus immunoglobulin G antibody is associated with subclinical carotid artery disease among HIV-infected women.
J Infect Dis. 2012;205(12):1788-96.

Siedner M.J., J.-H., Kim, R.S., Nakku, P., Bibangambah, L., Hemphill, V.A., Triant, et al.
Persistent immune activation and carotid atherosclerosis in HIV-infected ugandans receiving antiretroviral therapy.
J Infect Dis. 2016;213(3):370-8.

Haberer J.E., B.M., Bwana, C., Orrell, S., Asiimwe, G., Amanyire, N., Musinguzi, et al.
ART adherence and viral suppression are high among most non-pregnant individuals with early-stage, asymptomatic HIV infection: an observational study from Uganda and South Africa.
J Int AIDS Soc. 2019;22(2).

Nouaman M.N., M., Vinikoor, M., Seydi, D.K., Ekouevi, P.A., Coffie, L., Mulenga, et al.
High prevalence of binge drinking among people living with HIV in four African countries.
J Int AIDS Soc. 2018;21(12).

Helou E., S., Shenoi, T., Kyriakides, M.-L., Landry, M., Kozal, L.A., Barakat.
Characterizing Patients with Very-Low-Level HIV Viremia: A Community-Based Study.
J Int Assoc Providers AIDS Care. 2017;16(3):261-6.

Poudel K.C., P.H., Palmer, M., Jimba, T., Mizoue, J., Kobayashi, K., Poudel-Tandukar.
Coinfection with hepatitis C virus among HIV-positive people in the Kathmandu Valley, Nepal.
J Int Assoc Providers AIDS Care. 2014;13(3):277-83.

Aderemi-Williams R.I., F., Tayo, A., Sagoe, M.P., Zachariah.
Effect of 2 Models of Care and Factors Predicting Patients' Adherence to Doctor's Appointment Attendance in Lagos State University Teaching Hospital, Ikeja, Lagos, Nigeria.
J Int Assoc Providers AIDS Care. 2017;16(3):296-302.

Wu P.-Y., M.-Y., Chen, W.-H., Sheng, S.-M., Hsieh, Y.-C., Chuang, A., Cheng, et al.
Estimated risk of cardiovascular disease among the HIV-positive patients aged 40 years or older in Taiwan.
J Microbiol Immunol Infect. 2019;52(4):549-55.

Heaps-Woodruff J.M., P.W., Wright, B.M., Ances, D., Clifford, R.H., Paul.
The impact of human immune deficiency virus and hepatitis C coinfection on white matter microstructural integrity.
J Neurovirol. 2016;22(3):389-99.

Kaio D.J., P.H.C., Rondó, J.M.P., Souza, A.V., Firmino, L.A., Luzia, A.A., Segurado.
Vitamin A and beta-carotene concentrations in adults with HIV/AIDS on highly active antiretroviral therapy.
J Nutr Sci Vitaminol. 2013;59(6):496-502.

Jerome C.S., M., Agonnoudé, G.E., Sopoh, A.I., Bah-Chabi, A., de Souza, M., Bachabi, et al.
Sociodemographic, lifestyle and therapeutic predictors of 2-year survival in HIV-infected persons receiving antiretroviral therapy in Benin.
J Public Health Afr. 2017;8(1):60-4.

Guaraldi G., M., Beggi, S., Zona, K., Luzi, G., Orlando, F., Carli, et al.
Erectile Dysfunction Is Not a Mirror of Endothelial Dysfunction in HIV-Infected Patients.
J Sex Med. 2012;9(4):1114-21.

Makinson A., J.-C., Tenon, S., Eymard-Duvernay, J.-L., Pujol, C., Allavena, L., Cuzin, et al.
Human immunodeficiency virus infection and non-small cell lung cancer: Survival and toxicity of antineoplastic chemotherapy in a cohort study.
J Thorac Oncol. 2011;6(6):1022-9.

Muramatsu T., K., Amano, Y., Chikasawa, M., Bingo, M., Yotsumoto, M., Otaki, et al.
Chronic kidney disease is related to femoral neck bone loss among HIV-1-infected patients: A retrospective study.
J Tokyo Med Univ. 2019;77(1):11-22.

Baranoski A.S., A., Harris, D., Michaels, R., Miciek, T., Storer, P., Sebastiani, et al.
Relationship between poor physical function, inflammatory markers, and Comorbidities in HIV-infected women on antiretroviral therapy.
J Women's Health. 2014;23(1):69-76.

Sharma S., P., Khadga, G.P., Dhungana, U., Chitrakar.
Medication adherence to antiretroviral therapy among patients visiting antiretroviral therapy center at Tribhuvan university teaching hospital, Kathmandu, Nepal.
Kathmandu Univ Med J. 2013;11(41):50-3.

Sabin C.A., S.W., Worm, R., Weber, P., Reiss, W., El-Sadr, F., Dabis, et al.
Use of nucleoside reverse transcriptase inhibitors and risk of myocardial infarction in HIV-infected patients enrolled in the D:A:D study: A multi-cohort collaboration.
Lancet. 2008;371(9622):1417-26.

Ryom L., J.D., Lundgren, W., El-Sadr, P., Reiss, O., Kirk, M., Law, et al.
Cardiovascular disease and use of contemporary protease inhibitors: the D:A:D international prospective multicohort study.
Lancet HIV. 2018;5(6):e291-e300.

North C.M., J.G., Allen, S., Okello, R., Sentongo, B., Kakuhikire, E.T., Ryan, et al.
HIV Infection, Pulmonary Tuberculosis, and COPD in Rural Uganda: A Cross-Sectional Study.
Lung. 2018;196(1):49-57.

Hulgan T., M.S., Boger, D.H., Liao, G.A., McComsey, C.A., Wanke, A., Mangili, et al.
Urinary eicosanoid metabolites in HIV-infected women with central obesity switching to raltegravir: An analysis from the women, integrase, and fat accumulation trial.
Mediators Inflamm. 2014;2014.

Makhubele T.G., H.C., Steel, R., Anderson, G., Van Dyk, A.J., Theron, T.M., Rossouw.
Systemic Immune Activation Profiles of HIV-1 Subtype C-Infected Children and Their Mothers.
Mediators Inflamm. 2016;2016.

Knudsen A., T.E., Christensen, A.A., Ghotbi, P., Hasbak, A.-M., Lebech, A., Kjær, et al.
Normal myocardial flow reserve in HIV-infected patients on stable antiretroviral therapy: A cross-sectional study using rubidium-82 PET/CT.
Medicine. 2015;94(43).

Obry-Roguet V., S., Brégigeon, C.E., Cano, C., Lions, O., Zaegel-Faucher, H., Laroche, et al.
Risk factors associated with overweight and obesity in HIV-infected people: Aging, behavioral factors but not cART in a cross-sectional study.
Medicine. 2018;97(23).

Looby S.E., C., Psaros, G., Raggio, C., Rivard, L., Smeaton, J., Shifren, et al.
Association between HIV status and psychological symptoms in perimenopausal women.
Menopause. 2018;25(6):648-56.

Gonçalves R.M.C., R.A., Mendoza-Sassi, M.S., Graudenz.
Changes to the prevalence of cervical squamous intraepithelial lesions and risk factors among HIV/AIDS patients attended at a specialized service in southern Brazil from 1995 to 1999 and 2006 to 2007.
Modificações na prevalência das lesões intra-epiteliais escamosas cervicais e dos fatores de risco em pacientes HIV/AIDS atendidas em serviço especializado no sul do Brasil nos períodos 1995-1999 e 2006-2007. 2009;42(1):33-8.

Kakar S., D., Drak, T., Amin, J., Cheung, C.C., O'Connor, D.M., Gracey.
Screening and management practices for renal disease in the HIV-positive patient population of an inner metropolitan sexual health service.
Nephrology. 2017;22(2):174-8.

Shuter J., S., Bernstein.
Cigarette smoking is an independent predictor of nonadherence in HIV-infected individuals receiving highly active antiretroviral therapy.
Nicotine Tob Res. 2008;10(4):731-6.

Edward A.O., A.A., Oladayo, A.S., Omolola, A.A., Adetiloye, P.A., Adedayo.
Prevalence of traditional cardiovascular risk factors and evaluation of cardiovascular risk using three risk equations in Nigerians living with human immunodeficiency virus.
North Am J Med Sci. 2013;5(12):680-8.

Krishnan S., J.T., Schouten, D.L., Jacobson, C.A., Benson, A.C., Collier, S.L., Koletar, et al.
Incidence of Non-AIDS-defining cancer in antiretroviral treatment-naïve subjects after antiretroviral treatment initiation: An ACTG longitudinal linked randomized trials analysis.
Oncology (Switzerland). 2011;80(1):42-9.

Melaku T., G., Mamo, L., Chelkeba, T., Chanie.
Immunologic restoration of people living with human immunodeficiency virus on highly active anti-retroviral therapy in Ethiopia: The focus of chronic non-communicable disease co-morbidities.
Open AIDS J. 2019;13(1):36-48.

Lake J.E., R.M., Hoffman, C.-H., Tseng, H.M., Wilhalme, J.S., Adams, J.S., Currier.
Success of standard dose vitamin D supplementation in treated human immunodeficiency virus infection.
Open Forum Infect Dis. 2015;2(2).

Baker J.V., K.H., Hullsiek, N.W., Engen, R., Nelson, P., Chetchotisakd, J., Gerstoft, et al.
Early antiretroviral therapy at high CD4 counts does not improve arterial elasticity: A substudy of the strategic timing of antiretroviral treatment (START) trial.
Open Forum Infect Dis. 2016;3(4).

Kulkarni M., E., Bowman, J., Gabriel, T., Amburgy, E., Mayne, D.A., Zidar, et al.

Altered monocyte and endothelial cell adhesion molecule expression is linked to vascular inflammation in human immunodeficiency virus infection.
Open Forum Infect Dis. 2016;3(4).

Dirajlal-Fargo S., M., Kulkarni, E., Bowman, L., Shan, A., Sattar, N., Funderburg, et al.
Serum albumin is associated with higher inflammation and carotid atherosclerosis in treated human immunodeficiency virus infection.
Open Forum Infect Dis. 2018;5(11).

Alencherry B., G., Erem, G., Mirembe, I., Ssinabulya, C.-H., Yun, C.-L., Hung, et al.
Coronary artery calcium, HIV and inflammation in Uganda compared with the USA.
Open Heart. 2019;6(1).

Mama M., A., Manilal, H., Tesfa, H., Mohammed, E., Erbo.
Prevalence of pulmonary tuberculosis and associated factors among HIV positive patients attending antiretroviral therapy clinic at Arba minch general hospital, southern Ethiopia.
Open Microbiol J. 2018;12:163-71.

Nittayananta W., K., Amornthatree, M., Kemapunmanus, S., Talungchit, H., Sriplung.
Expression of oral cytokines in HIV-infected subjects with long-term use of antiretroviral therapy.
Oral Dis. 2014;20(3):e57-e64.

Engeland C.G., P., Jang, M., Alves, P.T., Marucha, J., Califano.
HIV infection and tooth loss.
Oral Surg Oral Med Oral Pathol Oral Radiol Endod. 2008;105(3):321-6.

Kazooba P., I., Kasamba, B.N., Mayanja, J., Lutaakome, I., Namakoola, T., Salome, et al.
Cardiometabolic risk among HIV-positive Ugandan adults: Prevalence, predictors and effect of long-term antiretroviral therapy.
Pan Afr Med J. 2017;27.

Mai H.T., G.M., Le, B.X., Tran, H.N., Do, C.A., Latkin, L.T., Nguyen, et al.
Adherence to antiretroviral therapy among HIV/ AIDS patients in the context of early treatment initiation in Vietnam.
Patient Preference Adherence. 2018;12:2131-7.

Da Silva É.F.R., K.C., Bassichetto, D.S., Lewi.
Lipid profile, cardiovascular risk factors and metabolic syndrome in a group of AIDS patients.
Perfil lipídico, fatores de risco cardiovascular e síndrome metabólica em um grupo de pacientes com AIDS. 2009;93(2):113-8.

Nguyen S.T., S.A., Eaton, A.M., Bain, A.P., Rahman, K.D., Payne, R., Bedimo, et al.
Lipid-lowering efficacy and safety after switching to atazanavir-ritonavir- based highly active antiretroviral therapy in patients with human immunodeficiency virus.
Pharmacotherapy. 2008;28(3):323-30.

Amberbir A., V., Banda, V., Singano, A., Matengeni, C., Pfaff, Z., Ismail, et al.
Effect of cardio-metabolic risk factors on all-cause mortality among HIV patients on antiretroviral therapy in Malawi: A prospective cohort study.
PLoS ONE. 2019;14(1).

Castley A., L., Williams, I., James, G., Guelfi, C., Berry, D., Nolan.
Plasma CXCL10, sCD163 and sCD14 levels have distinct associations with antiretroviral treatment and cardiovascular disease risk factors.
PLoS ONE. 2016;11(6).

Cournil A., S., Eymard-Duvernay, A., Diouf, C., Moquet, J., Coutherut, N.F.N., Gueye, et al.
Reduced quantitative ultrasound bone mineral density in HIV-infected patients on antiretroviral therapy in Senegal.
PLoS ONE. 2012;7(2).

Martin A., C.L., Moore, P.W., Mallon, J.F., Hoy, S., Emery, W.H., Belloso, et al.
HIV lipodystrophy in participants randomised to lopinavir/ritonavir (LPV/r) +2-3 nucleoside/nucleotide reverse transcriptase inhibitors (N(t)RTI) or LPV/r + raltegravir as second-line antiretroviral therapy.
PLoS ONE. 2013;8(10).

Petraglia A., J.K., Leader, M., Gingo, M., Fitzpatrick, J., Ries, C., Kessinger, et al.
Emphysema is associated with thoracic vertebral bone attenuation on chest CT scan in HIV-infected individuals.
PLoS ONE. 2017;12(4).

Winston A., A., Arenas-Pinto, W., Stöhr, M., Fisher, C.M., Orkin, K., Aderogba, et al.
Neurocognitive Function in HIV Infected Patients on Antiretroviral Therapy.
PLoS ONE. 2013;8(4).

Hatcher A.M., A.C., Tsai, E., Kumbakumba, S.L., Dworkin, P.W., Hunt, J.N., Martin, et al.
Sexual Relationship Power and Depression among HIV-Infected Women in Rural Uganda.
PLoS ONE. 2012;7(12).

Ghehi C., D., Gabillard, R., Moh, A., Badje, G.M., Kouamé, E., Oouttara, et al.
High correlation between Framingham equations with BMI and with lipids to estimate cardiovascular risks score at baseline in HIV-infected adults in the Temprano trial, ANRS 12136 in Côte d'Ivoire.
PLoS ONE. 2017;12(6).

Dimala C.A., J., Atashili, J.C., Mbuagbaw, A., Wilfred, G.L., Monekosso.
A comparison of the diabetes risk score in HIV/AIDS patients on Highly Active Antiretroviral Therapy (HAART) and HAART-naïve patients at the Limbe Regional Hospital, Cameroon.
PLoS ONE. 2016;11(5).

Miller C.J., J.V., Baker, A.M., Bormann, K.M., Erlandson, K.H., Hullsiek, A.C., Justice, et al.
Adjudicated morbidity and mortality outcomes by age among individuals with HIV infection on suppressive antiretroviral therapy.
PLoS ONE. 2014;9(4).

Guaraldi G., G., Besutti, R., Scaglioni, A., Santoro, S., Zona, L., Guido, et al.
The Burden of image based emphysema and bronchiolitis in HIV-infected individuals on antiretroviral therapy.
PLoS ONE. 2014;9(10).

Bultum J.A., N., Yigzaw, W., Demeke, M., Alemayehu.
Alcohol use disorder and associated factors among human immunodeficiency virus infected patients attending antiretroviral therapy clinic at Bishoftu General Hospital, Oromiya region, Ethiopia.
PLoS ONE. 2018;13(3).

Liu J.C.Y., J.M., Leung, D.A., Ngan, N.F., Nashta, S., Guillemi, M., Harris, et al.
Absolute leukocyte telomere length in HIV-infected and uninfected individuals: Evidence of accelerated cell senescence in HIV-associated chronic obstructive pulmonary disease.
PLoS ONE. 2015;10(4).

Lake J.E., C.-H., Tseng, J.S., Currier.
A Pilot Study of Telmisartan for Visceral Adiposity in HIV Infection: The Metabolic Abnormalities, Telmisartan, and HIV Infection (MATH) Trial.
PLoS ONE. 2013;8(3).

Baker J.V., K., Huppler Hullsiek, R., Prosser, D., Duprez, R., Grimm, R.P., Tracy, et al.
Angiotensin Converting Enzyme Inhibitor and HMG-CoA Reductase Inhibitor as Adjunct Treatment for Persons with HIV Infection: A Feasibility Randomized Trial.
PLoS ONE. 2012;7(10).

Barska K., W., Kwiatkowska, B., Knysz, K., Arczyńska, M., Karczewski, W., Witkiewicz.
The role of the tissue factor and its inhibitor in the development of subclinical atherosclerosis in people living with HIV.

PLoS ONE. 2017;12(7).

Rollet-Kurhajec K.C., E.E.M., Moodie, S., Walmsley, C., Cooper, N., Pick, M.B., Klein.
Hepatic fibrosis progression in HIV-hepatitis C virus co-infection - The effect of sex on risk of significant fibrosis measured by aspartate-to-platelet ratio index.
PLoS ONE. 2015;10(6).

Furuya-Kanamori L., M.D., Kelly, S.J., McKenzie.
Co-Morbidity, Ageing and Predicted Mortality in Antiretroviral Treated Australian Men: A Quantitative Analysis.
PLoS ONE. 2013;8(10).

Rabkin M., A., Mutiti, C., Chung, Y., Zhang, Y., Wei, W.M., El-Sadr.
Missed opportunities to address cardiovascular disease risk factors amongst adults attending an urban HIV clinic in South Africa.
PLoS ONE. 2015;10(10).

George M.P., M., Kannass, L., Huang, F.C., Sciurba, A., Morris.
Respiratory symptoms and airway obstruction in HIV-infected subjects in the HAART era.
PLoS ONE. 2009;4(7).

Gingo M.R., Y., Zhang, K.B., Ghebrehawariat, J.-H., Jeong, Y., Chu, Q., Yang, et al.
Elevated NT-pro-brain natriuretic peptide level is independently associated with all-cause mortality in HIV-infected women in the early and recent HAART eras in the women's interagency HIV study cohort.
PLoS ONE. 2015;10(3).

Weldehaweria N.B., E.H., Abreha, M.G., Weldu, K.H., Misgina.
Psychosocial correlates of nutritional status among people living with HIV on antiretroviral therapy: A matched case-control study in Central zone of Tigray, Northern Ethiopia.
PLoS ONE. 2017;12(3).

Kristoffersen U.S., A.-M., Lebech, N., Wiinberg, C.L., Petersen, P., Hasbak, H., Gutte, et al.
Silent Ischemic Heart Disease and Pericardial Fat Volume in HIV-Infected Patients: A Case-Control Myocardial Perfusion Scintigraphy Study.
PLoS ONE. 2013;8(8).

Szymanek-Pasternak A., A., Szymczak, M., Zalewska, K., Małyszczak, B., Knysz.
Risk factors for chronic kidney disease do not influence the serum levels of asymmetric dimethylarginine in HIV-1-infected patients without significant renal disease.
Pol Arch Med Wewn. 2016;126(9):672-80.

Fontela C., J., Castilla, R., Juanbeltz, I., Martínez-Baz, M., Rivero, A., O'Leary, et al.
Comorbidities and cardiovascular risk factors in an aged cohort of HIV-infected patients on antiretroviral treatment in a Spanish hospital in 2016.
Postgrad Med. 2018;130(3):317-24.

Turčinov D., J., Begovac.
Predicted coronary heart disease risk in Croatian HIV infected patients treated with combination antiretroviral therapy.
Predvid{stroke}anje rizika koronarne bolesti srca u sudionika zaraženih HIV-om iz Hrvatske liječenih cart-om. 2011;35(1):115-21.

Knobel H., P., Domingo, I., Suarez-Lozano, F., Gutierrez, V., Estrada, R., Palacios, et al.
Rate of cardiovascular, renal and bone disease and their major risks factors in HIV-infected individuals on antiretroviral therapy in Spain.
Prevalencia de enfermedad cardiovascular, renal y ósea, y sus principales factores de riesgo en personas infectadas por el VIH que reciben tratamiento antirretroviral en España. 2019;37(6):373-9.

Siyahhan Julnes P., S., Auh, R., Krakora, K., Withers, D., Nora, L., Matthews, et al.
The Association Between Post-traumatic Stress Disorder and Markers of Inflammation and Immune Activation in HIV-Infected Individuals With Controlled Viremia.

Psychosomatics. 2016;57(4):423-30.

Willig A.L., P.A., Kramer, B.K., Chacko, V.M., Darley-Usmar, S.L., Heath, E.T., Overton.
Monocyte bioenergetic function is associated with body composition in virologically suppressed HIV-infected women.
Redox Biol. 2017;12:648-56.

Passos A.I.M., E.R., Couto, S.M., de Rezende, M.L., Moretti.
Evaluation of functional respiratory parameters in AIDS patients assisted in the infectious diseases ambulatory care clinic of a tertiary care university hospital in Brazil.
Respir Care. 2012;57(4):544-9.

Almeida S.E., M., Borges, M., Fiegenbaum, C.C., Nunes, M.L., Rossetti.
Metabolic changes associated with antiretroviral therapy in HIV-positive patients.
Rev Saude Publica. 2009;43(2):283-90.

Jaime P.C., A.A., Florindo, O., Latorre M. do R.D. de, A.A.C., Segurado.
Central obesity and dietary intake in HIV/AIDS patients.
Rev Saude Publica. 2006;40(4):634-40.

Sansores C.C., A.S., Rivero, A.G., Flores, R.R., Sánchez, R.A., Góngora Biachi, E.B., Caldelas, et al.
Cardiovascular risk in HIV patients.
Riesgo cardiovascular en pacientes con VIH. 2008;24(4):284-8.

Waweru P., R., Anderson, H., Steel, W.D.F., Venter, D., Murdoch, C., Feldman.
The prevalence of smoking and the knowledge of smoking hazards and smoking cessation strategies among HIV-positive patients in Johannesburg, South Africa.
S Afr Med J. 2013;103(11):858-60.

Biraguma J., E., Mutimura, J.M., Frantz.
Health-related quality of life and associated factors in adults living with HIV in Rwanda.
SAHARA J. 2018;15(1):110-20.

Missailidis C., J., Höijer, M., Johansson, L., Ekström, G., Bratt, B., Hejdeman, et al.
Vitamin D status in Well-Controlled Caucasian HIV Patients in Relation to Inflammatory and Metabolic Markers - A Cross-Sectional Cohort Study in Sweden.
Scand J Immunol. 2015;82(1):55-62.

Colón-López V., D., González-Barrios, S.M., De León, G., Girona-Lozada, M., Machin, E., Charneco, et al.
Population-Based Study of Tobacco Use Among People Living With HIV in Puerto Rico.
Subst Use Misuse. 2018;53(3):420-5.

Ma Q., B.S., Zingman, A.E., Luque, M.A., Fischl, B.M., Gripshover, C.S., Venuto, et al.
Therapeutic drug monitoring of protease inhibitors and efavirenz in HIV-infected individuals with active substance-related disorders.
Ther Drug Monit. 2011;33(3):309-14.

D'Ascenzo F., E., Cerrato, D., Appleton, C., Moretti, A., Calcagno, N., Abouzaki, et al.
Prognostic indicators for recurrent thrombotic events in HIV-infected patients with acute coronary syndromes: Use of registry data from 12 sites in Europe, South Africa and the United States.
Thromb Res. 2014;134(3):558-64.

Bhatta D.N., A., Subedi, N., Sharma.
Tobacco smoking and alcohol drinking among HIV infected people using antiretroviral therapy.
Tob Induced Dis. 2018;16.

Adefolalu A.O., Z.Z., Nkosi, S.A.S., Olorunju.
Patterns of adherence to scheduling and dietary instructions among patients on antiretroviral therapy in Pretoria, South Africa.
Trans R Soc Trop Med Hyg. 2014;108(9):582-8.

Adefolalu A.O., Z.Z., Nkosi, S.A.S., Olorunju.
Patterns of adherence to scheduling and dietary instructions among patients on antiretroviral therapy in Pretoria, South Africa.
Trans R Soc Trop Med Hyg. 2014;108(9):582-8.

Katoto P.D.M.C., F., Thienemann, A.N.H., Bulabula, T.M., Esterhuizen, A.B., Murhula, P.P.M., Lunjwire, et al.
Prevalence and risk factors of metabolic syndrome in HIV-infected adults at three urban clinics in a post-conflict setting, eastern Democratic Republic of the Congo.
Trop Med Int Health. 2018;23(7):795-805.

Fuster F., J.I., Vargas, D., Jensen, V., Sarmiento, P., Acuña, F., Peirano, et al.
CD4/CD8 ratio as a predictor of the response to HBV vaccination in HIV-positive patients: A prospective cohort study.
Vaccine. 2016;34(16):1889-95.

García-Lázaro M., A.R., Román, Á.C., Espejo, I., Pérez-Camacho, C.N., Kindelán, J.J.C., Osorio, et al.
Variability in coronary risk assessment in HIV-infected patients.
Variabilidad en la valoración del riesgo coronario en pacientes infectados por el virus de la inmunodeficiencia humana. 2007;129(14):521-4.

Hinojosa C.A., A.E., Nunez-Salgado, J.E., Anaya-Ayala, H., Laparra-Escareno, L.J., Ortiz-Lopez, J.O., Herrera-Caceres, et al.
Prevalence and variables associated with an abnormal ankle-brachial index among patients with human immunodeficiency virus/acquired immunodeficiency syndrome.
Vascular. 2018;26(5):540-6.

Tomažič J., K., Ul, G., Volčanšek, S., Gorenšek, M., Pfeifer, P., Karner, et al.
Prevalence and risk factors for osteopenia/osteoporosis in an HIV-infected male population.
Wien Klin Wochenschr. 2007;119(21):639-46.

Supplementary References 2. Articles included in the meta-of the association between active smoking and adherence to antiretroviral therapy in people living with HIV

Cigarette smoking is an independent predictor of nonadherence in HIV-infected individuals receiving highly active antiretroviral therapy
Shuter J., Bernstein S.
Nicotine and Tobacco Research (2008) 10:4 (731-736).

Determinants of adherence in a cohort of Belgian HIV patients: A pilot study
Degroote S., Vogelaers D., Vermeir P., Mariman A., De Rick A., Van Der Gucht B., Pelgrom J., Van Wanzeele F., Verhofstede C., Vancauwenberghe J., Vandijck D.
Acta Clinica Belgica (2014) 69:2 (111-115).

Factors associated with non-adherence to antiretroviral therapy in adults with AIDS in the first six months of treatment in Salvador, Bahia State, Brazil
Silva J.A., Dourado I., Brito A.M., Silva C.A.
Cadernos de saúde pública (2015) 31:6 (1188-1198).

Rate and predictors of self-chosen drug discontinuations in highly active antiretroviral therapy-treated HIV-positive individuals
Murri R., Guaraldi G., Lupoli P., Crisafulli R., Marcotullio S., Von Schloesser F., Wu A.W.
AIDS Patient Care and STDs (2009) 23:1 (35-39).

Compliance of antiviral therapy and influencing factors in people living with HIV/AIDS in Nanjing
Wei H., Li M., Zhang X., Bu K., Feng Y., Liu X., Shi L., Chen Y., Bai C., Fu G., Huan X., Wang L.
Chinese Journal of Endemiology (2015) 36:7 (672-676).

Medication adherence in HIV-infected smokers: The mediating role of depressive symptoms
Webb M.S., Vanable P.A., Carey M.P., Blair D.C.
AIDS Education and Prevention (2009) 21:SUPPL. 3 (94-105).

Effects of cigarette smoking and nicotine dependence on adherence to antiretroviral therapy among HIV-positive patients in Vietnam
Nguyen N.T.P., Tran B.X., Hwang L.Y., Markham C.M., Swartz M.D., Vidrine J.I., Phan H.T.T., Latkin C.A., Vidrine D.J.
AIDS Care - Psychological and Socio-Medical Aspects of AIDS/HIV (2016) 28:3 (359-364).

Factors associated with optimal pharmacy refill adherence for antiretroviral medications and plasma HIV RNA non-detectability among HIV-positive crack cocaine users: A prospective cohort study
Hayashi K., Wood E., Kerr T., Dong H., Nguyen P., Puskas C.M., Guillemi S., Montaner J.S.G., Milloy M.-J.
BMC Infectious Diseases (2016) 16:1 Article Number: 455.

Cumulative exposure to stimulants and immune function outcomes among HIV-positive and HIV-negative men in the Multicenter AIDS Cohort Study
Shoptaw S., Stall R., Bordon J., Kao U., Cox C., Li X., Ostrow D.G., Plankey M.W.
International Journal of STD and AIDS (2012) 23:8 (576-580).

Medication adherence to antiretroviral therapy among patients visiting antiretroviral therapy center at Tribhuvan university teaching hospital, Kathmandu, Nepal
Sharma S., Khadga P., Dhungana G.P., Chitrakar U.
Kathmandu University Medical Journal (2013) 11:41 (50-53).

Drug use patterns and adherence to treatment among HIV-positive patients: evidence from a large sample of French outpatients (ANRS-EN12-VESPA 2003)
Peretti-Watel P., Spire B., Lert F., Obadia Y.
Drug and Alcohol Dependence (2006) 82:SUPPL. 1 (S71-S79).

The association of race, sociodemographic, and behavioral characteristics with response to highly active antiretroviral therapy in women
Anastos K., Schneider M.F., Gange S.J., Minkoff H., Greenblatt R.M., Feldman J., Levine A., Delapenha R.,

Cohen M.
Journal of Acquired Immune Deficiency Syndromes (2005) 39:5 (537-544).

Adherence to highly active antiretroviral therapies (HAART) in HIV-infected patients: From a predictive to a dynamic approach
Spire B., Duran S., Souville M., Leport C., Raffi F., Moatti J.-P.
Social Science and Medicine (2002) 54:10 (1481-1496).

The Modalities of Nonadherence to Highly Active Antiretroviral Therapy and the Associated Factors Related to Patients' Sociodemographic Characteristics and Their Caregiving Perceptions in Ouagadougou (Burkina Faso)
Guira O., Kaboré D.S.R., Dao G., Zagré N., Zohoncon T.M., Pietra V., Drabo J.Y., Simporé J.
Journal of the International Association of Providers of AIDS Care (2013) 15:3 (256-260).

The relationship between ART adherence and smoking status among HIV+ individuals
Moreno J.L., Catley D., Lee H.S., Goggin K.
AIDS and behavior (2015) 19:4 (619-625).

Predictors of Antiretroviral Adherence Self-efficacy among People Living with HIV/AIDS in a Canadian Setting
Lee W.K., Milloy M.J.S., Nosova E., Walsh J., Kerr T.
Journal of Acquired Immune Deficiency Syndromes (2019) 80:1 (103-109).

Patterns of adherence to scheduling and dietary instructions among patients on antiretroviral therapy in Pretoria, South Africa
Adefolalu A.O., Nkosi Z.Z., Olorunju S.A.S.
Transactions of the Royal Society of Tropical Medicine and Hygiene (2014) 108:9 (582-588) Article Number: tru116.

Factors associated with nonadherence to antiretroviral therapy in HIV-positive smokers
Marks King R., Vidrine D.J., Danysh H.E., Fletcher F.E., McCurdy S., Arduino R.C., Gritz E.R.
AIDS Patient Care and STDs (2012) 26:8 (479-485).

Adherence to anti-retroviral therapy and its associated factors among patients with HIV/AIDS in central Java Indonesia
Febriyanti E.K.A., Cholisoh Z., Wikantyasning E.R., Sagotra G., Anggoro I.H.M., Devientasari C., Nuraeni E.
International Journal of Pharmaceutical Research (2019) 11 Supplementry 1 (1073-1080).

The association between cigarette smoking, virologic suppression, and CD4+ lymphocyte count in HIV-Infected Russian women
Brown J.L., Winhusen T., DiClemente R.J., Sales J.M., Rose E.S., Safonova P., Levina O., Belyakov N., Rassokhin V.V.
AIDS Care - Psychological and Socio-Medical Aspects of AIDS/HIV (2017) 29:9 (1102-1106).

Adherence to antiretroviral therapy among people living with HIV/AIDS in Northeastern Brazil: A cross-sectional study
Soares R.C.A., de Brito A.M., Lima K., Lapa T.M.
Sao Paulo Medical Journal (2019) 137:6 (479-485).

Depression and coping are associated with failure of adherence to antiretroviral therapy among people living with HIV/aids
Camargo C.C., Cavassan N.R.V., Tasca K.I., Meneguin S., Miot H.A., Souza L.R.
AIDS Research and Human Retroviruses (2019) 35:11-12 (1181-1188).

Evaluation of adherence to antiretroviral treatment. Analysis of related factors
Valoración de la adherencia al tratamiento antirretroviral. Análisis de factores relacionados
Real J.M., Navarro H., Lapresta C., Rabanaque M.J., Soler E., Abad M.R.
Atencion Farmaceutica (2011) 13:4 (215-221).

Cigarette Smoking and Antiretroviral Therapy (ART) Adherence in a Sample of Heavy Drinking HIV-Infected Men Who Have Sex with Men (MSM)

Cioe P.A., Gamarel K.E., Pantalone D.W., Monti P.M., Mayer K.H., Kahler C.W.
AIDS and behavior (2017) 21:7 (1956-1963).

Association between smoking, crack cocaine abuse and the discontinuation of combination antiretroviral therapy in recife, pernambuco, Brazil
Associação entre tabagismo e o uso de crack com a descontinuidade da terapia antirretroviral combinada em Recife, Pernambuco, Brasil
Batista J.L., de Albuquerque M.F.P.M., Santos M.L., Miranda-Filho D.B., Lacerda H.R., Maruza M., Moura L.V., Coimbra I., Ximenes R.A.A.
Revista do Instituto de Medicina Tropical de Sao Paulo (2014) 56:2 (127-132).

HIV treatment adherence and unprotected sex practices in people receiving antiretroviral therapy
Kalichman S.C., Rompa D.
Sexually Transmitted Infections (2003) 79:1 (59-61).

Vitamin A and beta-carotene concentrations in adults with HIV/AIDS on highly active antiretroviral therapy
Kaio D.J., Rondó P.H.C., Souza J.M.P., Firmino A.V., Luzia L.A., Segurado A.A.
Journal of Nutritional Science and Vitaminology (2013) 59:6 (496-502).

The Impact of Substance Use on Adherence to Antiretroviral Therapy Among HIV-Infected Women in the United States
Zhang Y., Wilson T.E., Adedimeji A., Merenstein D., Milam J., Cohen J., Cohen M., Golub E.T.
AIDS and behavior (2018) 22:3 (896-908).

Determinants of discontinuation of initial highly active antiretroviral therapy regimens in a US HIV-infected patient cohort
Yuan Y., L'Italien G., Mukherjee J., Iloeje U.H.
HIV Medicine (2006) 7:3 (156-162).

Substance Use, Violence, and Antiretroviral Adherence: A Latent Class Analysis of Women Living with HIV in Canada
Carter A., Roth E.A., Ding E., Milloy M.-J., Kestler M., Jabbari S., Webster K., de Pokomandy A., Loutfy M., Kaida A.
AIDS and behavior (2018) 22:3 (971-985).

HIV-infected individuals who use alcohol and other drugs, and virologic suppression
Nolan S., Walley A.Y., Heeren T.C., Patts G.J., Ventura A.S., Sullivan M.M., Samet J.H., Saitz R.
AIDS Care - Psychological and Socio-Medical Aspects of AIDS/HIV (2017) 29:9 (1129-1136).

High-intensity cannabis use and adherence to antiretroviral therapy among people who use illicit drugs in a Canadian setting
Slawson G., Milloy M.-J., Balneaves L., Simo A., Guillemi S., Hogg R., Montaner J., Wood E., Kerr T.
AIDS and behavior (2015) 19:1 (120-127).

Gender differences in non-adherence among Brazilian patients initiating antiretroviral therapy
Bonolo P.F., Ceccato M.G.B., Rocha G.M., Acúrcio F.A., Campos L.N., Crosland Guimarães M.D.
Clinics (2013) 68:5 (612-620).

Patterns and predictors of antiretroviral therapy use among alcohol drinkers at HIV clinics in Tshwane, South Africa
Kekwaletswe C.T., Morojele N.K.
AIDS Care - Psychological and Socio-Medical Aspects of AIDS/HIV (2014) 26 Supplement 1 (S78-S82).

Patterns and predictors of antiretroviral therapy use among alcohol drinkers at HIV clinics in Tshwane, South Africa
Kekwaletswe C.T., Morojele N.K.
AIDS Care - Psychological and Socio-Medical Aspects of AIDS/HIV (2014) 26:SUPPL. 1 (78-82).

Vulnerability and non-adherence to antiretroviral therapy among HIV patients, Minas Gerais State, Brazil
De Fátima Bonolo P., Machado C.J., César C.C., Das Graças Braga Ceccato M., Guimarães M.D.C.
Cadernos de Saude Publica (2008) 24:11 (2603-2613).

Relationship between Single Tablet Antiretroviral Regimen and Adherence to Antiretroviral and Non-Antiretroviral Medications among Veterans' Affairs Patients with Human Immunodeficiency Virus
Yager J., Faragon J., McGuey L., Hoye-Simek A., Hecox Z., Sullivan S., Neubert S., Patel N.
AIDS Patient Care and STDs (2017) 31:9 (370-376).

Adherence to antiretroviral therapy, stigma and behavioral risk factors in HIV-infected adolescents in Asia
Prasitsuebsai W., Sethaputra C., Lumbiganon P., Hansudewechakul R., Chokephaibulkit K., Truong K.H., Nguyen L.V., Mohd Razali K.A., Nik Yusoff N.K., Fong M.S., Teeraananchai S., Ananworanich J., Durier N.
AIDS Care - Psychological and Socio-Medical Aspects of AIDS/HIV (2018) 30:6 (727-733).

Tobacco smoking and alcohol drinking among HIV infected people using antiretroviral therapy
Bhatta D.N., Subedi A., Sharma N.
Tobacco Induced Diseases (2018) 16:April.

Correlates of non-adherence to antiretroviral therapy in a cohort of HIV-positive drug users receiving antiretroviral therapy in Hanoi, Vietnam
Jordan M.R., Obeng-Aduasare Y., Sheehan H., Hong S.Y., Terrin N., Duong D.V., Trung N.V., Wanke C., Kinh N.V., Tang A.M.
International Journal of STD and AIDS (2014) 25:9 (662-668).

Baseline Cigarette Smoking Status as a Predictor of Virologic Suppression and CD4 Cell Count During One-Year Follow-Up in Substance Users with Uncontrolled HIV Infection
Winhusen T., Feaster D.J., Duan R., Brown J.L., Daar E.S., Mandler R., Metsch L.R.
AIDS and behavior (2018) 22:6 (2026-2032).

Substance Use Stigma and Antiretroviral Therapy Adherence among a Drug-Using Population Living with HIV
Stringer K.L., Marotta P., Baker E., Turan B., Kempf M.-C., Drentea P., Stepanikova I., Turan J.M.
AIDS Patient Care and STDs (2019) 33:6 (282-293).

Factors associated with adherence amongst 5295 people receiving antiretroviral therapy as part of an international trial
O'Connor J.L., Gardner E.M., Mannheimer S.B., Lifson A.R., Esser S., Telzak E.E., Phillips A.N.
Journal of Infectious Diseases (2013) 208:1 (40-49).

A Longitudinal Study of Behavioral Risk, Adherence, and Virologic Control in Adolescents Living with HIV in Asia
Ross J.L., Teeraananchai S., Lumbiganon P., Hansudewechakul R., Chokephaibulkit K., Khanh T.H., Van Nguyen L., Mohamed T.A.J., Yusoff N.K.N., Fong M.S., Prasitsuebsai W., Sohn A.H., Kerr S.J.
Journal of Acquired Immune Deficiency Syndromes (2019) 81:2 (e28-e38).

HIV-infected adolescents have low adherence to antiretroviral therapy: A cross-sectional study in Addis Ababa, Ethiopia
Firdu N., Enquselassie F., Jerene D.
Pan African Medical Journal (2017) 27 Article Number: 80.

Characteristics of adolescents living with HIV receiving care and treatment services in antiretroviral therapy clinics in Cambodia: descriptive findings from a cross-sectional study
Yi S., Tuot S., Pal K., Khol V., Sok S., Chhoun P., Ferguson L., Mburu G.
BMC health services research (2018) 18:1 (781).

Non-adherence to anti-retroviral therapy among HIV infected adults in Mon State of Myanmar
Aye W.L., Puckpinyo A., Peltzer K.
BMC public health (2017) 17:1 (391).

High-risk behaviours, and their associations with mental health, adherence to antiretroviral therapy and HIV parameters, in HIV-positive men who have sex with men

Pool E.R.M., Winston A., Bagkeris E., Vera J.H., Mallon P.W.G., Sachikonye M., Post F.A., Pozniak A., Boffito M., Anderson J., Williams I., Johnson M., Burgess L., Sabin C.A., Babalis D., Garvey L., Campbell L., Yurdakul S., Okumu S., Pollard L., Suárez B.S., Otiko D., Phillips L., Laverick R., Beynon M., Salz A.-L., Severn A., Fisher M., Clarke A., Richardson C., Kirk S., Gleig R., Macken A., Ghavani-Kia B., Maher J., Byrne M., Flaherty A., Babu S., Mguni S., Clark R., Nevin-Dolan R., Pelluri S., Ngwu N., Hemat N., Carroll A., Kinloch S., Youle M., Madge S., Whitehouse A., Babalis D., Garvey L., Underwood J., Tembo L., Stott M., McDonald L., Dransfield F., Bracchi M., Pagani N., Cerrone M., Bradshaw D., Ferretti F., Higgs C., Seah E., Fletcher S., Anthonipillai M., Moyes A., Deats K., Syed I., Matthews C., Fernando P., Chiwome C., Hardwick S., De Francesco D.
HIV Medicine (2019) 20:2 (131-136).

Alcohol consumption among HIV-infected women: Impact on time to antiretroviral therapy and survival
Neblett R.C., Hutton H.E., Lau B., McCaul M.E., Moore R.D., Chander G.
Journal of Women's Health (2011) 20:2 (279-286).

Adherence to antiretroviral therapy among HIV/ AIDS patients in the context of early treatment initiation in Vietnam
Mai H.T., Le G.M., Tran B.X., Do H.N., Latkin C.A., Nguyen L.T., Thai T.P.T., Le H.T., Ngo A.T., Nguyen C.T., Ho C.S.H., Ho R.C.M.
Patient Preference and Adherence (2018) 12 (2131-2137).

Prevalence and correlates of substance use among youth living with HIV in clinical settings
Gamarel K.E., Brown L., Kahler C.W., Fernandez M.I., Bruce D., Nichols S.
Drug and Alcohol Dependence (2016) 169 (11-18).

Cigarette Smokers are Less Likely to Have Undetectable Viral Loads: Results from Four HIV Clinics
Cropsey K.L., Willig J.H., Mugavero M.J., Crane H.M., McCullumsmith C., Lawrence S., Raper J.L., Christopher Mathews W., Boswell S., Kitahata M.M., Schumacher J.E., Saag M.S.
Journal of Addiction Medicine (2016) 10:1 (13-19).
